# Indonesian marine and its medicinal contribution

**DOI:** 10.1007/s13659-023-00403-1

**Published:** 2023-10-16

**Authors:** Ari Satia Nugraha, Lilla Nur Firli, Dinar Mutia Rani, Ayunda Hidayatiningsih, Nadya Dini Lestari, Hendris Wongso, Kustiariyah Tarman, Ayu Christien Rahaweman, Jeprianto Manurung, Ni Putu Ariantari, Adelfia Papu, Masteria Yunovilsa Putra, Antonius Nugraha Widhi Pratama, Ludger A. Wessjohann, Paul A. Keller

**Affiliations:** 1 Drug Utilisation and Discovery Research Group, Faculty of Pharmacy, Universitas Jember, Jember, 68121 Indonesia; 2 Research Center for Radioisotope, Radiopharmaceutical, and Biodosimetry Technology, Research Organization for Nuclear Energy, National Research and Innovation Agency, Puspiptek, Banten, 15314 Indonesia; 3 Research Collaboration Center for Theranostic Radiopharmaceuticals, National Research and Innovation Agency, J1. Raya Bandung-Sumedang KM 21, Sumedang, 45363 Indonesia; 4 Department of Aquatic Product Technology, Faculty of Fisheries and Marine Sciences; and Division of Marine Biotechnology, Centre for Coastal and Marine Resources Studies (CCMRS), IPB University, Bogor, 16680 Indonesia; 5 Leibniz Institute Für Pflanzenbiochemie, Weinberg 3, 06120 Halle (Saale), Germany; 6 German Centre for Integrative Biodiversity Research (iDiv) Halle-Jena-Leipzig, Puschstrasse 4, 04103 Leipzig, Germany; 7 Department of Pharmacy, Faculty of Mathematics and Natural Sciences, Udayana University, Badung, Bali, 80361 Indonesia; 8 Biology Department, Faculty of Mathematics and Natural Sciences, Sam Ratulangi University, Manado, 95115 Indonesia; 9 Vaccine and Drug Research Center, National Research and Innovation Agency, Cibinong, Jawa Barat 16911 Indonesia; 10 School of Chemistry and Molecular Biosciences, Molecular Horizons, University of Wollongong, Wollongong, NSW 2522 Australia

**Keywords:** Indonesian marine, Laulimalide, Papuamine, Manzamine A, Manadoperoxide B, Hippuristanol, (+)-8-Hydroxymanzamine A

## Abstract

The archipelagic country of Indonesia is populated by the densest marine biodiversity in the world which has created strong global interest and is valued by both Indigenous and European settlements for different purposes. Nearly 1000 chemicals have been extracted and identified. In this review, a systematic data curation was employed to collate bioprospecting related manuscripts providing a comprehensive directory based on publications from 1988 to 2022. Findings with significant pharmacological activities are further discussed through a scoping data collection. This review discusses macroorganisms (Sponges, Ascidian, Gorgonians, Algae, Mangrove) and microorganism (Bacteria and Fungi) and highlights significant discoveries, including a potent microtubule stabilizer laulimalide from *Hyattella *sp.*,* a prospective doxorubicin complement papuamine alkaloid from *Neopetrosia cf exigua*, potent antiplasmodial manzamine A from *Acanthostrongylophora ingens*, the highly potent anti trypanosomal manadoperoxide B from *Plakortis cfr.* Simplex, mRNA translation disrupter hippuristanol from *Briareum *sp, and the anti-HIV-1 (+)-8-hydroxymanzamine A isolated from *Acanthostrongylophora *sp. Further, some potent antibacterial extracts were also found from a limited biomass of bacteria cultures. Although there are currently no examples of commercial drugs from the Indonesian marine environment, this review shows the molecular diversity present and with the known understudied biodiversity, reveals great promise for future studies and outcomes.

**Graphical Abstract:**

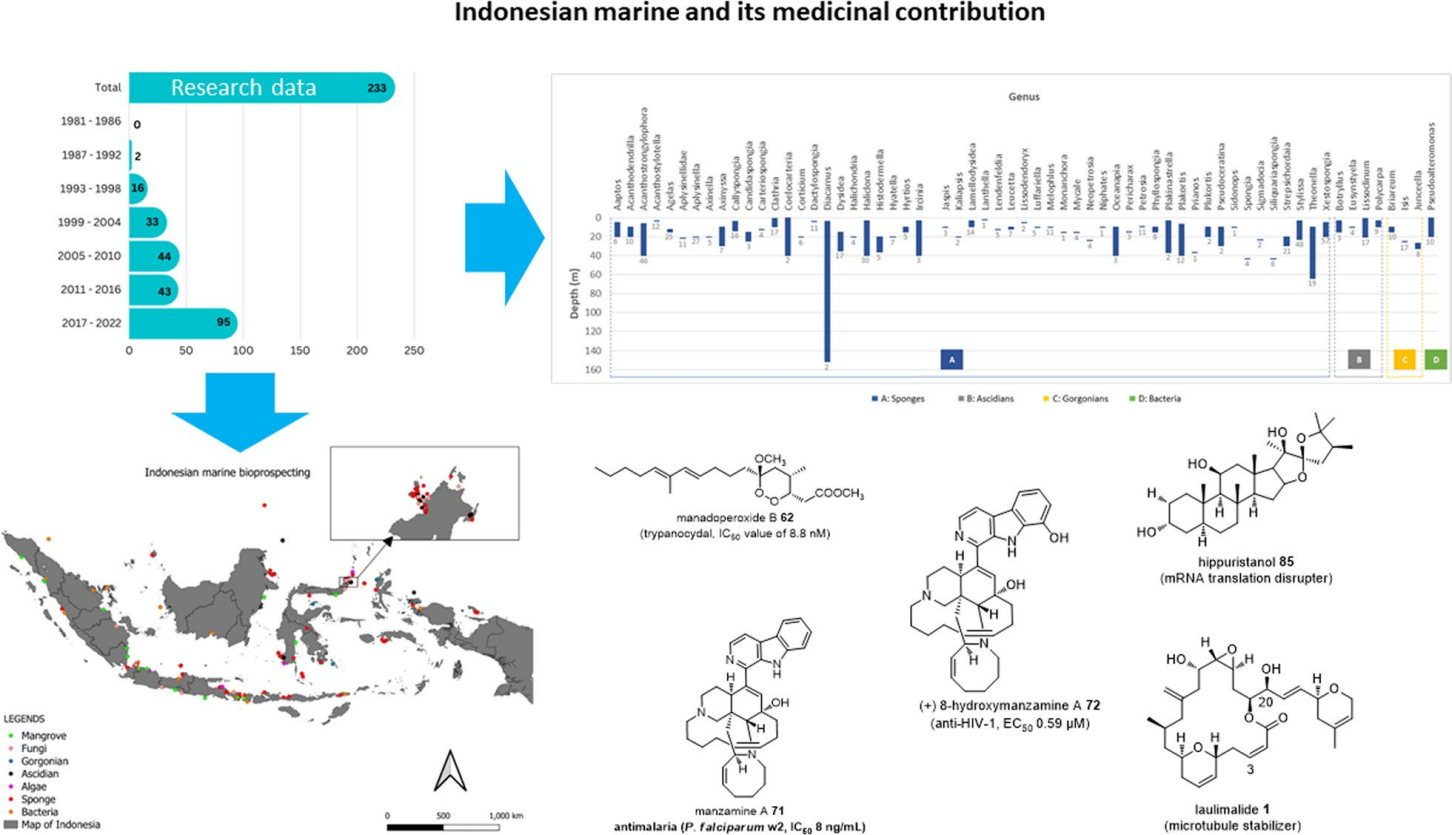

**Supplementary Information:**

The online version contains supplementary material available at 10.1007/s13659-023-00403-1.

## Introduction

Indonesia is the largest archipelagic country in the world comprised of 17,504 islands with a total surface area of ~ 6.32 million km^2^ surrounded by 0.3 million km^2^ of territorial sea, 3.09 million km^2^ archipelagic water, 2.97 million km^2^ executive economic zone and 2.01 million km^2^ of land in which is bordered by 99,093 km coastline [1]. The island nation formed through tectonic movement between the Asian and Australian continents resulting in an archipelago rich in biodiversity. Indonesian waters stretch across three different time zones being host to 86,700 square kilometres of coral reefs between the Pacific and Atlantic oceans [2]. The extensive marine biodiversity supplies 70% of dietary protein to the Indonesian population and generates an annual revenue of approximately USD 10 billion. During the kingdom era in the early civilisation of the archipelago, Srivijaya Budish Kingdom (7th–12th AD) was a respected ruler in the South East Asian region controlling international trade between the East and West through the Malacca Straits. Srivijaya international perpetuation was attenuated by Hindu Majapahit Kingdom (12th–15th AD) [3]. These Kingdoms were acknowledged as maritime powers due to the strength of their navy in this territory [3]. The indigenous people of the archipelago inherited medicinal knowledge using both terrestrial and marine based medicaments which have been passed through generations mostly through verbal communication [4, 5]. For example, traditional sea cucumber diving by the Maccassan people established a traditional practice which escalated into the northern territory of Australia, due to the commodity value [6]. Traditional sea salt makers by the Balinese recorded their reliance on high quality salts throughout centuries [5]. Nevertheless, ethnopharmacological data in marine-based traditional medicines are less in number than their terrestrial counterparts, emphasising the opportunity for greater examination.

**Fig. 1:**
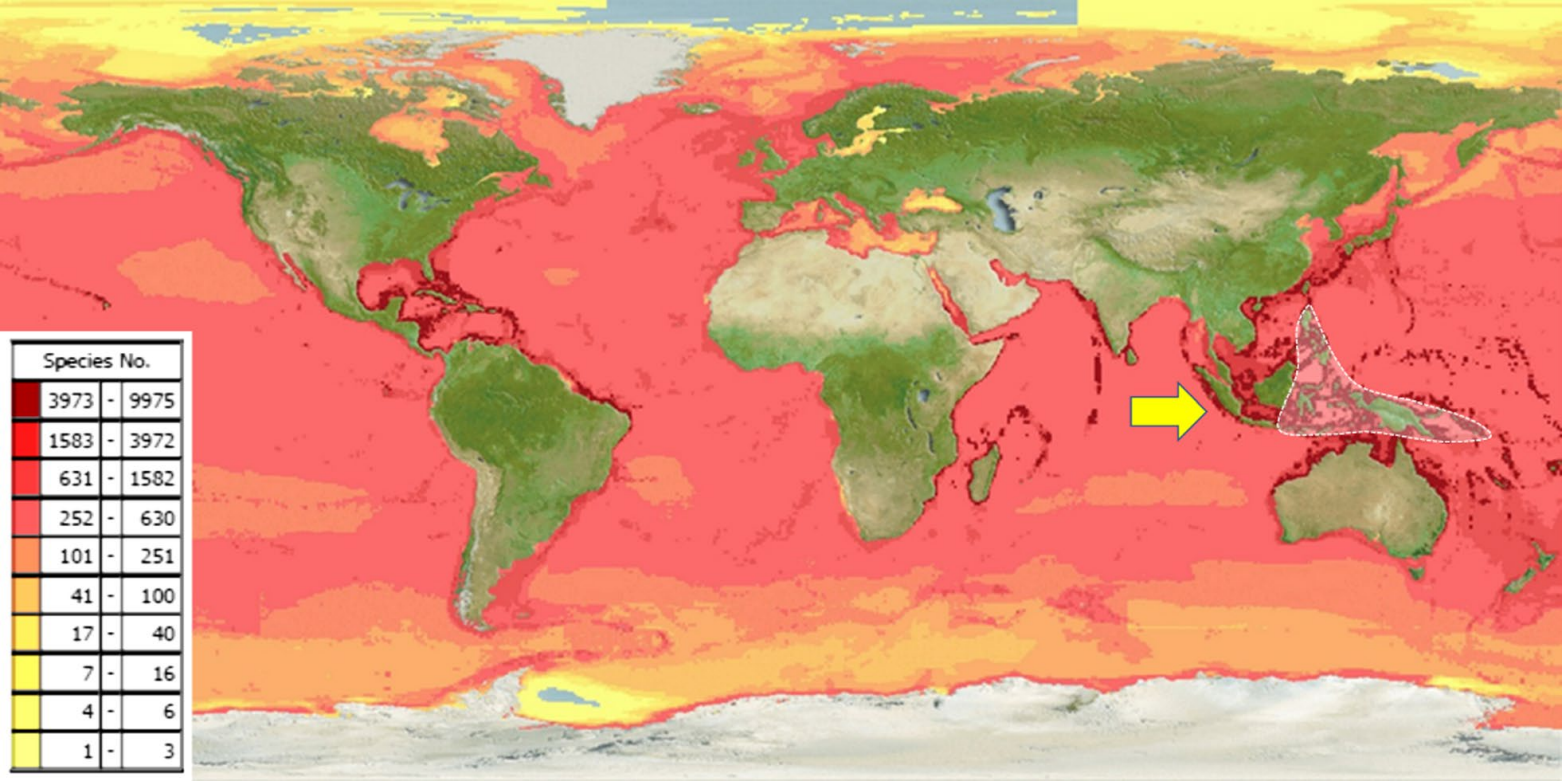
Predicted range maps for aquatic species across the world and the world epicentre of coral reef in Indonesia(see arrow) [7]

The Indonesian archipelago is located in the epicentre of the coral triangle and is over 25,000 km^2^ and is home to more than 75% of the world’s coral reef species and extensive mangrove habitats due to the extended coastline (Fig. 1) [7]. Unfortunately, multi sector conflicts of interest have contributed to the deterioration of the coral reef resulting in biodiversity depletion, with coral reef status monitoring indicating a worse reef shape condition in eastern Indonesia despite a less dense population [8]. Several activities including unsustainable fishing (i.e. blast fishing), and coral exploitation for building materials were the major causes. The problem led to the establishment of the Coral Triangle Initiative (CTI) in 2009 with oversight on coral reefs, fisheries and food security. It involved six countries, Indonesia, Malaysia, Papua New Guinea, Philippines, Solomon Islands and Timor-Leste in agreeing to address urgent threats facing the coastal and marine resources of the Coral Triangle region, an area of approximately 2.3 million square miles [9]. Additional Indonesian government initiatives have reinforced aquatic conservation areas from marine national parks to marine wildlife and managed the Indonesian marine into Core, Sustainable Fisheries and Utilization Zones [1].

Early examination of Indonesian marine organisms dates back to the Dutch East Indies European Settlements and focussed mostly on ecological descriptions – this included a Danish expedition in the waters surrounding the Ambon islands in 1845–1847 [10]. This was the period of golden age of species curation with taxonomically driven exploration where the numbers of species and their distribution across the phylogeny tree was the focus. Despite these studies, the first compound isolated from the Indonesian marine environment was published in 1973 by Rothberg et al. reporting a sapogenin (23ξ-acetoxy-17-deoxy-7,8,-dihydroholothurinogenin) which was a triterpenoid from the dried skins of the sea cucumber, *Stichopus chloronotus* Brandt, collected in the bay of Telukdalam, Nias Island with no pharmacological evaluation performed [11].

There are few published reviews on Indonesian marine natural products with one only reporting on marine invertebrates, sponges, tunicate and soft corals collected from 2007 to 2020 [12], with another covering a limited dataset curated from 1970 to 2017 which includes marine natural products distributed across 94 species [13], while another discusses only marine organohalogen compounds [14]. All these manuscripts offer limited and specific commentary and fail to provide a broad and comprehensive perspective of the Indonesian marine phytochemistry environment. In this current study, a systematic review covering the bioprospecting research of Indonesian marine natural products is presented, from early research in 1973 to 2022 covering macroorganisms (invertebrates, algae and marine vegetation) and microorganisms (fungi and bacteria). Compounds with significant pharmacological efficacy were selected and discussed through further scoped literature studies from similar compounds isolated from other organisms across the globe.

## Methodology and general information

**Fig. 2:**
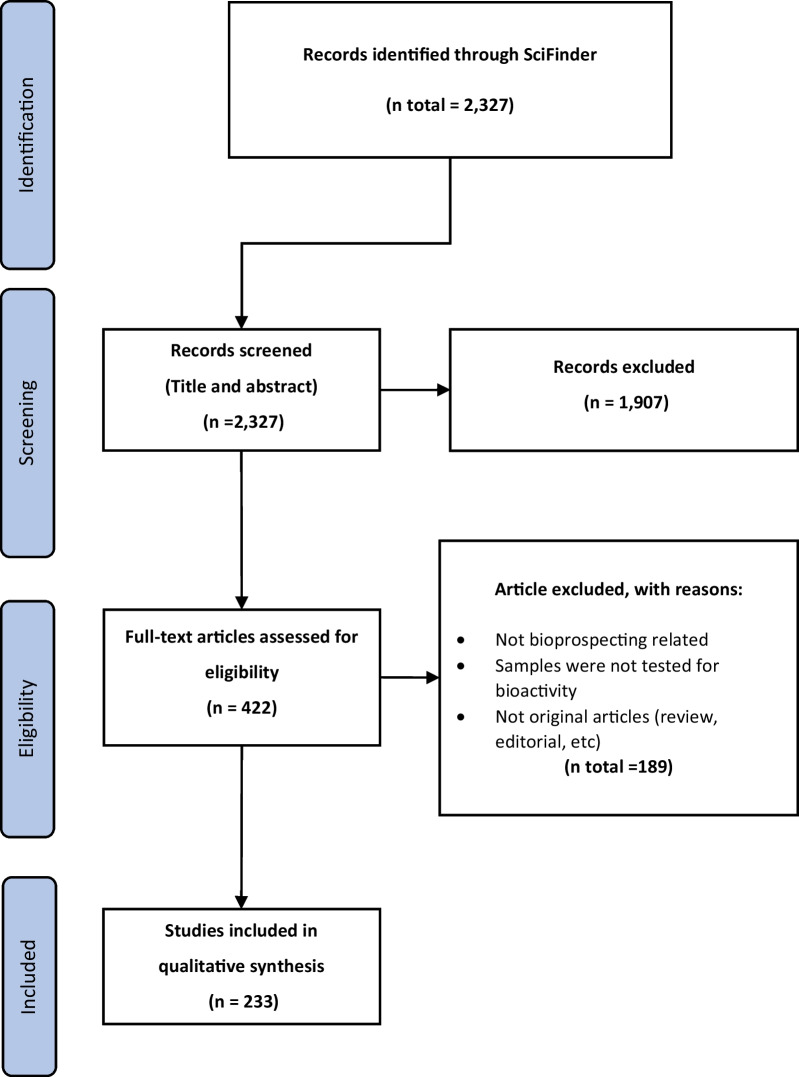
Systematic data curation of Indonesian marine natural products

The SciFinder database was utilised using the keywords Indonesia, marine, sea, ocean, sponge, ascidian, gorgonian, fungi, bacteria, mangrove for initial data mining. Non-medicinal related publications were excluded leaving chemical and pharmacological evaluation articles. Information on the species includes geographical sampling location, chemical constituents, and pharmacological information were tabulated and compounds with potential activities were selected for discussion using compounds names as keywords for further scoping searches. Figure 2 summarises this methodology.

**Fig. 3:**
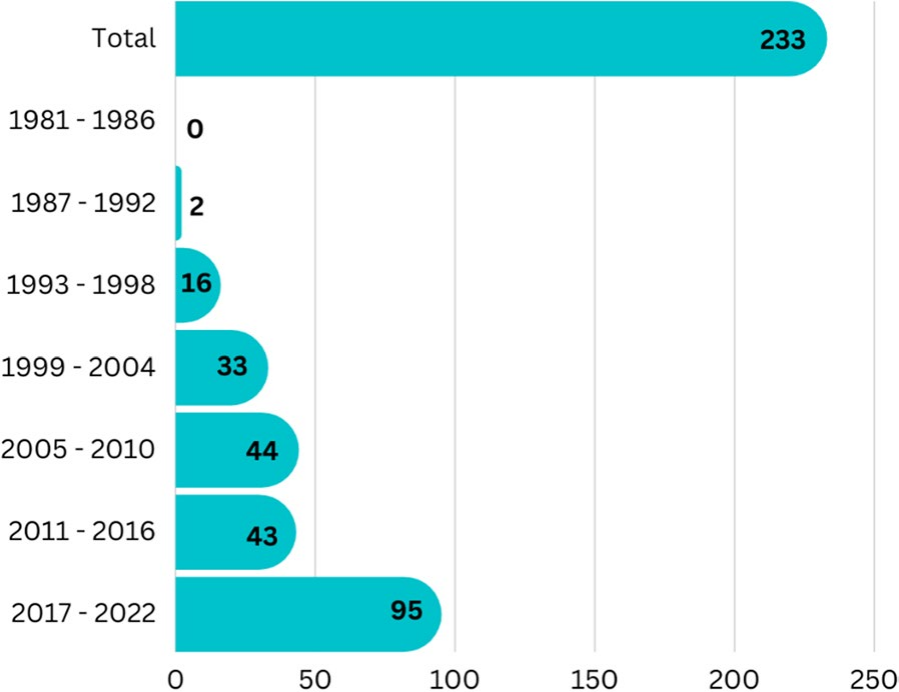
Indonesian marine natural products manuscripts distribution throughout the years

**Fig. 4:**
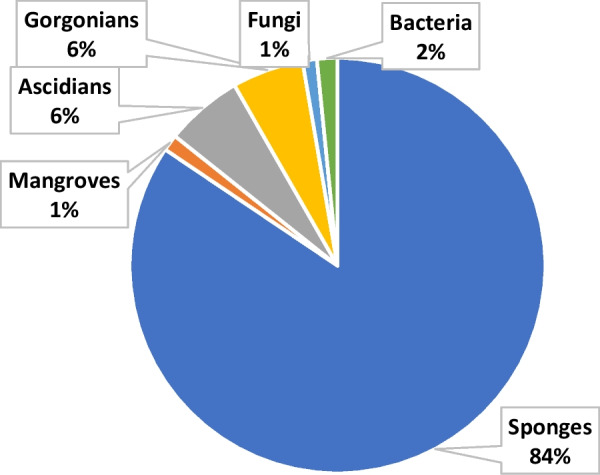
Indonesian marine natural products discovered across organism types

**Fig. 5:**
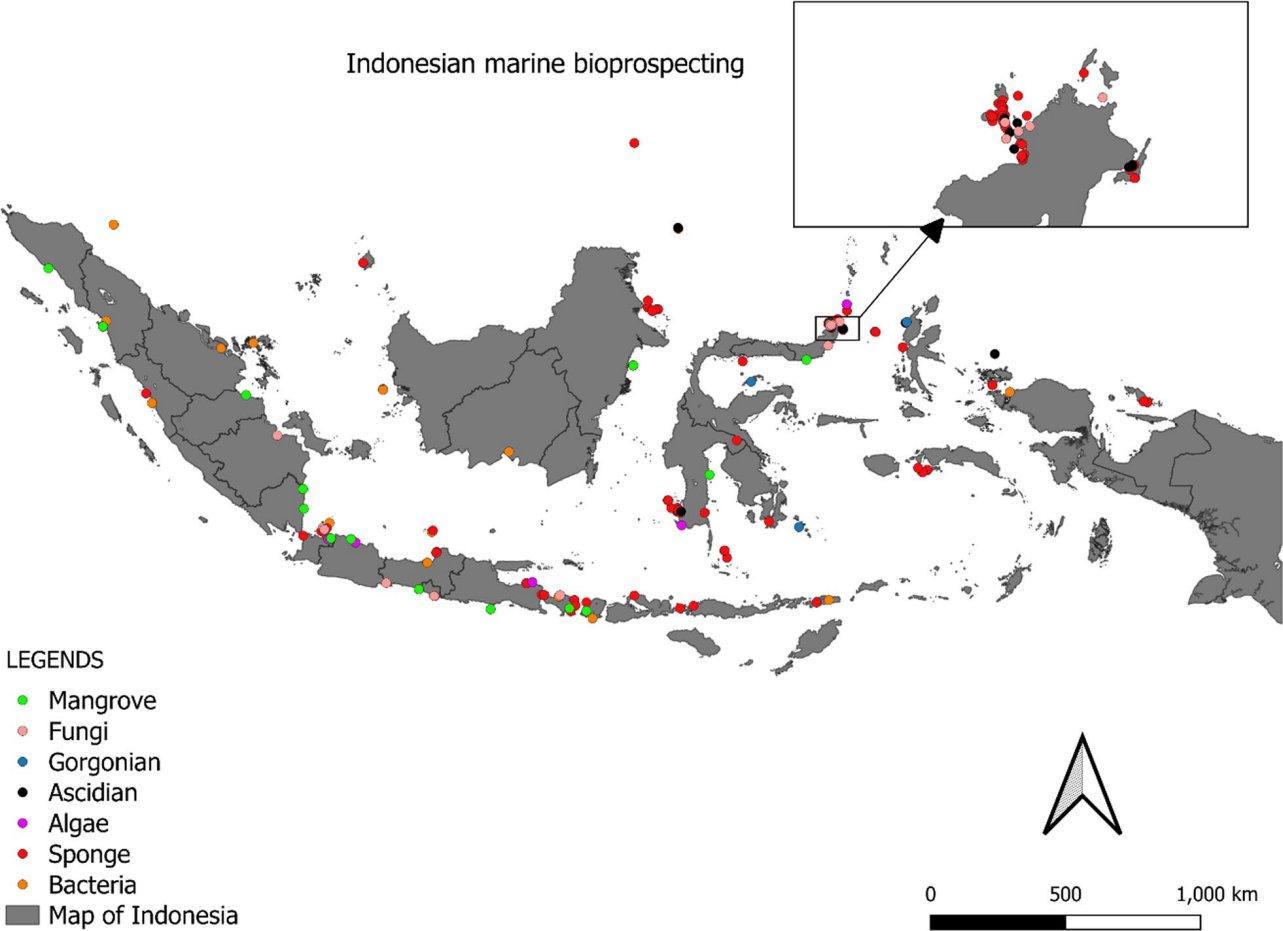
Sample location of Indonesian marine bioprospecting

**Fig. 6:**
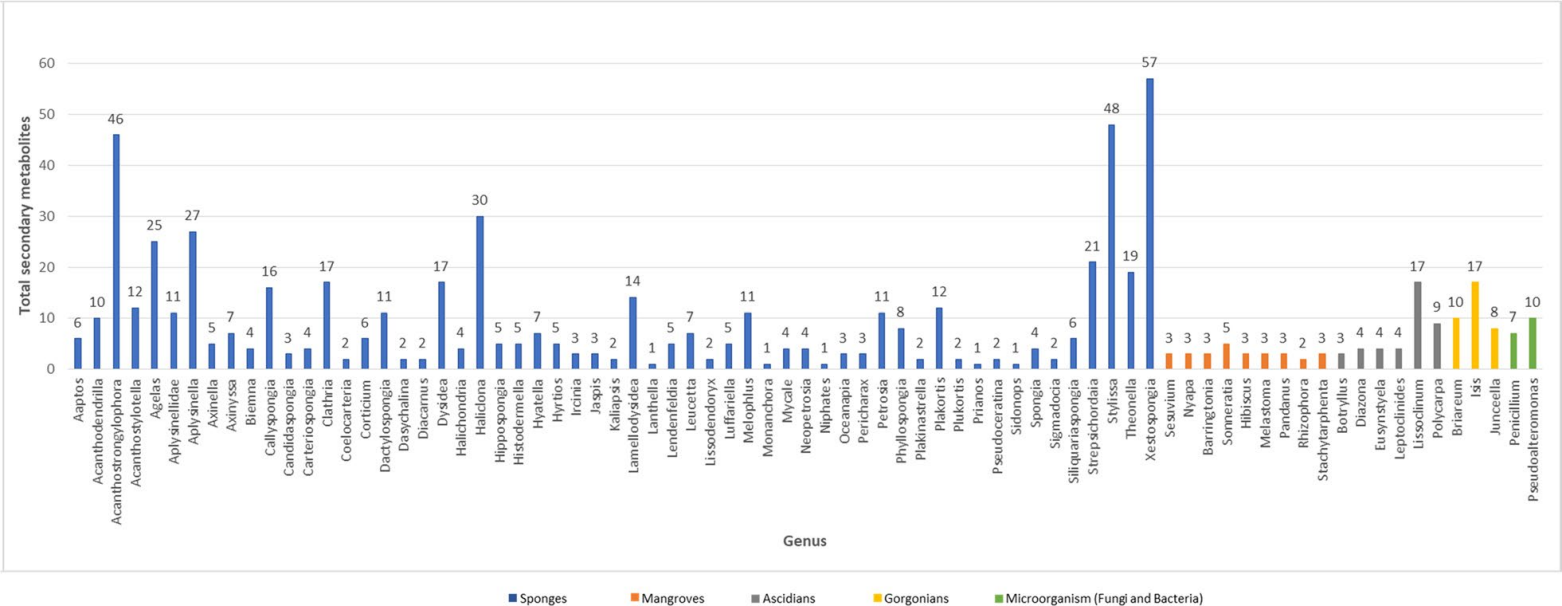
Distribution of compounds across genus

**Fig. 7:**
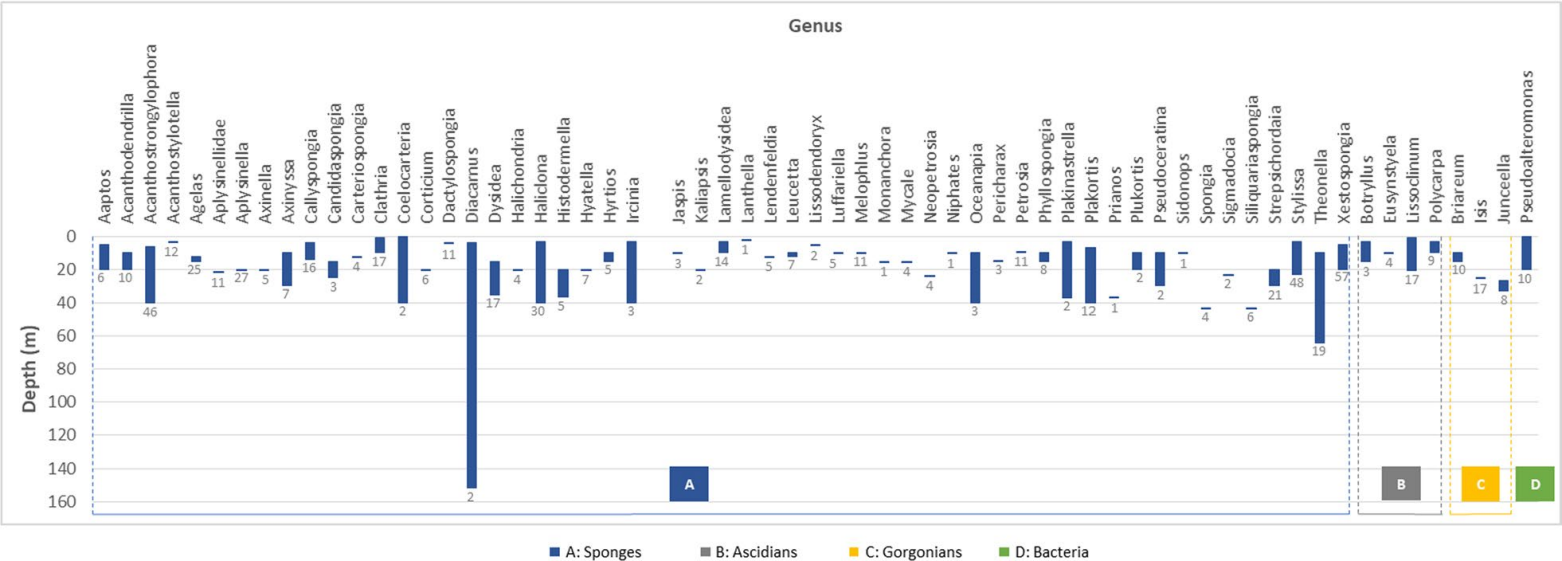
Number of compounds and the depth of sample collection across genus

The data were tabulated in separate tables for each type of organism, sponge, ascidian, gorgonian, algae, mangrove, fungi, bacteria (Additional file 1: Table S1 to S7, respectively). The data curation profile indicated a strong interest in Indonesian marine natural products in the last 5 years (Fig. 3). This work depicted a positive signal in the implementation of Nagoya protocol ensuring mutual benefits with Indonesian marine bioprospecting. As a result, a total of 630 secondary metabolites were isolated from Indonesian marine organisms with sponges providing the majority, likely due to its higher biomass availability and accessibility (Fig. 4). The Bunaken Marine National Park and surround remain the hotspot for marine sample collection (Fig. 5). Exploration on genera *Acanthostrongylophora, Haliclona, Stylissa, Xestospongia* produced the largest number of secondary metabolites totaling more than 30 compounds (Fig. 6). Most of the samples were collected from sites less than 20 m depth with only *Diacarnus* being collected from the deep sea which provided only two isolated compounds. (Fig. 7).

## Indonesian marine macro organisms

### Sponges

Sponges are animals from the Porifera phylum and are distributed from polar to tropical ocean environments and are one of the most abundant marine invertebrates. There are over 8500 identified species in the world with 850 recorded in Indonesian waters.[15–17] Sponges are distributed in a range of different ecosystems and are found in both shallow and deep sea water [18, 19]. The successful chemical study of sponges gave rise to a global escalation in sponge bioprospecting resulting in the number of studied species and the resulting isolated molecules outnumbering those from other marine organisms. The study of Indonesian marine sponges was conducted mostly in the eastern part of Indonesia including the Spermonde Archipelago, Sulawesi, North Sulawesi, Derawan Islands, Kalimantan, and Wakatobi waters [20]. There are approximately 9500 novel compounds that have been isolated from marine sponges in the period 1950 to 2019 [21] and 732 secondary metabolites isolated from Indonesian marine sponges from 1970 to 2017 [13]. Alkaloids became the most abundant group of secondary metabolites isolated from sponges, along with other groups including terpenes, peptides, and polyketides. Steroid and saponin were also found as sponge secondary metabolites, but only in small quantities. The recent studies conducted by Ohte et al. and Murtihapsari et al. in 2021 reported two novel compounds, a new sesquiterpene lactone, bicyclolamellolactone A, isolated from *Lamellodysidea* sp. (cf. *L. herbacea*) and a new sterol, kaimanol, isolated from *Xestospongi*a sp, respectively [22, 23].

Sponges have provided numerous biologically active compounds with activities against various diseases including cancer, microbial infections, and malaria. The current (1985 to 2012) statistical study of marine natural products bioactivity reported 56% of the 4196 biogically active compounds from sponges are known for their anticancer activity against an array of cell lines [24]. Reports on the Indonesian marine-originated invertebrates were previously reviewed by Putra et al. which covered studies published throughout 2007–2020 [12]. In this current study, early reports from the 1970s to 2022 were collated to provide a comprehensive dataset (Additional file 1: Table S1). In particular, compounds isolated from Indonesian sponges were found to possess significant activities as anti-degenerative agents, anti-cancer and anti-infective agents.

#### Anti-degenerative disease agents

**Fig. 8:**
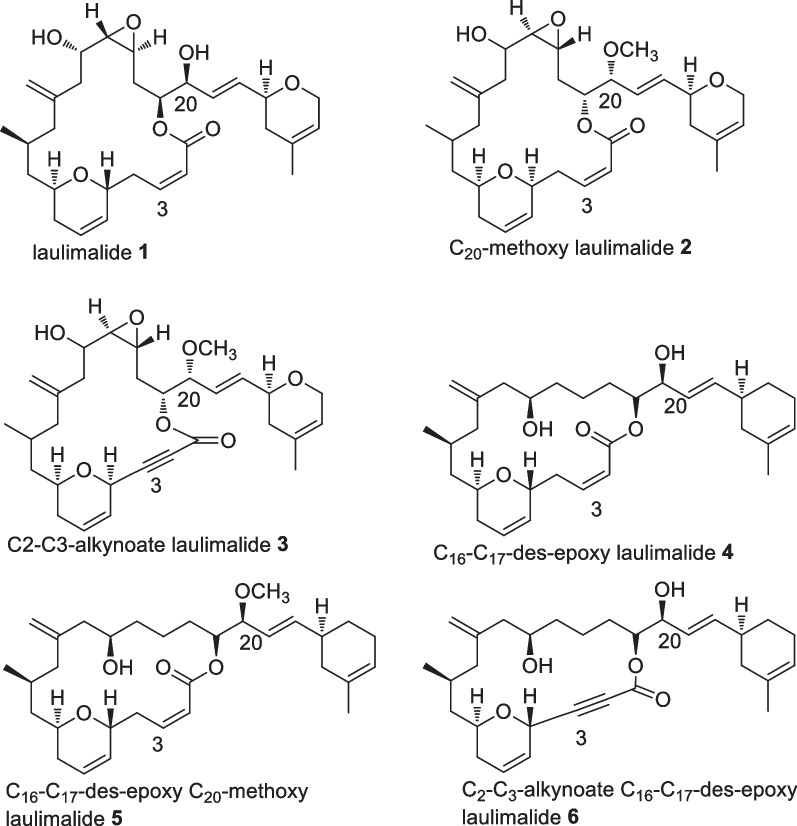
Laulilamide and its derivatives for QSAR studies

**Fig. 9:**
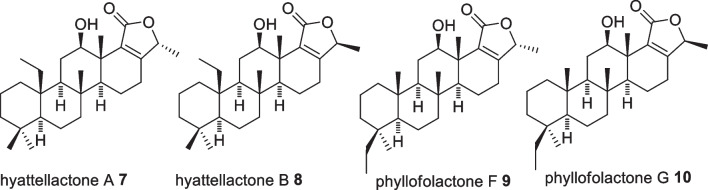
Hyttellactones from Indonesian *Hyattella *sp.

The first investigated sponge from Indonesian waters was *Hyattella sp*, collected near Manado in the 1980s, which yielded 1.5% of Laulimalide **1** (Fig. 8), a potent anticancer initially displaying activity against papilloma KB cell line with an IC_50_ value of 15 ng/mL [25]. It has antimitotic activity through a microtubule stabilizing mechanism, with a novel binding site on the external face, rather than on the luminal face of the *β* tubulin subunit of lineaging stabilizing agents, TAK-sayn, allowing for synergism [26, 27]. Laulimalide **1** non mitotic activity includes inhibiting cell migration and endothelial tubule formation [26]. In addition, the macrolide indicated a potent kinase inhibitory activity and induced multidrug transporter P-glycoprotein expression [27, 28]. Several molecular optimisations were attempted (**2**–**6**) including structural stabilisation through removal of the epoxy functionality (**4**—**6**) which reduced its potency by 11 to 50 fold without altering the molecular mechanism (Fig. 8) [29–31]. Despite the lengthy synthetic routes, derivatives were produced in sufficient quantities to facilitate in vivo studies which showed good pharmacokinetic outcomes, however severe toxicity emerged thus requiring further optimisation prior clinical performance experiments [26]. Studies on the same species produced four sesquiterpenes derivatives, hyttellactones A **7**, B **8**, phyllofolactone F **9** and G **10** with no antiproliferative activities (Fig. 9). Hyttellactones A **7** and F **9** showed potent efficacy as PTP1B inhibitory agents (therapeutic target for diabetic type 2 treatment) with IC_50_ values of 7.45 and 7.47 µM, respectively. The potency of phyllfolactone G was decreased indicating that the chirality at C24 was crucial for activity of these scalarane sesterterpenes [32].

**Fig. 10:**
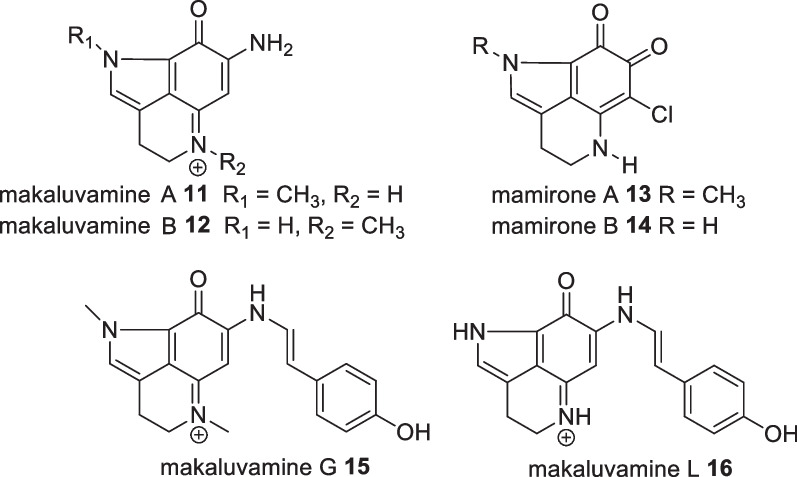
Makaluvamine derivatives from Indonesian *Histodermella *sp.

The tryptamine-tyramine-derived alkaloid, makaluvamine G **15**, along with four other alkaloids (makaluvamines A **11**, C **12** and damirones A **13**, B **14**), was isolated from the marine invertebrate *Histodermella sp*, collected in Manado Bay-Sulawesi Island. Only makaluvamine G **15** possessed moderate anticancer activity with an IC_50_ value of 500 ng/mL against P388, A549, HT-29, and MCF-7 with an IC_50_ value of 350 ng/mL against KB cell lines (Fig. 10). No significant activity was displayed against several fungi [33]. Makaluvamines contain a pyrrolo[4,3,2-*d,e*]quinoline scaffold and are known for their high cytotoxicity. Further alkaloids of sponge origin include the discorhabdins, epinardins, batzellines, isobatzellines, makaluvamines, tsitsikammamines and veiutamine skeletons [34]. However, makaluvamine G **15** was reported to moderately inhibit DNA replication on topoisomerase I [35]. The absence of a methyl substituent on the amine group of makaluvamine L **16** resulted in an order of magnitude higher cytotoxicity towards HCT-116 cells [35].

**Fig. 11:**
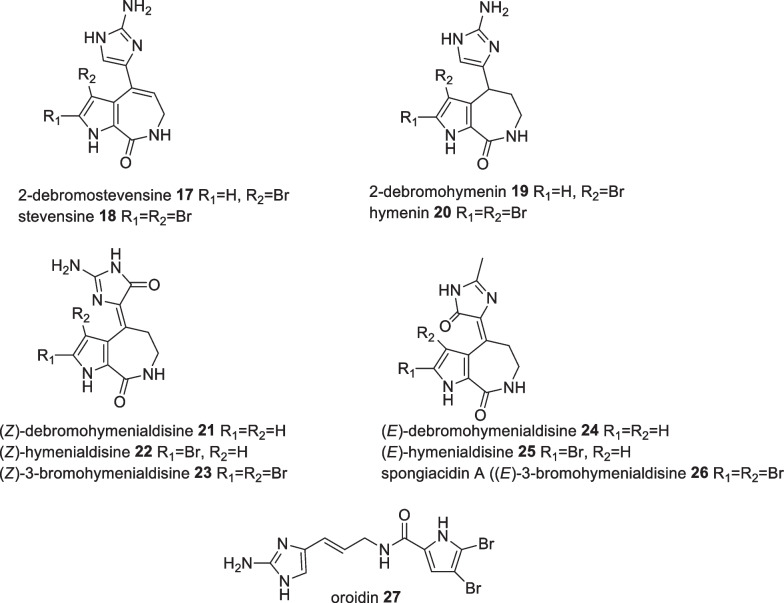
Bromopyrrole derivatives from Indonesian *Stylissa carteri*

Investigations on the indopacific sponge *Stylissa carteri*, collected off shore from Barangpolo Island, isolated eleven bromopyrrole compounds (debromostevensine **17**, stevensin **18,** debromohymenin **19**, hymenin **20**, (*Z*)-debromohymenialdisine **21**, (*Z*)-hymenialdisine **22**, (*Z*)-3-bromohymenialdisine **23**, (*E*)-debromohymenialdisine **24**, (*E*)-hyrmenialdisine **25**, spongiacidin A **26**, oroidin **27**) in which zwitterionic mesomerism allowed conversion of (*Z*)-debromohymenialdisine **21** towards its (*E*) isomer (Fig. 11). This instability limited cytotoxic evaluation of the pyrrole alkaloids leaving (*Z*)-hymenialdisine **22** to possess the most significant activity against MONO-MAC-6 cell with an IC_50_ value of 200 ng/mL [36]. Further studies on other Indonesian Stylissa revealed 27 bromopyrrole alkaloids in which (*Z*)-hymenialdisine **22** showed consistent antiproliferative activity with a plausible mechanism related to CLK-1 and CDK-5 kinase protein inhibition with IC_50_ values of 0.01 µM and 0.26 µM, respectively [37]. Nineteen of these pyrrole related compounds were synthesised and evaluated for their kinases inhibitor activity with the (*Z*)-hymenialdisine **22** possessing the highest activity [38]. Preliminary SAR studies revealed alteration of the free amine on carbon 14 was not beneficial to its activity. The compound also indicated antimalarial activity based on *Plasmodium falciparum* glycogen synthase kinase-3 (*Pf*GSK-3) inhibition with an IC_50_ value of 0.2 µM in which bromination at carbon 3 escalates the activity by threefold [37].

**Fig. 12:**
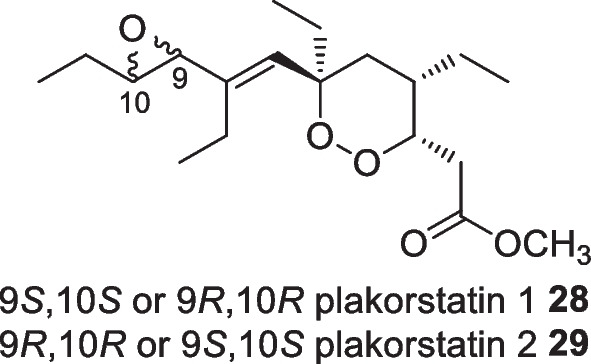
Peroxides isolated from Indonesian *Plakortis nigra*

Bio-guided assay strategies in the search for new anticancer agents from the Indonesian marine sponge, *Plakortis nigra,* isolated two peroxide containing molecules, plakorstatins 1 **28** and 2 **29** (Fig. 12). Antiproliferative screening against the cell lines, P388, BXPC3, MCF7, SF268, NCIH460, KM20L2, DU145, indicated P388 sensitivity against the peroxides with IC_50_ values of 1.2 and 0.91 µM, respectively [39]. The unique peroxide compounds were also reported from *P. simplex* from the Norwegian ocean producing three peroxide molecules although subordinate activity against human tumor cell lines was obtained [40]. No further studies on these peroxides have been reported.

**Fig. 13:**
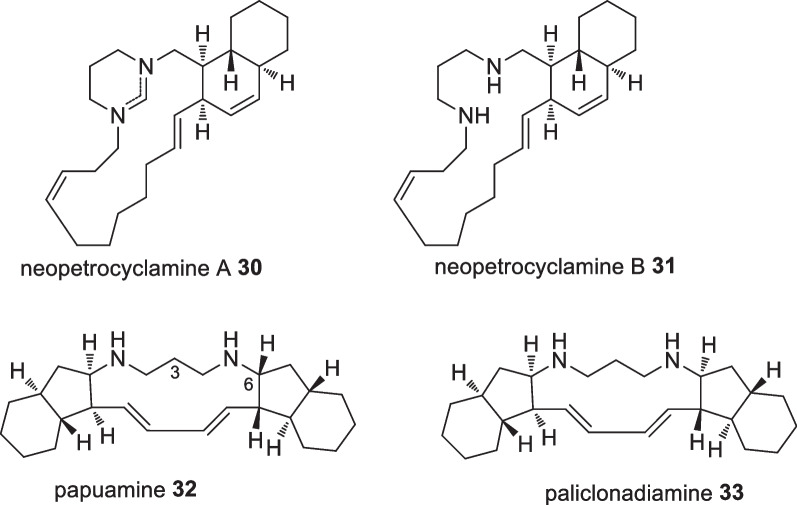
Alkaloids isolated from Indonesian *Neopetrosia cf exigua*

Liang et al explored *Neopetrosia cf exigua* sponges collected from Derawan Piers and successfully isolated four polycyclic alkaloids, neopetrocyl amines A **30** and B **31**, papuamine **32**, and haliclonadiamine **33** in which a formaminidium moiety differentiated the two neopetrocyls (Fig. 13). Anticancer evaluation against UO-31, A498 and SF-295 cell lines indicated only papuamine **32** to possess significant cytotoxicity against human glioblastoma SF-295 with a GI_50_ value of 800 nM [41]. Interestingly, a molecular comparison between papuamine **32** and holiclonadiamine **33** showed the importance of the stereogenic C-6 for its cytotoxicity. Papuamine **32** and holiclonadiamine **33** were also isolated from the sponge *Haliclona *sp. collected from a coral reef at Manado in which papuamine **32** showed moderate antineoplastic activity against MCF-7, hepatoma Huh-7, prostate cancer PC-3, HCT-15, histiocytic lymphoma U937 and Jurkat cells, with IC_50_ values of 1.39, 0.89, 1.23, 1.50, 0.93 and 1.50 µM, respectively [42]. A similar test on Holiclonadiamine **33** showed comparable IC_50_ values of 1.35, 1.13, 1.66, 4.44, 1.00 and 2.51 µM, respectively. Nevertheless, the cytotoxicity of the crude extract from *Haliclona* sp. on Caco-2 cells was 2.39 ug/mL, and was superior to the standard anticancer drug etoposide (12.0 ug/mL) and mitomycin C (2.80 ug/mL) [42]. Papuamine **32** was suggested to operate through an antiproliferative mechanism by inducing earlier onset autophagy, ATP depletion causing mitochondrial damage and c-Jun *N*-terminal kinase activation in MCF-7 cells [43]. In addition, papuamine **32** was suggested as a prospective doxorubicin modulator in chemotherapy as the alkaloid was able to activate c-Jun N-terminal kinase in human breast cancer MCF-7 cells without changing the doxorubicin cellular accumulation [44].

**Fig. 14:**
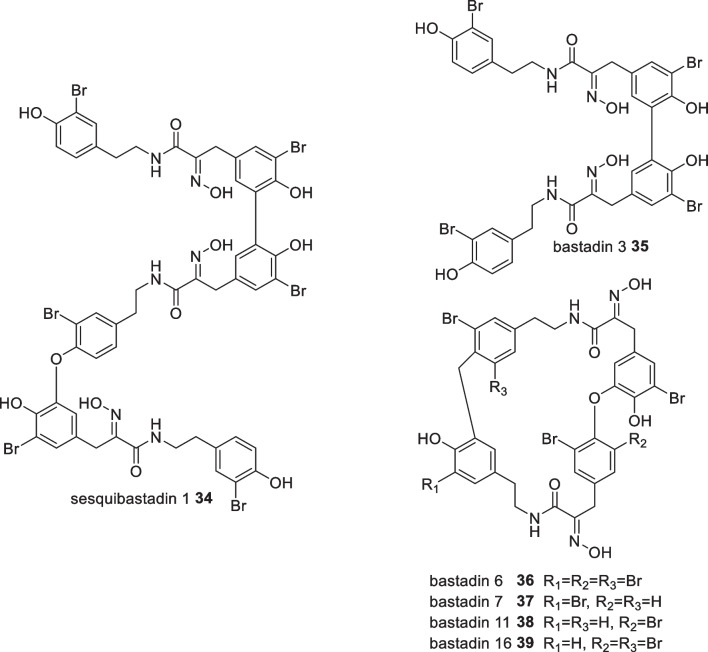
Bastadins isolated from Indonesian *Ianthella basta*

Secondary metabolites isolated from the Indonesian sponge *Ianthella basta*, collected from Manado Bay Sulawesi, included unique peptide derivatives, mostly in cyclic form, namely the bastadins (**34**–**39**).[45–47] In 2012, the Proksch group studied the same species collected from the Ambon Ocean Moluccas and successfully isolated the hemibastadin congener, sesquibastadin 1 **34** along with bastadins 3 **35**, 6 **36**, 7 **37**, 11 **38** and 16 **39** which showed inhibitory activity against several kinase proteins (Fig. 14). Compared to the cyclic bastadins, the linear bastadin congeners, sesquibastadin 1 **34** and bastadin 3 **35** were most active against at least 22 protein kinases, with IC_50_ values ranging from 0.1 to 6.5 μM [48]. A SAR study using synthesised analogues of bastadin 6 **36** in which modification of the oxime moiety and bromine substituent exposed their importance in antiproliferative activity performed under human umbilical vein endothelial experiment [49].

**Fig. 15:**
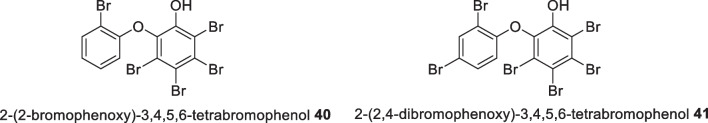
Bromophenols isolated from Indonesian *Dysidea herbacea*

2-(2-Bromophenoxy)-3,4,5,6-tetrabromophenol **40** and 2-(2,4-dibromophenoxy)-3,4,5,6-tetrabromophenol **41**, isolated from a *Dysidea herbacea* sample collected in West Sumatra along with four other compounds were reported to have the most cytotoxic activity amongst all of the isolated compounds in the brine shrimp lethality test with LC_50_ values of 0.96 and 0.94 µg/mL, respectively (Fig. 15). The structure–activity relationship of different polybrominated diphenyl ether derivatives suggested that the strong cytotoxic activity of these two compounds was due to the higher number of bromine substituents in both of the compounds [50].

**Fig. 16:**
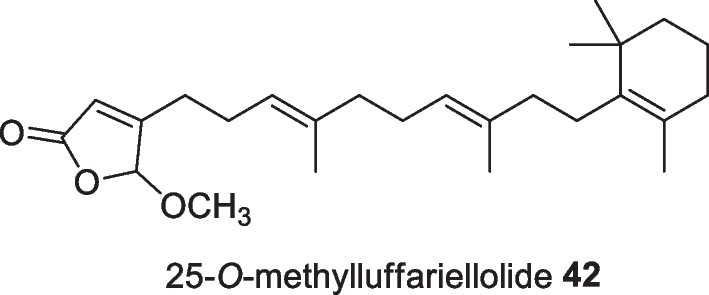
Sesterterpene isolated from Indonesian *Acanthodendrilla* sp.

The sesterterpene 25-*O*-methylluffariellolide **42** (Fig. 16) is a derivative of luffariellolide and was isolated as a yellowish oily residue from *Acanthodendrilla* sp. which was collected at a depth of 16–20 ft near the coast of Kundingarengkeke Island, Indonesia. This compound showed significant cytotoxic activity against mouse lymphoma (L5178Y cells) with an IC_50_ value of 0.7 µg/mL, whereas its ethoxy congener was found to be inactive. The cytotoxic activity against L5178Y cell lines is sensitive to the stereochemistry of 1-cyclopentan-5-ol [51].

**Fig. 17:**
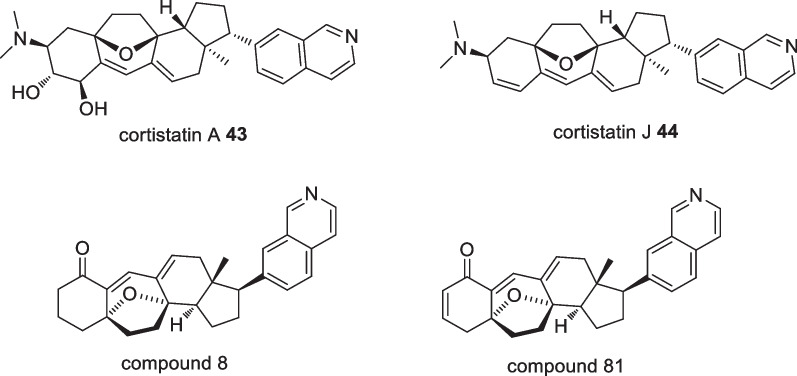
Sesterterpenes isolated from Indonesian *Acanthodendrilla *sp.

Bioassay-guided investigation of *Corticium simplex* collected from Flores island produced eleven new steroidal alkaloids, including cortistatins A **43** and J **44**, two potent antiproliferative agents exhibiting strong activity against HUVECs with IC_50_ values of 1.8 and 8 nM, respectively (Fig. 17). Further studies were conducted to evaluate the effect of cortistatin A **43** on the migration and tubular formation as well as protein phosphorylation in HUVECs in which cortistatin A **43** was reported to inhibit VEGF-induced migration and bFGF-induced tubular formation. However, the compound did not show inhibition on the phosphorylation of ERK1/2 and p38 [52]. A total synthesis of cortistatin A **43** and J **44** was succesfully attempted resulting in synthetic cortistatins A **43** and J **44** with identical physical properties with the natural substances. Two analogues were also synthesised coded as analogues 8 and 81, both missing the dimethylamino and hydroxyl groups of cortistatin A **43**. In addition, the synthetic cortistatins A **43** and J **44** along with two other selected analogues were evaluated for their antiproliferative effects against several cell lines including HUVECs in which the results reported all of the synthetic compounds and the selected analogues showed comparable antiproliferative activities against HUVECs with the natural substance [53].

**Fig. 18:**
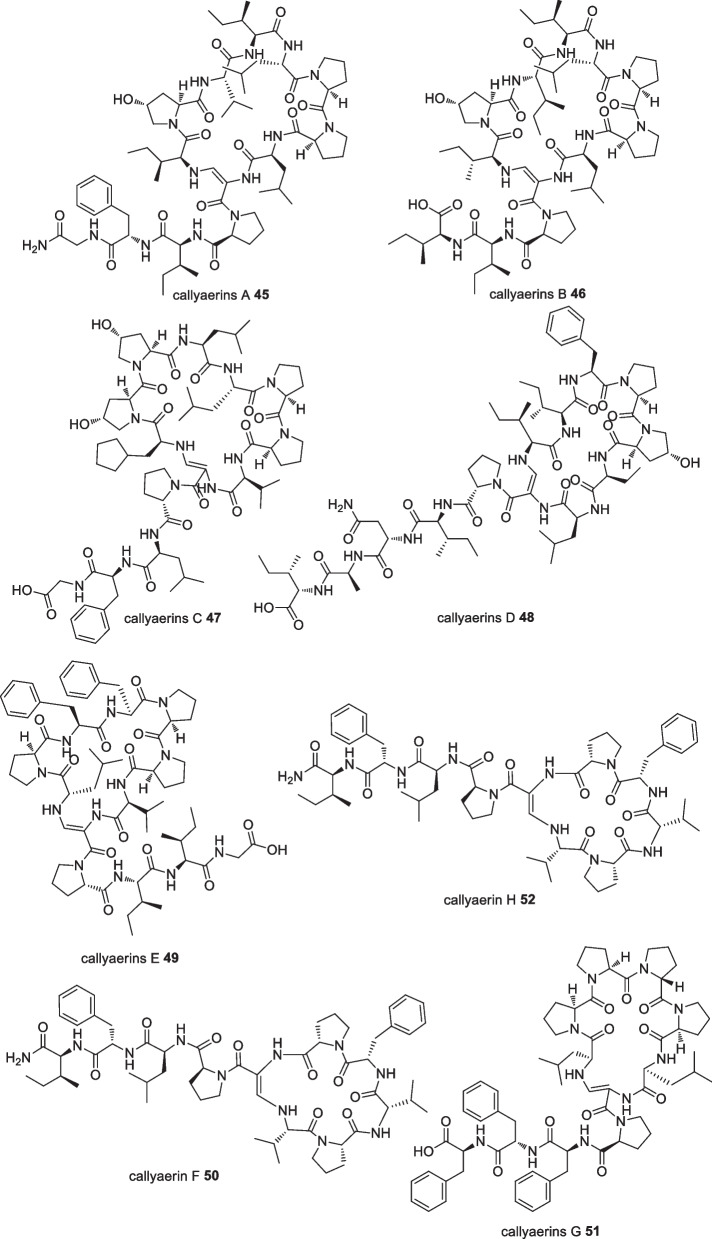
Cyclic peptides isolated from Indonesian *Callyspongia aerizusa*

In 2008, callyaerin G **51**, a new cyclic peptide, was isolated from the ethyl acetate fraction of the Indonesian sponge *Callyspongia aerizusa* from Ambon. Anticancer evaluation indicated the compound had potent cytotoxic activity against the mouse lymphoma cell line (L5178Y) with an ED_50_ value of 0.53 µg/mL [54]. Further investigation through bioassay guided fractionation of *C. aerizusa* in 2010 yielded seven novel cyclic peptides; callyaerins A-F (**45–50**) and H **52** (Fig. 18). The compounds were evaluated for their cytotoxicity against several cell lines in which only Callyaerins E **49** and H **52** showed strong cytotoxicity against the L5178Y tumour cell line with ED_50_ values of 0.39 and 0.48 µM, respectively. It was suggested that the cytotoxicity of the callyaerins was due to the number of proline residues in the cyclic moiety [55], however no further cytotoxic studies on this proline congener were reported.

**Fig. 19:**
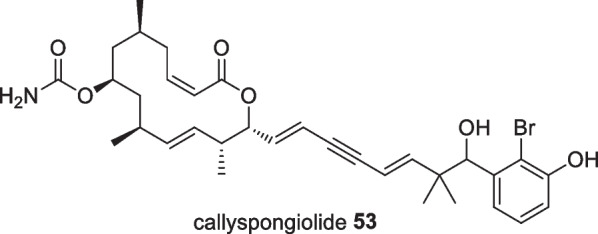
Anti-cancer macrolide from Indonesian *Callyspongia* sp.

A novel cytotoxic polyketide-derived macrolide, callyspongiolide **53**, was isolated from the Indonesian marine sponge *Callyspongia* sp. as a light yellowish amorphous solid in which the compound exhibited significant cytotoxicity against L5178Y mouse lymphoma cells, human Jurkat J16T and Ramos B lymphocytes, with IC_50_ values of 320, 70 and 60 nM respectively (Fig. 19). The cytotoxicity on L5178Y cells was approximately 13 times more active than kahalalide F as the positive control (IC_50_ 4300 nM) [56]. This macrolide promoted autophagy-dependent cell death arising by mitochondrial damage through iron depletion by lysosomal deacidification [57].

**Fig. 20:**
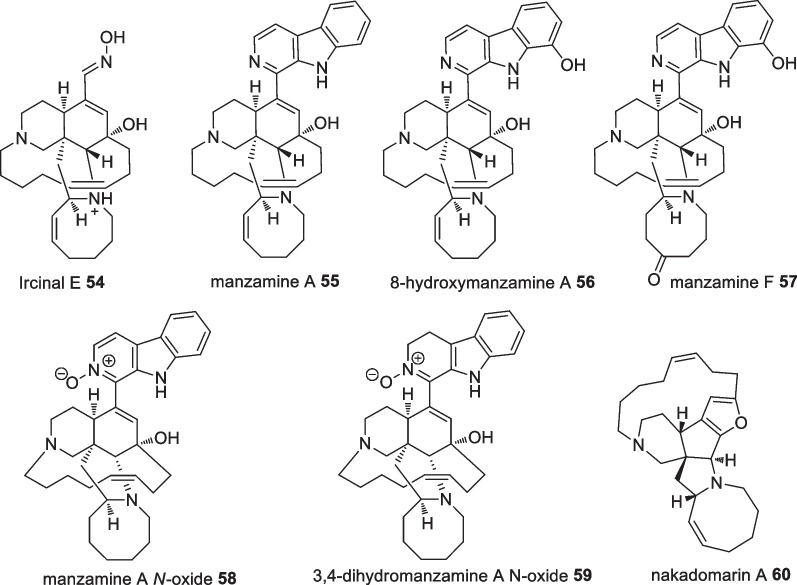
Cytotoxic active manzamine from Indonesian *Acanthostrongylophora ingens*

The investigation of the Indonesian sponge *Acanthostrongylophora ingens*, collected at a depth of 10 m in Ambon, led to the isolation of a new manzamine derivative ircinal E **54** along with six identified compounds, manzamine A **55**, 8-hydroxymanzamine A **56**, manzamine F **57**, manzamine A *N*-oxide **58**, 3,4-dihydromanzamine A *N*-oxide **59**, and nakadomarin A **60** (Fig. 20). Cytotoxic evaluation of the isolated compounds on L5178Y cells showed 3,4-dihydromanzamine A *N*-oxide **59** possessed the highest cytotoxicity with an IC_50_ value of 2.8 µM which was superior compared to kahalalide F (~ 1.5-fold, IC_50_ 4.3 µM). The new compound, ricinal E **54**, showed only weak cytotoxicity against the tested cell line [58].

#### Anti-infective agents

**Fig. 21:**
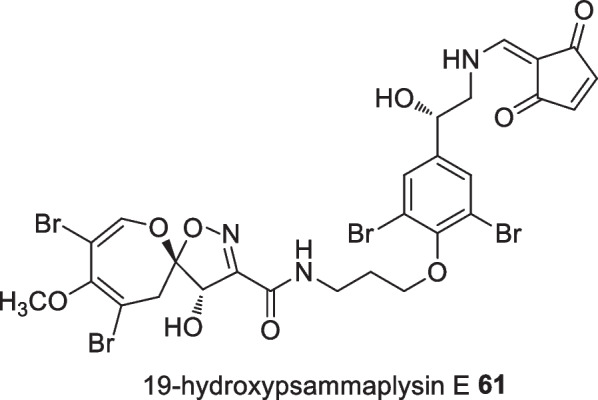
The antimalarial active psammplysisn derivative **61** isolated from Indonesian sponge *Aplysinella strongylata*

The interest in Indonesian sponges led to an investigation of Balinese marine sponges by Garson et al, in which twenty one new psammplysisn derivatives were successfully isolated from *Aplysinella strongylata* with 19-hydroxypsammaplysin E **61** possessing the highest activity against chloroquine-sensitive *Plasmodium falciparum* 3D7 strain with an IC_50_ value of 6.4 µM (Fig. 21) [59].

**Fig. 22:**
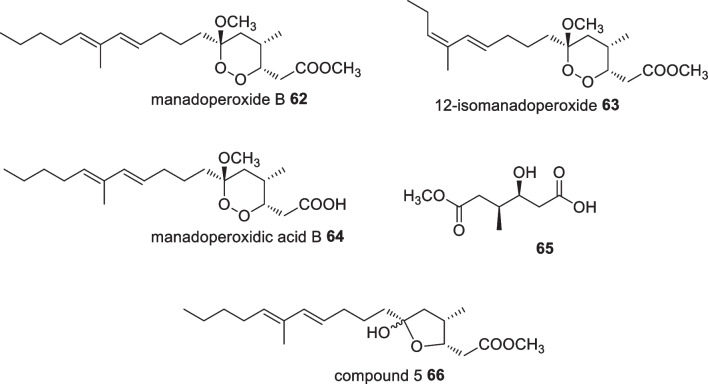
Sponge peroxides isolated from Indonesian *Plakortis cfr. Simplex*

Investigations on the Indonesian Plakortis species successfully isolated cyclic peroxides, manadoperoxides A-D from *Plakortis cfr. Simplex* collected from the coast around the Bunaken Islands [60]. An antiplasmodial bioassay indicated moderate activity against both the D10 and W2 strains of *P. falciparum.* Further examination on this genus revealed manadoperoxide B **62**, 12-isomanadoperoxide B **63**, manadoperoxidic acid B **64** and decarboxylate mono ester **65** from *P. cfr. Lita* collected from the Bunaken Marine Park (Fig. 22) [61]. These peroxides were evaluated for their trypanocidal activity in which among the peroxides, manadoperoxide B **62** possessed high potency with an IC_50_ value of 8.8 nM [61]. Reductive cleavage on manadoperoxide B **62** removed the peroxide moiety to produce tetrahydrofuran **66**, which resulted in a significant decrease in the trypanosomal activity showing no activity at 20 ug/mL.

**Fig. 23:**
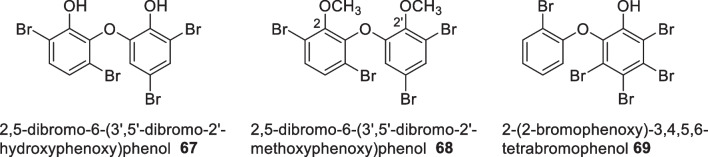
Bioactive polybrominated diphenyl ethers sourced from *Lamellodysedia herbacea* and *Cladosporium cucumerinum*

Fourteen polybrominated diphenyl ethers were isolated from the Indonesian sponge *Lamellodysedia herbacea* collected from Sangiang Island, some of which were further derivatised through methylation and debromination to produce a total of 22 analogues [62]. Overall, the study revealed methylation of 2,5-dibromo-6-(3′,5′ -dibromo-2′ -hydroxyphenoxy)phenol **67** at the C2 and C2′ position yielded **68** which eliminated antibacterial activity against *Bacillus subtilis* (Fig. 23) [62]. Phytochemical investigations on the Indonesian sponge *Dysidea herbacea* isolated four new polybrominated diphenyl ethers congeners and three known derivatives in which 2-(2-bromophenoxy)-3,4,5,6-tetrabromophenol **69** showed the highest antibacterial activity against *Bacillus subtilis* with a MIC value of 0.20 µg/mL. All isolated compounds were found to have fungicidal activity against *Cladosporium cucumerinum* with inhibition zones ranging from 1.5 to 8.0 mm at a concentration of 25 nmol (Fig. 23) [50].

**Fig. 24:**
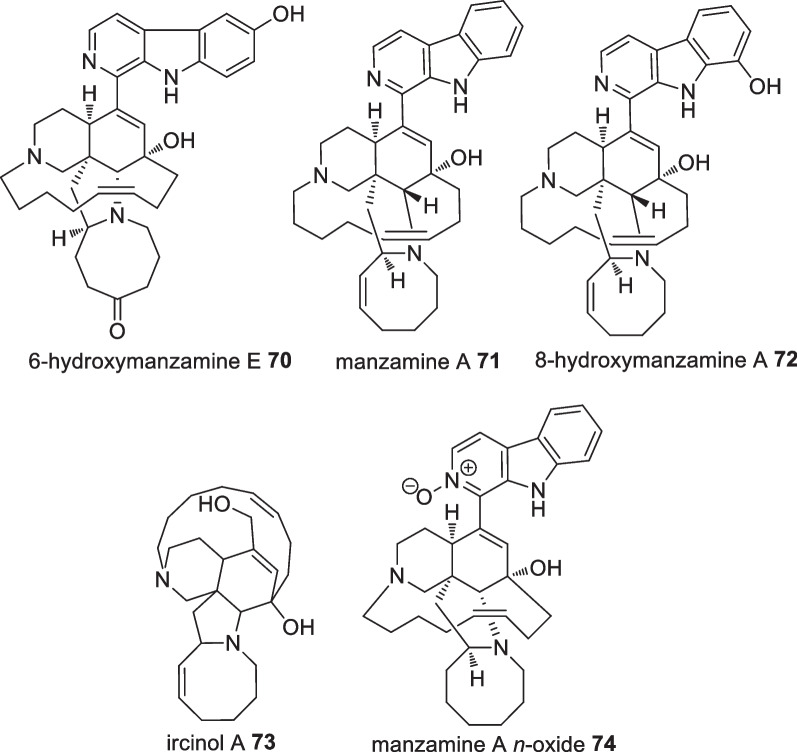
Bioactive manzamine compounds isolated from Indonesian *Achantostrongylophora* sp

Three new manzamine alkaloids were isolated from the Indonesian sponge, *Achantostrongylophora* sp., along with 12 known manzamine alkaloids. The isolated compounds exhibited significant biological activities against *Mycobacterium tuberculosis* (H37Rv), *P. falciparum*, and *Leishmania donovani* (Fig. 24). 6-Hydroxymanzamine E **70** showed the highest antibacterial activity against *M. tuberculosis* with a MIC value of 0.4 µg/mL which was comparable to the positive control rifampin. The antimalarial activity was tested on *P. falciparum* in which manzamine A **71**, (+)-8-hydroxymanzamine A **72**, and manzamine A *N*-oxide **74** displayed remarkable activities with IC_50_ values of 4.5, 6.0, and 11 ng/mL, respectively against the D6 clone *P. falciparum* and 8.0, 8.0, 13 ng/mL, respectively against the chlorine resistant W2 clone *P. falciparum*. These activities were reported better than the positive control, chloroquine, and more than tenfold greater when tested against chlorine resistant W2 clone *P. falciparum*. It was suggested that the hydroxyl group and its arrangement on the *β*-moiety might be responsible for the biological activities of manzamine-type alkaloids. Ircinol A **73** and manzamine A **71** exhibited significant leishmaniacidal activities with an IC_50_ value of 0.9 µg/mL. The isolated compounds were also evaluated for their anti-HIV-1 activity with only (+)-8-hydroxymanzamine A **72** showing an EC_50_ value of 0.59 µM [63].

### Ascidian

Ascidians consist of more than 4000 species, mostly benthic, solitary or in colonies in marine habitats, associated with mostly intertidal ecosystems (rocky, sandy, coral reefs, sea grass, and mangrove areas). They are widely distributed from the Atlantic to tropical areas, and survive in a wide variety of environmental conditions, such as high-current fields on rocks; a few of them are invasive species [64, 65]. In the field, the shape of adult benthic ascidians are often difficult to distinguish from sponge colonies. In order to survive they store calcareous spicules along the body and mantle as a physical defense mechanism. Another survival technique uses secondary metabolites for the chemical defense against predators. Therefore, compounds produced by ascidians are of great interest, e.g. as potential drug leads, as biofuels, and supplementary feed such as omega 3 fatty acid, thus providing value in bioprospecting [64].

**Fig. 25:**
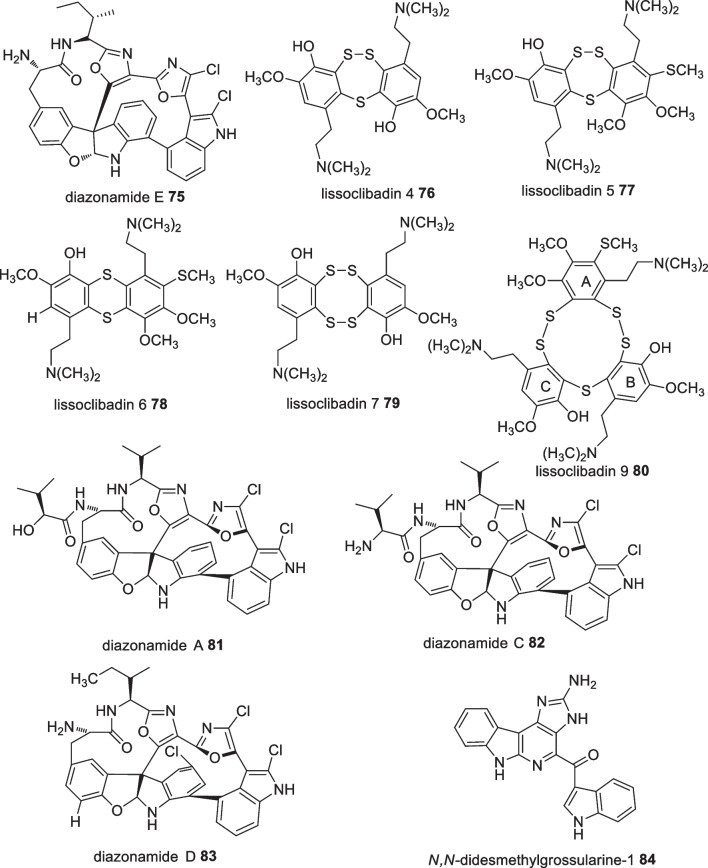
Bioactive compounds sourced from Indonesian Ascidians

A number of Indonesian marine species were identified which possess potential pharmacological activity (Additional file 1: Table S2). Fifty-two bioactive molecules isolated from seven species in the Ascidian family were tested against various cancer cell lines. A separation protocol of *Diazona* sp. produced four compounds including diazonamides A **81**, C **82**, D **83**, and E **75** (Fig. 25). Diazonamide E **75** displayed the highest cytotoxic activity against A549, HT29, and MDA-MB-23 cell lines with GI_50_ values of 0.029, 0.006, and 0.07 µM, respectively [66]. However, diazonamides A **81**, C **82**, and D **83** only showed moderate anticancer activity against A549 (GI_50_ 1.8–2.2 µM), HT29 (GI_50_ 2.9–3.1 µM), and MDA-MB-231 (GI_50_ 5.2–9 µM) [66]. Furthermore, lissoclibadins 4, 5, 6 and 7 (**76–79**) (Fig. 25) were isolated from *Lissoclinum cf. badium* and were reported to inhibit the colony formation of Chinese hamster V79 cells with EC_50_ values of 0.71, 0.06, 0.06, and 0.17 µM, respectively [67]. Similarly, lissoclibadin 8 was reported to have a remarkable antiproliferative effect against colony formation of Chinese hamster V79 cells with an IC_50_ value of 0.14 µM, while lissoclibadin 9 **80** showed highly cytotoxic activity against L1210 murine leukemia cells with an IC_50_ value of 0.38 µM [68]. In addition, studies on *Polycarpa aurata* revealed that *N,N-*didesmethylgrossularine-1 (DDMG-1) **84** displayed inhibition against TNF-*α* production in lipopolysaccharide-stimulated murine macrophage-like RAW265.7 cells, indicating a potential ability in tumor elimination as TNF-*α* was widely associated in the growth, invasion, and cancer metastasis [69].

### Gorgonians

**Fig. 26:**
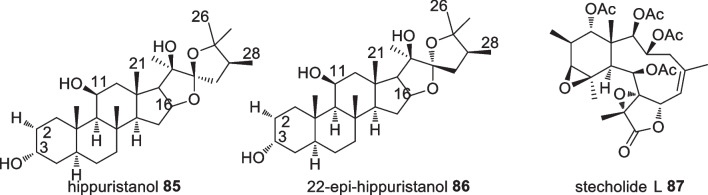
Bioactive compounds sourced from Indonesian gorgonians

Gorgonians, or octocoral, live in currents primarily to optimise food intake. Three species in the gorgonian family (Additional file 1: Table S3) were reported to produce thirty-five isolates. Among them, three compounds were reported to produce potential anticancer activity against several cell lines. For instance, stecholide L **87** sourced from *Briareum* sp. showed remarkable cytotoxic activity against P-388, A549, and HT-29 cell lines with IC_50_ values of 10, 2.5, and 5 µM, respectively [70]. Additionally, the steroidal compounds hippuristanol **85** and 22-epi-hippuristanol **86** (Fig. 26) displayed high cytotoxic activity against P-388, A549, HT49, and MEL28 cell lines with an IC_50_ value of 0.1 µg/mL for each [71]. Hippuristanol **85** and 22-epi-hippuristanol **86** were the most active isolates in which steroids bearing a spiroketal ring are responsible for this feature. Hippuristanol **85** was shown to influence mRNA translation as it actively interferes with the eukaryotic initiation factor 4A (eIF4A) resulting in potent anti-tumorigenic activity [72]. A combinatorial chemistry approach synthesised several analogues and it was found that elevating the oxidation state of the hydroxyl groups depleted its activity, as did removal of the methyl substituent [72].

### Algae

Indonesia is a tropical nation and is situated within the coral triangle. It is renowned for its abundance of marine species including the large taxonomic group of algae due to its species diversity among the marine organisms of Indonesia. According to the "Sibolga Expedition", there are 555 species of algae living in Indonesia [73]. In addition, Atmadja and Prud’homme van Rein [74] documented the presence of 201 species of Chlorophyta (Ulvophyceae/green algae) belonging to 7 orders, 24 families, and 48 genera; and 138 species of Ochrophyta (Phaeophyceae/brown algae) belonging to 6 orders, 12 families, and 31 genera. Meanwhile, the total number of Rhodophyta (Florideophyceae and Bangiophyceae/red algae) is 452 species.

In addition to their biodiversity, marine algae, including macro- and microalgae, are potential industrial commodities as marine biological resources. Indonesia is one of the five largest producers of macroalgae in the world and plays a role in the development of marine algae-based industries as a supplier of biomass raw materials. Six species are harvested commercially in Indonesia with four (*Eucheuma spinosum*, *Kappaphycus alvarezii*—also known commercially as *Eucheuma cottonii*, *Gracilaria* sp., and *Caulerpa* sp.) being cultivated while the other two (*Gelidium* spp. and *Sargassum* spp.) are primarily collected from natural populations [75]. The largest seaweed production based on wet weight (tonnes) comes from the Sulawesi (50.63% of total production) followed by the West and East Nusa Tenggara province with 31.85% of total production [76]. The islands of Java, Bali, Kalimantan, Moluca and Papua contribute 17% of total production.

Algae have been utilized in the production of food, pharmaceuticals, health-related products, nutraceuticals, cosmetics, fine chemicals, feed components, feed additives, aquaculture products, and agricultural products [77]. Marine macroalgae or seaweeds have been consumed as food, particularly in China and Japan, and as traditional drugs for the treatment of a variety of illnesses, including iodine deficiency. Bioactive seaweed substances are a group of chemical components extracted from seaweed biomass that can influence the biological processes of living organisms via chemical, physical, and other mechanisms [78]. Indonesian macroalgae have been historically significant sources of natural products with applications in cosmetics, nutraceuticals, and pharmaceuticals, particularly as the source of bioactive compounds with antibacterial, anticancer, anti-inflammatory, antioxidant, etc. activities [79–85].

The bioprospecting of Indonesian algae is described in Additional file 1: Table S4 and covers species, location, constituents and biological activities. There was a major research focus on the medicinal potency of crude extracts with only a few chemical isolations reported. Pharmacological evaluation indicated significant activity, however required large doses which is disadvantagous and limited further investigation. Testing of the crude protein extract of erect cactus (*Halimeda macrobola*) collected from the Selayar and Kapoposang Islands was significantly lethal to Hela cell lines with a LC_50_ value of 0.29 µg/mL, although the species remain understudied [86]. The less polar components of *Gymnogongrus* sp indicated a strong antibacterial activity against *Escherichia coli,* and *Salmonella thypi* with respective inhibition zone diameter of 42.33 and 37.50 mm; despite no further exploration reported from this species [87]. The polar components of brown algae, *Padina* sp., showed strong antibacterial activity against *E. coli* with inhibition zone value of 26.5 mm [88]. A bioguided protocol for antibacterial agent discovery was applied to *Gracilaria edulis*, in which gradient fractionation of the less polar component revealed ethyl acetate hexane (1:1) fraction possessed comparable antibacterial against *Vibrio fluvialis* and *Aeromonas hydrophyla* with MIC values of 1.25 and 0.625 ug/mL, respectively. Analysis of the volatile component detected hexadecanoic acid which is a bacterial pathogen providing protection to fish and is therefore promising for the aquaculture industries [89].

### Mangroves

Mangroves, as coastal vegetation adapted to thrive in brackish environments, are intricately linked to the marine ecosystem, occupying the intertidal zone between land and the ocean. They are essential elements in coastal and estuary ecosystems in the tropics and subtropics, protecting the ecosystems from various natural disasters [90] while also serving as habitats for other species and providing abundant sources of food and natural medicines [90–92]. Indonesia's coastal zones are renowned for hosting the world's largest mangrove areas, which exhibit exceptional biodiversity [93–95], with forty-five species out of the seventy-five true mangroves found in Indonesia [94]. Most of these species have been utilized by Indonesian communities to treat various diseases, both as preventative and curative measures. However, investigations into the phytochemistry of these plants significantly lags behind the existing knowledge of their indigenous uses. To ensure the validity and reliability of this review on the natural medical aspects of Indonesian mangroves, we have specifically included the most authoritative and relevant existing publications published since the 2010s. These publications comprise studies conducted by local research institutions, universities in Indonesia, and collaborating international research institutions, and have been published in both local and international peer-reviewed journals. Older publications predating the 2010s were thoroughly evaluated and excluded from the review if they lacked comprehensive scientific reporting or presented redundant information compared to more recent publications.

Medically active metabolites from mangrove plants can also be produced by related microbes such as endophytic fungi, instead of being synthesized by the plants themselves [96–98]. Consequently, microorganisms like mangrove endophytes or soil-derived mangrove fungi and bacteria play a significant role in the production of natural compounds, their derivatives, and their medicinal activities. However, delving into these topics would require extensive reporting and discussion, which goes beyond the scope of this review.

**Fig. 27:**
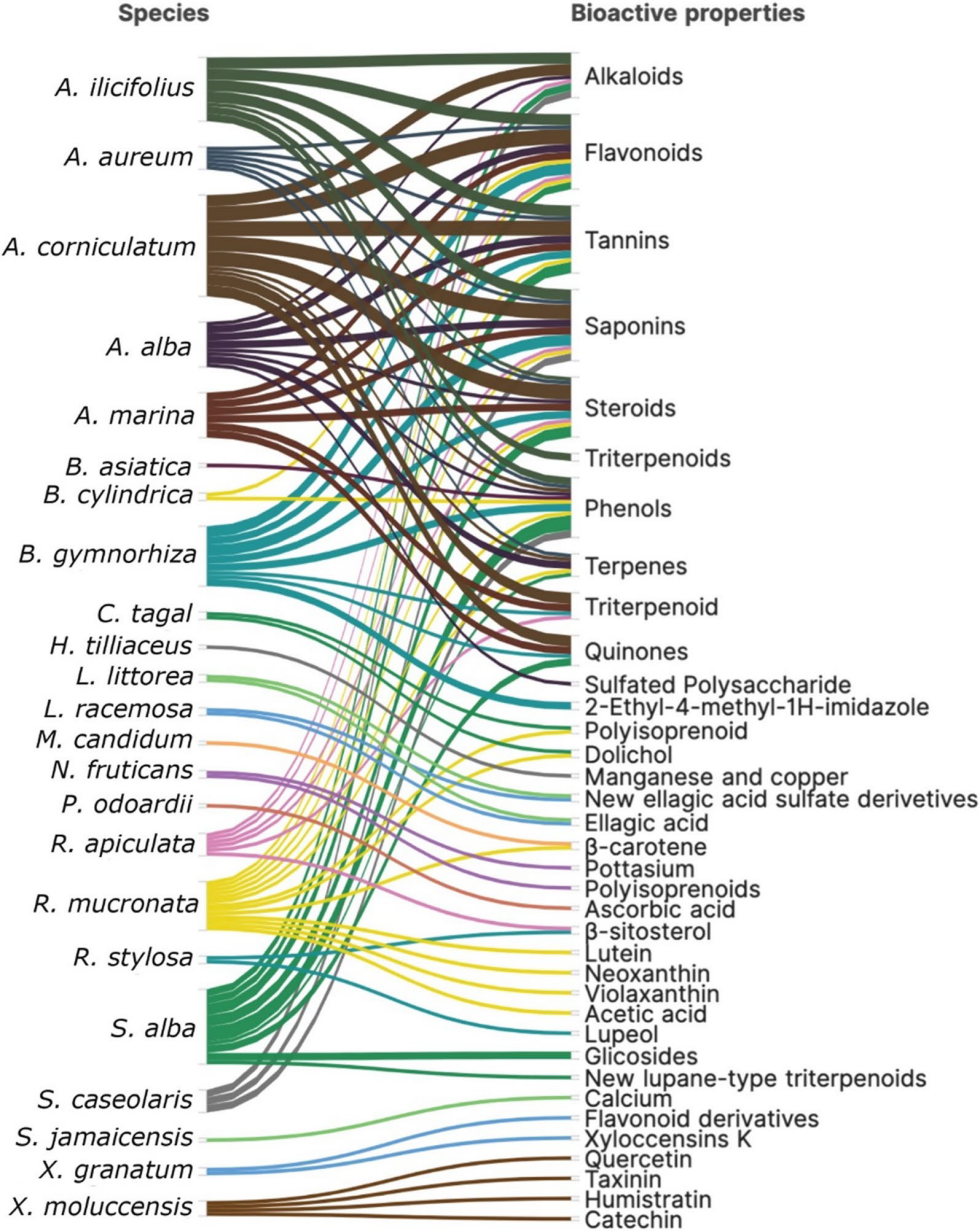
Phytochemical properties of mangrove species from Indonesia

Inclusive of the ecological function in marine ecosystems, mangrove plants have been widely used in medicine by local communities in many parts of the world due to their bioactive natural products. In Indonesia, over the last decade, a considerable number of 35 research studies have been published, primarily focused on 23 species out of the 45 mangrove species found in the country. These studies have specifically examined and reported on thirty-six major bioactive properties (see Additional file 1: Table S5). These compounds have been isolated from various tissues of mangroves including leaves, roots, bark, stem, fruits, and flowers. The most common compounds that occur at the family and species levels are flavonoids, saponins, tannins, phenols, triterpenoids/steroids, alkaloids, quinones, terpenes/triterpenoids (Fig. 27).

The production of secondary metabolites in various tissues of mangroves is prone to both developmental biology and environmental factors. Occurring in harsh environmental conditions, mangrove plants require specific development mechanisms including morphology and physiological adaptations. These adaptations are vital for their growth in intertidal zones where mangroves must cope with challenging ecological conditions such as high salinity, seawater inundation, and anoxic soil, as well as heat stress and cold stress. Therefore, in order to survive, the production of secondary metabolites play important roles. Moreover, particular metabolites may be produced as a response to specific stress adaptations. For instance, terpenes in mangroves have been reported to be crucial for adaptation to salt stress caused by sea water [99], while flavonoids are associated with heat response [100]. Additionally, metabolites derived from mangroves have demonstrated potential for pharmacological application (see Additional file 1: Table S5).

The major bioactive compounds identified for their pharmacological potential include antioxidants, anticancer, antibacterial, antimicrobials, anti-infectives, antiplasmodial, anthelmintics, wound treatment, anti-inflammatory, antimalarial, analgesics, anti-hyperuricemia, and anticoagulants (Additional file 1: Table S5). The most investigated species were *Bruguiera gymnorhiza* (L.) Lam., *Rhizophora mucronata* Lam., *Sonneratia alba* Sm., *Acanthus ilicifolius* L. Importantly, some bioactivities were sufficient high, leading to the further development as anticancer agents [101–105], antioxidants [106–110], antihyperuricemics [111], and anti-infectives [112]. However, numerous species in various locations known from traditional medicine remain to be explored, e.g. the local community in North Maluku has been using *Lumnitzera littorea*, *R. mucronata*, *Scyphiphora hydrophyllacea, S. alba*, *Xylocarpus granatum*, and *Xylocarpus moluccensis* to treat malaria [113]. This highlights the significant potential for further research and exploration of mangroves as sources of medicinal leads.

In addition to researching the remaining mangrove species, exploring the species across the Indonesian Archipelago shows promising prospects. So far, most of the sampling efforts in investigating mangroves have been focused on Java and Sumatra Islands, with limited representation from the other regions such as Sulawesi, Papua and Nusa Tenggara Timur (Additional file 1: Table S5). Recently, a study [112] reported that pharmacological activities in two mangrove species, *L. littorea* and *L. racemosa,* surprisingly corresponded to their local distributions across the archipelago. Specifically, the study identified strong antibacterial activities in only in the root extracts of *Lumnitzera littorea* from Southern Nias Island and East Java, while the root extracts of *L. racemosa* from Ternate Island and East Java exhibited even stronger antibacterial activities compared to other samples across the regions.

### Indonesian marine microorganisms

#### Bioactive compounds from Indonesian marine fungi

Fungi isolated from Indonesian marine habitats have been explored since the 1970s. The marine fungi showed various biological activities, including antibacterial [114–118], antifungal [117, 119], anticancer [117, 120–122], anti-obesity [123], enzyme producer [118, 124–126], and immunostimulant [127]. Marine fungi of Indonesian origin were isolated from several biota as the fungal host, mostly sponges, seaweeds and mangroves. In this review, we present the updates on the marine fungal isolation, characterization and related studies of fungi from Indonesian waters. Marine fungi are fungi isolated from marine habitats, either as obligate marine fungi which grow and sporulate exclusively in a marine or estuarine habitat or facultative marine fungi which are fungi from freshwater or terrestrial milieus able to grow and probably sporulate in marine environment [128]. Due to their productivity in producing bioactive compounds, marine fungi have been explored more intensively.

In the marine environment, fungi are associated with other organisms or substrates. Sponge-associated fungi were dominantly isolated, followed by algicolous and mangliciolous fungi, those isolated from marine algae and mangrove, respectively. Sponges are the most productive marine biota producing bioactive compounds. Sponges use fungal secondary metabolites as a chemical defence against predation and survival in toxic or extreme environmental conditions [129]. Marine sponges contain diverse and abundant microbial communities, including bacteria, archaea, microalgae and fungi [130], with the microbial associates comprising about 40% of the sponge’s volume and contribute significantly to host metabolism. Venkataraman et al. (2022) report that sponge associated fungi are a promising source of pharmacologically active compounds with anticancer, antibacterial and antiviral activities [131].

Marine fungi from Indonesian waters have been underexplored compared to marine bacteria or macro-organisms, such as sponges, corals, seaweeds, and mangroves. In 2021–2022 only 16 articles were published with 53% of the fungal samples isolated from sponges, about 27% from seaweeds and 20% from mangroves. Sponges as fungal hosts comprised *Monanchora* sp. [132], *Ancorina* sp. [121], *Clathria* sp. [119], *Petrosia* sp., *Stylissa carteri, Cinachyrella australiensis, Callyspongia* sp., *Petrosia nigrians, Stylissa massa *[133], *Stylissa flabelliformis* [122], and *Stylissa* sp. [123]. Some species of the sponges were not identified. Seaweeds as biological sources of the marine fungi were identified as *Padina* sp., *Asparagopsis* sp., and *Chondrophycus* sp. [134], green alga *Bornetella* sp. [116] and unidentified brown algae [125]. Species of mangroves included *Avicennia marina*, *Aegiceras corniculatum* [135], *Avicennia* sp., *Sonneratia* sp., *Rhizophora* sp. [118], *Rhizophora apiculate* [126]. Identification of fungi is mostly conducted morphologically, though, in the case of marine fungi, it is difficult to produce generative spores in lab culture. Therefore, molecular identification plays a key role. *Aspergillus* and *Penicillium* are still the most productive species in producing bioactive compounds, as well the most well-studied. Species of marine fungi reported in 2021–2022 were *Aspergillus* spp. [115, 133], *Aspergillus flavus* [122]*, Aspergillus fumigatus* [122], *Aspergillus ochraceus, Aspergillus niger*, *Aspergillus nomius* [116], *Aspergillus aculeatus* [118], *Penicillium* spp. [122, 126], *Penicillium citrinum* [118], *Penicillium oligosporum* [125], *Diaporthe stewartii* [118], *Diaporthe* spp.[126], *Fusarium equiseti* [118], *Trichoderma reesei* [118, 132], *Trichoderma viride* [118], *Colletothrichum* spp. [126], *Hypocrea* sp., *Pestalotiopsis theae, P. microspora* [118], *Gymnoascus udagawae* [119], *Microdochium* sp. [135], and *Purpureocillium lilacinum* [123]*.*

From those reported marine fungi, sample collecting was still concentrated in the surroundings of Java Island (56%), followed by Sulawesi Island (19%), Sumatera Island (13%), and Bali (6%). Additional file 1: Table S6 presents the origin of the marine fungal isolates in more detail. Overall, the studies reveal moderate activities against several bacteria, however impure extracts were used.

#### Bioactive compounds from Indonesian marine bacteria

Marine microorganisms are possibly the next bio-manufacturer of diverse bioactive compounds in addition to terrestrial plants and nonmarine microorganisms [14, 136]. Other than eight clinically approved marine-derived natural products on the market [13], more than twenty more marine natural products and derivatives have advanced to clinical trials, including plinabulin, lurbinectedin, tetrodotoxin, salinosporamide A, and depatuxizumab mafodotin [136]. The biodiversity of marine bioactive products has been driven by the response of marine organisms to several environmental factors, including defense strategies, communication factors, response to the availability of food, and response to physical factors (e.g. temperature, levels of salinity, and hydrostatic pressures) [13, 137].

Until recently, the number of bioactive products isolated from marine bacteria with diverse biological activities ranging from antibiotic to anticancer has further increased [138]. These compounds have been utilised in the food industry, medicine, agriculture, and cosmetics [139]. In general, bacteria species found in the seawater belong to the genera *Vibrio* sp., *Pseudomonas* sp., *Flavobacterium* sp., *Achromobacter* sp., and *Micrococcus* sp. with the genus *Streptomyces* being the major contributor to novel bioactive molecules to date [137]. Marine bacteria face constant challenges from extreme habitats and unfavourable salinity, temperature, pressure, light, pH, oxygen, and nutrient conditions. Thus, in response to these conditions, bacteria often produce unique secondary metabolites as valuable sources in pharmaceutical and biotechnological industries [140]. Many taxonomically novel species have been identified as potential sources of novel bioactive compounds. In particular, marine bacteria reside in the deep-sea sediments have shown to be an abudance source of secondary metabolites that possess new structure diversity and excellent medicinal potential, including as antimicrobials [141], which has given rise to blue biotechnology [142].

**Fig. 28:**
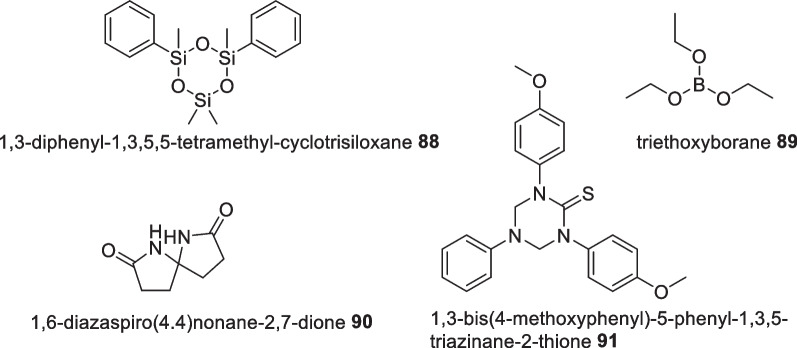
Antivibriosis compounds detected from *Pseudoalteromonas xiamenensis* STKMTI.2

The archipelago of Indonesia is home to a highly broad variety of marine bacteria [12, 14] with examples exhibiting bioactivity tabulated in Additional file 1: Table S7. Compared to micro fungi studies, there are a lesser number of secondary metabolites extracted from Indonesian marine bacteria. Overall, the majority of studies revealed no potency which constrains the studies to crude extracts. Nevertheless, Handayani et al discovered the bacteria isolate, *Pseudoalteromonas xiamenensis* STKMTI.2, exerted antibiotic activity against *Vibrio harveyi, V. parahaemolyticus, V. alginolyticus* with a MIC value under 0.78 µg/mL which is comparable to commerical antibiotics (e.g. chloramphenicol, ampicillin, erythromycin, kanamycin, neomycin). Attempted separation produced an impure mixture of 1,3-diphenyl-1,3,5,5-tetramethylcy clotrisiloxane **88**, triethoxyborane **89**, 1,6-diazaspiro(4.4)nonane-2,7-dione **90**, 1,3-bis(4-methoxyphenyl)-5-phenyl-1,3,5-triazinane-2-thione **91** based on GCMS analysis [143] (Fig. 28). Bacterial community study on *Haliclona fascigera* sponge revealed fifteen of twenty six bacterial isolates possess inhibition activity against MRSA in which a *Corynebacterium sp.4* (N1F2) isolate indicated the highest inhibition zone diameter of 15.7 ± 0.76 mm [144].

## Conclusion

This review reports potential therapeutic natural products from Indonesian marine macro- and micro-organisms. The exploration on sponges indicated several anticancer potent secondary metabolites, Laulimalide **1** (terpenoid from *Hyattella* sp), makaluvamine G **15** (tryptamine-tyramine-derived alkaloid from *Histodermella* sp.), (Z)-hymenialdisine **22** (bromopyrrole alkaloid from *Stylissa carteri*), plakorstatin 2 **29** (peroxide isolated from *Plakortis nigra*), sesquibastadin 1 **34** (unique cyclic peptide from *Ianthella* basta), 2-(2,4-dibromophenoxy)-3,4,5,6-tetrabromophenol **41** (bromophenol derivate isolated from *Dysidea herbacea*), 25-*O*-methylluffariellolide **42** (sesquiterpene isolated from *Acanthodendrilla sp*.), cortistatin A **43** (steroidal alkaloid from *Corticium simplex*), Callyaerins E **49** and H **52** (cyclic peptide from *Callyspongia aerizusa*), callyspongiolide **53** (polyketide-derived macrolide from *Callyspongia* sp.). Trypanocidal manadoperoxide B **62** from *Plakortis cfr. Simplex.* Antibacterial and antifungal 2-(2-bromophenoxy)-3,4,5,6-tetrabromophenol **69** from Dysidea herbacea. Anti TB, 6-hydroxymanzamine E **70** from *Achantostrongylophora* sp. Anti plasmodial, manzamine A **71** from *Achantostrongylophora* sp. Notable results also show ascidian bioprospecting with anticancer lissoclibadin isolation from *Lissoclinum cf. badium.* Gorgonian bioprospecting also revealed an anticancer spiroketal compound, hippuristanol **85** isolated from *Briareum* sp. Despite a good indication on early-stage of bioprospecting on Indonesian marine fungi, bacteria and algae, no isolation protocol has been employed leaving the potent component remained in an unresolved mixture. In addition, there are still no single compounds reported from exploration on Indonesian mangrove vegetation which presents avenues for further research. Overall, despite international collaborative research in marine bioprospecting being conducted in Indonesian oceans, there remains large numbers of understudied of both macro and microorganisms. Further isolation protocols with superior techniques in metabolite profiling will assist research to be more efficient, especially on small scale samples.

### Supplementary Information

**Additional file 1: Table S1.** Chemical and pharmacological data of Indonesian sponges. **Table S2.** Chemical and pharmacological data of Indonesian Ascidian. **Table S3.** Chemical and pharmacological data of Indonesian Gorgonian. **Table S4.** Chemical and pharmacological data of Indonesian marine algae. **Table S5.** Medicinal potential of mangrove species from Indonesia. **Table S6.** Medicinal potential of marine micro fungi species from Indonesia. **Table S7.** Bioactive compounds isolated from marine bacteria in Indonesia and their pharmacological potential.

## Data Availability

The data that support the findings of this study were available on request from the corresponding author, upon reasonable request.
