# Supplementary material for: Indonesian marine and its medicinal contribution
Source: Nat Prod Bioprospect. 2023 Oct 16;13(1):38. doi: 10.1007/s13659-023-00403-1 (PMC10579215; doi:10.1007/s13659-023-00403-1)
Supplement: Supplementary file 1 — Additional file 1: Table S1. Chemical and pharmacological data of Indonesian sponges. Table S2. Chemical and pharmacological data of Indonesian Ascidian. Table S3. Chemical and pharmacological data of Indonesian Gorgonian. Table S4. Chemical and pharmacological data of Indonesian marine algae. Table S5. Medicinal potential of mangrove species from Indonesia. Table S6. Medicinal potential of marine micro fungi species from Indonesia. Table S7. Bioactive compounds isolated from marine bacteria in Indonesia and their pharmacological potential. [file 13659_2023_403_MOESM1_ESM.docx]

**Indonesian Marine and its medicinal contribution**

Ari Satia Nugraha^,a,e,j*^, Lilla Nur Firli^a^, Dinar Mutia Rani^a^, Ayunda Hidayatiningsih^a^, Nadya Dini Lestari^a^, Hendris Wongso^b,c^, Kustiariyah Tarman^d^, Ayu Christien Rahaweman^e^, Jeprianto Manurung^f^, Ni Putu Ariantari^g^, Adelfia Papu^h^, Masteria Yunovilsa Putra^i^, Antonius Nugraha Widhi Pratama^a^, Ludger A. Wessjohann^e^, Paul A Keller^j^

^a^Drug Utilisation and Discovery Research Group, Faculty of Pharmacy, Universitas Jember, Jember, Indonesia 68121; lillanurfirli13@gmail.com, 0000-0002-5173-4384 (L.N.F.); dinarmutiarani@gmail.com, 0000-0003-2633-700X (D.M.R.); ayundanurh@gmail.com, 0009-0001-7750-8721 (A.H.); nadyadini22@gmail.com, 0000-0002-1808-6864 (N.D.L.); anton.farmasi@unej.ac.id, 0000-0001-9106-9924 (A.N.W.P.)

^b^Research Center for Radioisotope, Radiopharmaceutical, and Biodosimetry Technology, Research Organization for Nuclear Energy, National Research and Innovation Agency, Puspiptek, Banten 15314, Indonesia; hend042@brin.go.id, 0000-0003-2802-0452 (H.W.)

^c^Research Collaboration Center for Theranostic Radiopharmaceuticals, National Research and Innovation Agency, Jl. Raya Bandung-Sumedang KM 21, Sumedang 45363, Indonesia; hend042@brin.go.id, 0000-0003-2802-0452 (H.W.)

^d^Department of Aquatic Product Technology, Faculty of Fisheries and Marine Sciences and Centre for Coastal and Marine Resources Studies (CCMRS), Bogor Agricultural University, Indonesia; kustiaz@ipb.ac.id, 0001-6542-542X (K.T.)

^e^Leibniz Institute für Pflanzenbiochemie, Weinberg 3, 06120 Halle (Saale), Germany; ayurahaweman@gmail.com, 0000-0003-2181-310X (A.C.R.); Ludger.Wessjohann@ipb-halle.de, 0000-0003-2060-8235 (L.A.W.)

^f^German Centre for Integrative Biodiversity Research (iDiv) Halle‐Jena‐Leipzig, Puschstrasse 4, Leipzig D04103, Germany; jeprianto_m@apps.ipb.ac.id, 0000-0003-3406-0960 (J.M.)

^g^Department of Pharmacy, Faculty of Mathematics and Natural Sciences, Udayana University, Badung 80361, Bali, Indonesia; putu_ariantari@unud.ac.id, 0000-0001-9657-9958 (N.P.A.)

^h^Biology Department, Faculty of Mathematics and Natural Sciences, Sam Ratulangi University, Manado 95115, Indonesia; adelfia.papu@unsrat.ac.id, 0000-0001-7790-9405 (A.P.)

^i^Vaccine and Drug Research Center, National Research and Innovation Agency, Cibinong, Jawa Barat 16911 Indonesia; mast001@brin.go.id, 0000-0002-9879-3293 (M.Y.P.)

^j^School of Chemistry and Molecular Biosciences, Molecular Horizons, University of Wollongong, Wollongong, New South Wales, 2522, Australia; keller@uow.edu.au, 0000-0003-4868-845X (P.A.K.)

Correspondence: arisatia@unej.ac.id (A.S.N.); Telp: +62 331 324 736 (A.S.N.)

**Abstract**

The archipelagic country of Indonesia is populated by the densest marine biodiversity in the world which have created strong global interest and is valued by both Indigenous and European settlements for different purposes. Nearly 1000 chemicals have been extracted and identified. In this review, a systematic data curation was employed to collate bioprospecting related manuscripts providing a comprehensive directory from 1988 to 2022 publications. Findings with significant pharmacological activities are further discussed through a scoping data collection. This review discusses macroorganisms (Sponges, Ascidian, Gorgonians, Algae, Mangrove) and microorganism (Bacteria and Fungi) and highlights significant discoveries, including a potent microtubule stabilizer laulimalide from *Hyattella sp,* a prospective doxorubicin complement papuamine alkaloid from *Neopetrosia cf exigua*, potent antiplasmodial manzamine A from *Acanthostrongylophora ingens*, the highly potent anti trypanosomal manadoperoxide B from *Plakortis cfr.* Simplex, mRNA translation disrupter hippuristanol from *Briareum sp*, and the anti-HIV-1 (+)-8-hydroxymanzamine A isolated from *Achantostrongylophora sp.* Further, some potent antibacterial extracts were also found from a limited biomass of bacteria cultures. Although there are currently no examples of commercial drugs from the Indonesian marine environment, this review reveals the diversity present and with the known understudied biodiversity, provides great promise for future study.


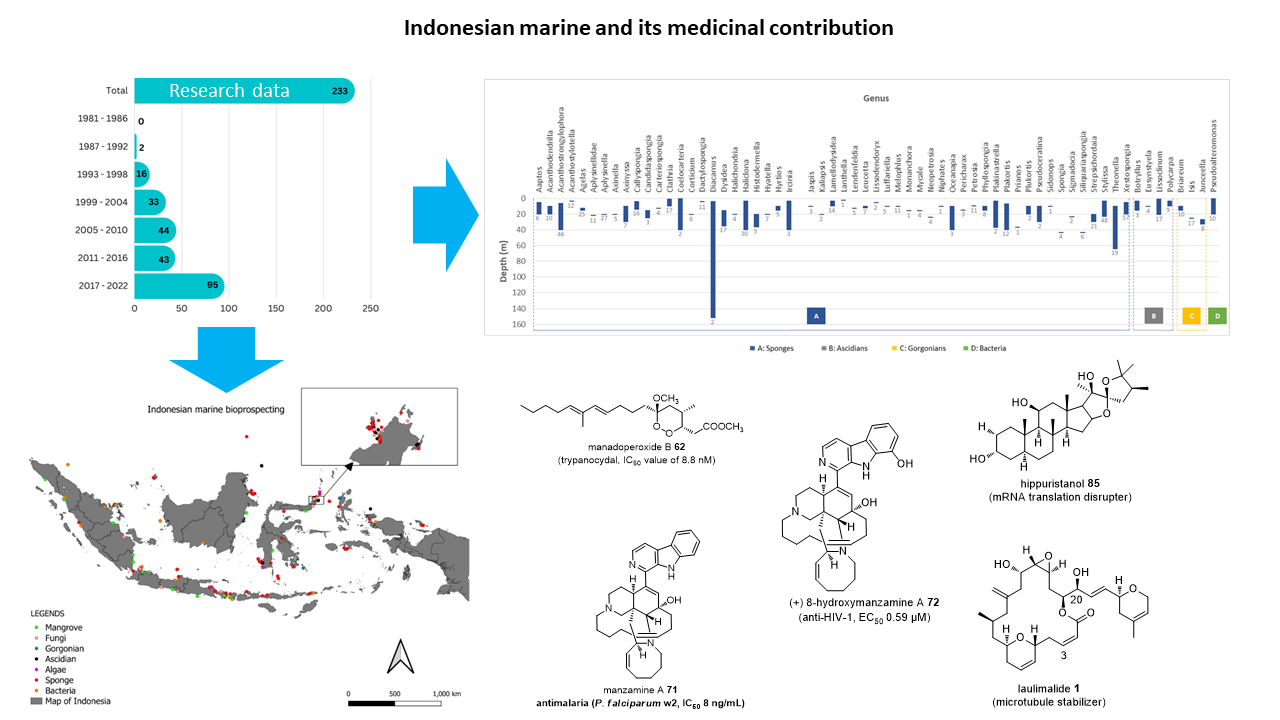


**Keywords**

Indonesian marine; laulimalide; papuamine; manzamine A, manadoperoxide B; hippuristanol; (+)-8-hydroxymanzamine A

**Supplementary Table 1. Chemical and pharmacological data of Indonesian sponges (126 References, 532 Compounds)**

| **No.** | **Type** | **Species** | **Location** | **Constituent** | **Bioactivity** | **Ref.** |
| --- | --- | --- | --- | --- | --- | --- |
| 1 | Sponge | *Aaptos sp.* | Manado and Derawan Island, Indonesia at a depth of 20 m | isoaaptamine;  8,9-demethylaaptamine; aaptamine; 1-*N*-methylaaptamine and  4-*N*-methylaaptamine | All compounds was used in the zebra mussel assay. Isoaaptamine, 8,9-demethylaaptamine, and aaptamine produced EC_50_ values of 24.2, 11.6, and 18.6 µM, respectively while 1-*N*-methylaaptamine and 4-*N*-methylaaptamine were not active.  Isoaaptamine and aaptamine were not phytotoxic to duckweed up to 300 µM (above practical use) whereas 8,9-demethylaaptamine inhibited growth after 7 days of exposure with IC_50_ values of approximately 200 and 20 µM respectively. | [[1](#_ENREF_1)] |
| 2 | Sponge | *Aaptos suberitoides* | North Sulawesi, Indonesia | aaptamine; isoaaptamine and demethylaaptamine | Aaptamine; isoaaptamine and demethylaaptamine inhibited the chymotrypsin-like and caspase-like activities of the proteasome with IC_50_ values of 1.6–4.6 µg/mL. All of the compounds showed less inhibition of the trypsin-like activity of the proteasome with IC50 values of 12–18 µg/mL and were cytotoxic to HeLa cells with IC_50_ values of 15.0, 3.1, and 1.4 µg/mL, respectively. | [[2](#_ENREF_2)] |
|  |  |  | The coastline of Pecaron Bay Situbondo, East Java, Indonesia at the depth of 5-20 m | Crude extract | The specimen showed high toxicity against HT-29, T47D and Casky tumor cell lines with IC50 values of 27.99± 0.98 µg/mL 25.78±1.23 µg/mL, 21.45±1.98 µg/mL, respectively. The specimen also showed strong antioxidant activity due to IC_50_ value of 27.42 µg/mL. | [[3](#_ENREF_3)] |
| 3 | Sponge | *Acanthella sp* | The coastline of Pecaron Bay Situbondo, East Java, Indonesia at the depth of 5-20 m | Crude extract | Cytotoxicity  The specimen moderate antioxidant activity with a IC_50_ < 56.94 µg/mL. The specimen also exhibit high toxicity against HT-29, T47D and Casky tumor cell lines with IC50 values of 37.56±1.21 µg/mL, 482.84±4.56 µg/mL, 37.64±4.21 µg/mL, respectively. | [[3](#_ENREF_3)] |
| 4 | Sponge | *Acanthodendrilla sp.* | Near the coast of Kundingarengkeke Island, Indonesia, at a depth of 16-20 ft. | acantholides (A-E); luffariellolide;  25-*O*-methylluffariellolide and  25-*O*-ethylluffariellolide | Acantholides D and E were not tested as antimicrobial. Acantholides E was cytotoxic against the mouse lymphoma L5187Y cell line. Acantholides B; luffariellolide and its 25-*O*-methyl congener were active against the Gram-positive bacteria *Staphylococcus aureus* and *Bacillus subtilis*, the Gram-negative bacterium *Escherichia coli*, the yeast *Candida albicans*, and the plant pathogenic fungus *Cladosporium herbarum.* | [[4](#_ENREF_4)] |
|  |  |  | Near Palau Badi, Makassar, Sulawesi, Indonesia, at a depth of 10-15 m | (+)-makassaric acid and  (+)-subersic acid | Inhibitors of the protein kinase MK2 with IC_50_ values of 20 and 9.6 µM, respectively. | [[5](#_ENREF_5)] |
| 5 | Sponge | *Acanthostrongylophora ingens* | Ti Toi and Bajotalawaan, North Sulawesi, Indonesia at a depth of 10 m | acantholactam; pre-neo-kauluamine; manzamine A and neo-kauluamine | Neo-kauluamine was active against HeLa cells, with an IC_50_ value of 5.4 μM.  Neo-kauluamine and pre-neo-kauluamine inhibited the proteasome with IC_50_ values of 0.13 and 0.34 μM, respectively, while manzamine A was less active (IC_50_ = 2.0 μM) and acantholactam was a very weak inhibitor (IC_50_ = 33 μM). Manzamine A, neo-kauluamine, and pre-neo-kauluamine exhibited 80−92% inhibition of accumulation of cholesterol esters, whereas acantholactam showed no inhibition. | [[6](#_ENREF_6)] |
|  |  |  | Ujung Pandang, Indonesia | manzamine A; 8-hydroxymanzamine A; manzamine F and 1-deoxyircinol A | Manzamine A ; 8-hydroxymanzamine A and manzamine F showed anti malarial, antibacterial, antiparasitic activity. The new compound, 1-deoxyircinol A has not yet been tested. 8-Hydroxymanzamine A in a single intraperitoneal dose of 100 μmoles/kg effectively reduced parasitemia. | [[7](#_ENREF_7)] |
|  |  |  | Indonesia | annomontine; ingenine A and ingenine B | Annomontine and ingenine B showed cytotoxicity against the murine lymphoma L5178Y cancer line with ED_50_ 7.8 and 9.1 µg/mL respectively, while ingenine A showed weak activity. | [[8](#_ENREF_8)] |
|  |  |  | Ambon, Indonesia at the depth of 10 m | ircinal E; manzamine A;  8-hydroxymanzamine A; manzamine F; manzamine A *N*-oxide;  3,4-dihydromanzamine A *N*-oxide and nakadomarin A | Ircinal E; manzamine A;  8-hydroxymanzamine A; manzamine F; manzamine A *N*-oxide;  3,4-dihydromanzamine A *N*-oxide exhibited strong to moderate cytotoxicity against murine lymphoma L5178Y cancer line with IC_50_ value 2.8 to 21.7 µM. | [[9](#_ENREF_9)] |
|  |  |  | Sulawesi Island, Indonesia at depth of 12 m | ingenines C; ingenines D; annomontine; acanthomine A and  1-hydroxy-3,4-dihydronorharman | Ingenines C and ingenines D exhibited cytotoxic activities against MCF7 and HCT116 with IC_50_ values of 4.33 and 6.05 and 2.90 and 3.35mM, respectively compared to doxorubicin (IC_50_ 0.23 and 0.39 mM, respectively).Both compound also showed weak activity towards A549 cancer cell line. | [[10](#_ENREF_10)] |
|  |  |  | South Sulawesi and Makassar (Ujong Pandang), Indonesia  Spermonde Archipelago, north-west Lankai Island, reef slope at a depth of 14 m depth | epi-tetradehydrohalicyclamine B ; tetradehydrohalicyclamine B; chloromethyltetradehydrohalicyclamine B; diketopiperazines 6–12; acanthocyclamine A; halicyclamine B and chloromethylhalicyclamine B | Acanthocyclamine A and halicyclamine B showed a selective antimicrobial activity against *E. coli* and *S. aureus*, respectively (diameter inhibition of 12 and 10 mm at 100 µg/disk, respectively). Chloromethylhalicyclamine B showed a selective inhibitory activity against the protein kinase CK1δ/ε with an IC_50_ value of 6 µM. Diketopiperazines **6** displayed a selective kinase inhibitory activity against CDK2/cyclin A with an IC_50_ value of 1 µM. Acanthocyclamine A showed an inhibition of amyloid β-42 production induced by aftin-5 at 26 µM, without cytotoxicity at this dose. | [[11](#_ENREF_11)] |
| 6 | Sponge | *Acanthostrongylophora*  *sp.* | Manado, Indonesia | 12,34-oxamanzamine E;  8-hydroxymanzamine J; and  6-hydroxymanzamine E | Active against malaria, *Mycobacterium tuberculosis*, Leishmania, HIV-1, and AIDS, cytotoxicity, insecticidal, antibacterial, antiinflammatory, antiinfective, and antiparasitic. | [[12](#_ENREF_12)] |
|  |  |  | Knife Cape Manado, Indonesia at vertical slopes between 33 and 40 m | 12,28-oxamanzamine E;  12,34-oxa-6-hydroxymanzamine E;  8-hydroxymanzamine B;  12,28-oxaircinal A; ircinal A; ircinol A; xestomanzamine A; manzamines A, E, F, J, and Y; manadomanzamines A and B; *neo*-kauluamine;  8-hydroxymanzamine A and Manzamin A *N-*oxide. | The isolated compounds were active against malaria, leishmania, tuberculosis, and HIV-1 are also presented. Manzamine Y showed significant inhibitory activity of GSK3, an enzyme implicated in Alzheimer’s disease pathology. | [[13](#_ENREF_13)] |
|  |  |  | Black Reef Point, Manado Bay, Suluwasi, Indonesia at the depth of 6 and 23 m | manadomanzamines A and B | Manadomanzamines A and B exhibited strong activity against *Mycobacterium tuberculosis* (Mtb) with MIC values of 1.9 and 1.5 µg/mL, respectively. Both compounds also exhibit activities against human immunodeficiency virus (HIV-1) and AIDS opportunistic fungal infections. | [[14](#_ENREF_14)] |
|  |  |  | Indonesia | manzamine A | GSK-3*β* inhibitors decreasing tau hyperphosphorylation in human neuroblastoma cell lines, a demonstration of its ability to enter cells and interfere with tau pathology. | [[15](#_ENREF_15)] |
|  |  |  | Indonesia | acantholactone | The low isolated yield of acantholactone from the sponge extract limited its biological evaluation | [[16](#_ENREF_16)] |
| 7 | Sponge | *Agelas linnaei* | Near Peniki E Island, Seribu Islands (also known as  Thousands Islands), in Northwest-Java, Indonesia at the depth of 15 m | midpacamide; agelongine;  methyl-4,5-dibromocarboxylic acid; methyl-4,5-dibromocarboxylic acid methyl ester; dibromphakellin HCl; dibromphakellin; dibromohydroxyphakellin HCl;  4-(4,5-dibromo-1-methylpyrrole- 2-carboxamido)-butanoic acid;  agelanin A and B; agelanesin A-D and mauritamide B-D | The compounds showed activity not only as anti-fouling agents but also as inhibitors of bacterial biofilms. These compounds also exhibited cytotoxic activity against L5178Y mouse lymphoma cells with IC_50_ values between 9.25 and 16.76 µM. | [[17](#_ENREF_17)] |
| 8 | Sponge | *Agelas nakamurai* | The coast of Menjangan Island (North of Bali Island) at a depth of 12 m | agelasine D; ageloxime-D; mukanadin-C and agelasidine C | The compounds exhibited activity not only as anti-fouling agents but also as inhibitors of bacterial biofilms.The diterpenoid alkaloids (-)-agelasine D and (-)-ageloxime D showed IC_50_ value of 4.03 and 12.5 µM in the cytotoxicity assay, respectively, indicating that presence of the oxime function decreases activity towards lymphoma cells. | [[17](#_ENREF_17)] |
|  |  |  | Near Menjangan Island (North of Bali Island) in Indonesia at a depth of 12 m | mukanadin C; 4-(4-bromo-1*H*-pyrrole-2-carboxamido)-butanoic acid;  4-bromopyrrole-2-carboxamide;  4-bromopyrrole-2-carboxylic acid and agelasine I | The compounds showed tyrosine kinase inhibition, and antifouling activity and also known to posses antibacterial and antifungal activity. Agelasine I was reported to have weak antifungal activity but was inactive as antibacterial. Mukanadin C; 4-bromopyrrole-2-carboxamide; and 4-bromopyrrole-2-carboxylic acid showed antibacterial activity, whereas 4-(4-bromo-1*H*-pyrrole-2-carboxamido)butanoic acid, the new compound, have not been tested yet. Agelasine I has antifungal activity | [[7](#_ENREF_7)] |
| 9 | Sponge | *Aplysinellidae sp.* | Manta Point, Sangalaki, Indonesia at a depth of 21.3 m. | purpuramine M–N; araplysillin VII–XI; hexadellin A; araplysillin II; araplysillin IV; purpurealidin I; aplysamine 4; purpuramine G | In biological screening, purpuramine M; araplysillin VII-XI; hexadellin A; araplysillin II; purpurealidin I; aplysamine 4 and purpuramine G showed moderate inhibition of the aspartic protease, BACE1 (memapsin-2). | [[18](#_ENREF_18)] |
| 10 | Sponge | *Aplysinella strongylata* | Tulamben Bay, Bali, at a depth of approximately 20 m | psammaplysins A and B; psammaplysins E;  19-hidroxypsammaplysins E; psammaplysins K; psammaplysins K dimethoxy acetal; psammaplysins L-W; 19-hydroxypsammaplysins P;  19-hydroxypsammaplysins Q;  19-hydroxypsammaplysins S-V and ceratinamides A and B | 19-Hydroxypsammaplysin E displayed modest in vitro growth inhibition of chloroquine-sensitive *P. falciparum* parasites | [[19](#_ENREF_19)] |
| 11 | Sponge | *Axinella carteri* | Shark Point, Derawan Island, Indonesia, at a depth of 20 m | 3*β*-(hydroxymethyl)- A-nor-5*α*-cholest-14-en-16-one and dibromoisophakellin | Moderate cytotoxic activity (5 µg/mL) against tumour cell lines (P 388, A 549 and HT 29) but the bioactivity of the sterol was not assessed. | [[20](#_ENREF_20)] |
|  |  |  | Java and Sumatera Island, Indonesia | guanidine alkaloid hymenialdisine; debromohymenaldisine and 3-bromohymenialdisine | The compounds showed insecticidal activity towards nenonate larvae of polyphagus pest insect *Spodoptera littoralis.* The compounds were also equitoxic. | [[21](#_ENREF_21)] |
| 12 | Sponge | *Axinyssa aculeata* | Ujung Pandang, Indonesia | indole-3-carboxaldehyde and curcuphenol | Curcuphenol reported as having antibacterial, antifungal, and cytotoxic activity. It inhibited gram positive bacteria *Staphylococcus aureus* with and the yeast *Candida* *albicans* | [[7](#_ENREF_7)] |
| 13 | Sponge | *Axinyssa aplysinoides* | Latondu Island, Taka Bonerate, Indonesia | boneratamide A and C have been isolated as their methyl esters. | Boneratamide B was inactive in the antimitotic assay. | [[22](#_ENREF_22)] |
| 14 | Sponge | *Axynissa sp.* | Maluku, Indonesia | (+)-curcuphenol and (+)-curcudiol | (+)-Curcuphenol showed SRC protein kinase inhibition with an IC_50_ value of 7.8 µg/mL. (+)-Curcudiol inhibited FAK with an IC_50_ value of 9.2 µg/mL. | [[23](#_ENREF_23)] |
| 15 | Sponge | *Biemna fortis* | Labuanbajo, West Flores, Nusa Tenggara Timur, Indonesia | labuanine A;  9-hydroxybenzo[*b*]pyrido[4,3,2-de][1,10]phenanthrolin-8(8*H*)-one;  9-aminobenzo[*b*]pyrido[4,3,2-*d,e*][1,10]phenanthrolin-8(8*H*)-one and biemnadin | Neuronal differentiation inducers against a murine neuroblastoma cell line, Neuro 2A | [[24](#_ENREF_24)] |
| 16 | Sponge | *Callyspongia aerisuza* | Ambon, Indonesia at a depth of 4–5 m | callyaerins A–F and H | Callyaerins A–F and H showed biological activity in antibacterial assays and in various cytotoxicity assays employing different tumour cell-lines (L5178Y, HeLa, and PC12). Callyaerins E and H exhibited strong activity against the L5178Y cell line with ED_50_ values of 0.39 and 0.48 µM, respectively. Callyaerins A showed strong inhibitory properties towards *C. albicans.* | [[25](#_ENREF_25)] |
|  |  |  | Ambon, Indonesia at a depth of 4–5 m | callyaerin G | The compound exhibit cytotoxic activity when tested against different cancer cell lines | [[26](#_ENREF_26)] |
|  |  |  | Three different locations in Indonesia as indicated:  Makassar, S. Sulawesi (TF23, TF90); Lembeh, N. Sulawesi (SP3); and Ambon, Maluku (TF40). | callyaerins I−M and callyaerins A−G | Callyaerins A and B showed potent anti-TB activity with MIC_90_ values of 2 and 5 μM, respectively. Callyaerins C was found to be less active, with an MIC_90_ value of 40 μM. Callyaerins A showed the strongest anti-TB activity, but not cytotoxic to THP-1 or MRC-5 cells (IC_50_ > 10 μM). | [[27](#_ENREF_27)] |
|  |  |  | Indonesia | callyspongiolide | Strong cytotoxicity against human Jurkat J16 T and Ramos B lymphocytes | [[28](#_ENREF_28)] |
| 17 | Sponge | *Callyspongia (Euplacella) biru de Voogd* | Spermonde Archipelago, Southwestern Sulawesi, Indonesia at a depth of  10–14 m | amphitoxin 3-alkylpyridine | Pharmaceutically active compound, and which concentrations are needed for drug production | [[29](#_ENREF_29)] |
| 18 | Sponge | *Callyspongia pseudoreticulata* | South Sulawesi, Spermonde Archipelago, off Ujung Pandang, Indonesia | eicosa-1,19-diyne-3,18-diol-4,16-diene | Toxic against nauplii of the brine shrimp *Artemia salina* (LD_50_ 5 µg/mL). | [[30](#_ENREF_30)] |
| 19 | Sponge | *Candidaspongia sp.* | Kupang, West Timor, East Nusa Tenggara, Indonesia at the depth of 15–25m | candidaspongiolide along with two new macrolides A and B | All of the compounds exhibited potent cytotoxicity, IC_50_ 37, 4.7, and 19 µg/mL, against NBT-T2 cells. Candidaspongiolide showed stronger growth inhibition (GI_50_ 14 µg/mL) than the core compound (42 µg/mL) | [[31](#_ENREF_31)] |
| 20 | Sponge | *Carteriospongia foliascens* | Near Palau Barang Lompo, Makassar, Sulawesi, Indonesia at a depth of 10 m | Two new 20,24-bishomo-25-norscalaranes A and B; 12*α* acetoxy-20,24*β*-dimethylscalar-17-eno-25,24-lactone and (12a,24*S*)-12-[(3-hydroxypentanoyl)oxy]-20,24-dimethyl-25-oxoscalar-15,17-dien-25,24-olide | Promising activity in the RCE protease assay | [[32](#_ENREF_32)] |
| 21 | Sponge | *Clathria basilana* | Ambon, Indonesia | microcionamides C and D; gombamides B, C, and D; (*E*)-2-amino-3-methyl-*N*-styrylbutanamide and microcionamide A | Microcionamides A, C, and D showed *in vitro* cytotoxicity against lymphoma (Ramos) and leukemia cell lines (HL-60, Nomo-1, Jurkat J16), as well as against a human ovarian carcinoma cell line (A2780) with IC_50_ values ranging from 0.45 to 28 μM. Microcionamides C and A inhibited bacterial growth of *Staphylococcus aureus* and *Enterococcus faecium* with minimal inhibitory concentrations between 6.2 and 12 μM. | [[33](#_ENREF_33)] |
| 22 | Sponge | *Clathria bulbotoxa* | Samalona Island, South Sulawesi Sea, Indonesia at a depth between 0.5 and 3 m | crambescidins 345; crambescidins 361; crambescidins 373; crambescidins 359; crambescidins 657 and crambescidins 800 | All of the compounds showed citotoxicity with an IC_50_ value lower than 10 µM, with crambescidins 657 and crambescidins 800 exhibit the strongest activity with IC_50_ values of 12 and 48 Nm, respectively. Crambescidins 345; crambescidins 361; crambescidins 373 and crambescidins 359 showed higher anti-oomycete activity with MID of 50 µg/disk than crambescidins 657 and crambescidins 800 (MID 100 µg/disk and higher). | [[34](#_ENREF_34)] |
| 23 | Sponge | *Clathria* sp. | Reef slope area of the Bintang Samudra Marine Education Park, Southeast Sulawesi, Indonesia, at a depth of 2–10 m | 3*β*-(butyryloxymethyl)-A-nor-5*α*-cholestane; 3*β*-(acetoxymethyl)-A-nor-5*α*-cholestane;  3*β*-(hydroxymethyl)-A-nor-5*α*-cholest-15-ene and 3*β*-(hydroxymethyl)-A-nor-5*α*-cholestane | The isolated compounds were evaluated for antibacterial activity against *E. coli*, *S. aureus*, and antifungal activity against *C. albicans*. However, none of the tested compounds exhibited antibacterial and antifungal activities as compared to chloramphenicol and ketoconazole, respectively. | [[35](#_ENREF_35)] |
| 24 | Sponge | *Coelocarteria cfr. singaporensis* | Bunaken Marine Park, North Sulawesi, Indonesia | coelodiol and coeloic acid | Inhibit the growth of MKN-45 cell line (human gastric adenocarcinoma). | [[36](#_ENREF_36)] |
| 25 | Sponge | *Corticium simplex* | Indonesia | cortistatin A | Anti-angiogenic | [[37](#_ENREF_37)] |
|  |  |  | Indonesia | cortistatins J; cortistatins K and cortistatins L | Anti angiogenic and cytostatic anti-proliferative activity against human umbilical vein endothelial cells (HUVECs) | [[38](#_ENREF_38)] |
| 26 | Sponge | *Corticium sp.* | Bunaken Island, Sulawesi, Indonesia at a depth of 20 m at | *N,N-*dimethyl-4*β*-hydroxy-3-*epi*-plakinamine A and *N*-acetyl-4*β*-hydroxy-3-*epi*-plakinamine A | Antimicrobial activity against *Batillus subtilis* | [[39](#_ENREF_39)] |
| 27 | Sponge | *Dactylospongia elegans* | Indonesia | dictyoceratin-C; smenospondiol; smenospongine ; smenospongorine; ilimaquinone; 5-epi-smenospongine; 5-epi-smenospongorine;  5-epi-smenospongidine; dysideamine and bolinaquinone | Dictyoceratin-C inhibited proliferation of human prostate cancer DU145 cells selectively under hypoxic condition in a dose-dependent manner at the concentrations ranging from 1.0 to 10 µM. Smenospondiol exhibited the similar hypoxia-selective growth inhibitory activity against DU145 cells, and the *para*-hydroxybenzoyl ester moiety would be important for hypoxia-selective growth inhibitory activity of dictyoceratin-C. In addition, the mechanistic analysis of dictyoceratin-C revealed that the 10 µM of 1 inhibited accumulation of Hypoxia-Inducible Factor-1a under hypoxic condition | [[40](#_ENREF_40)] |
|  |  |  | Towo’e Beach Tahuna Bay, Sangihe Islands North Sulawesi Province Indonesia at the depth of 4 m | aminoquinone nakijiquinone V; illimaquinone; smenospongine and dyctioceratine C | All compounds lacked antimicrobial activity against *Escherichia coli.* Ilimaquinone; smenospongine and dyctioceratine C exhibited modest antimicrobial activity against *Bacillus megaterium* with MIC values of 32 µg/mL, 32 µg/mL, and 64 µg/mL, respectively. Ilimaquinone; smenospongine and dyctioceratine C inhibit *Micrococcus luteus* with a MIC of 32 µg/mL each. | [[41](#_ENREF_41)] |
| 28 | Sponge | *Dasychalina sp.* | Bunaken Marine Park, Manado | haplosamate A and desulfohaplosamate | Desulfohaplosamate showed a selective affinity for CB2 receptors in the low µM range. | [[42](#_ENREF_42)] |
| 29 | Sponge | *Diacarnus megaspinorhabdosa* | Near Langkai Island in the Spermonde Archipelago, SW Sulawesi, Indonesia at a depth of 4 m-152 m. | euplectellodiol and methyl 3-acetoxy-6-hydroxy-2,6-dimethyl-8-(2,2-dimethyl-6-methylenecyclohexyl)-octanoate | Methyl 3-acetoxy-6-hydroxy-2,6-dimethyl-8-(2,2-dimethyl-6-methylenecyclohexyl)-octanoate showed no antiproliferative activity (IC_50_>10 µM). | [[43](#_ENREF_43)] |
| 30 | Sponge | *Dysidea herbacea* | The shores of the Air island of West Sumatra, Indonesia | 3,4,5-tribromo-2-(2′-bromophenoxy)phenol;  3,5,6-tribromo-2-(2′-bromophenoxy)phenol;  3,4,6-tribromo-2-(2′-bromophenoxy)phenol;  3,5,6-tribromo-1-(2′-bromophenoxy)-2-benzene methyl ether;  2-(2-bromophenoxy)-3,4,5,6-tetrabromophenol and  2-(2,4-dibromophenoxy)-3,4,5,6-tetrabromopheno | All of the compounds were active against the Gram-positive bacteria *Bacillus subtilis* and the phytopathogenic fungus *Cladosporium cucumerinum*. The isolated polybrominated compounds were also active in the brine shrimp lethality test. In the latter bioassay, compounds 3,4,5-tribromo-2-(2′-bromophenoxy)phenol and 2-(2,4-dibromophenoxy)-3,4,5,6-tetrabromopheno were the most active with LC_50_ values of 0.96 [SE ±0.19] and 0.94 [SE ± 0.70] µg/mL, respectively. | [[44](#_ENREF_44)] |
| 31 | Sponge | *Dysidea sp.* | Near Palau Sintok, Karimunjawa archipelago, Indonesia at a depth of ~15 m | sintokamides A to E | Sintokamides A is an inhibitor of *N*-terminus transactivation of the androgen receptor in prostate cancer cells | [[45](#_ENREF_45)] |
|  |  |  | Biak, Papua-Indonesia at 20–35 m depths | biaketide ; debromoantazirine; antazirine; dysidazirine; neoavarol and frondosin | Biaketide and debromoantazirine showed moderate cytotoxicity against NBT-T2 cells with IC_50_ values of 8.3 and 4.7 µg/mL, respectively | [[46](#_ENREF_46)] |
| 32 | Sponge | *Halichondria sp.* | Tulamben Bay, Bali, Indonesia at a depth of 20 m | tetradehydrohaliclonacyclamine A; bis-*N-*oxide; *N*-oxide derivative and 2-*epi* isomer | Tetradehydrohaliclonacyclamine A exhibited for antitumour activity against the P388 cell line with an IC_50_ of 1.8 µg/mL whereas the bis-*N-*oxide was not active at the concentrations tested. | [[47](#_ENREF_47)] |
| 33 | Sponge | *Haliclona sp.* | Lembeh Island, Bitung, Indonesia | lembehynes B and C | Exhibited neuritogenic activity against nuroblaastoma cell line (analogue) | [[48](#_ENREF_48)] |
|  |  |  | Knife Cape, Manado Bay, Indonesia at vertical slopes between 33 and 40 m | 12,34-oxamanzamine A;  12,34- oxamanzamine E; manzamine A ; 8-hydroxymanzamine A;  6-deoxymanzamine X; manzamine E; manzamine X; manzamine F; norharman; thymine; 2’,3’-didehydro-2’,3’-dideoxyuridine; uracil; thymidine; 2’-deoxyuridine;  32,33-dihydro-31-hydroxymanzamine A;  32,33-dihydro-6-hydroxymanzamine A-35-one;  des-*N-*methylxestomanzamine A;  32,33-dihydro-6,31-dihydroxymanzamine A and  1,2,3,4-tetrahydronorharman-1-one | Antileishmanial Anti-malaria, and anti-TB | [[49](#_ENREF_49)] |
|  |  |  | Mayu Island, Indonesia | haliclotriol A and B; and haliclotriol A triacetate | The compounds showed antimicrobial activity against *B. subtilis* and *S. aureus.* No cytotoxicity responses were obtained for haliclotriol A and B, weak antimicrobial activity was observed for haliclotriol B at 1 µg disk against *B. subtilis* and *S. aureus*, respectively. | [[50](#_ENREF_50)] |
|  |  |  | Lembeh Island, Bitung, Indonesia | lembehyne A | Induced neuritogenesis in pheochromocytoma PC12 cells and neuroblastoma Neuro 2A cells at 2 and 0.1 µg/mL. | [[51](#_ENREF_51)] |
|  |  |  | Sulawesi Island, Indonesia | kendramine A | Modulator of multidrug resistance (MDR) in tumor cells. Kendarimide A completely reversed the resistance to colchicine in KB-C2 cells, 10 human carcinoma cell line overexpressing P-gp, at a 6 µM concentration. | [[52](#_ENREF_52)] |
|  |  |  | Between Alor and Pantar Islands, Nusa Tengara Timur, Indonesia at a depth range of 20–35 m | polyunsaturated brominated fatty acid possessing acetylenic bonds | Moderate cytotoxicity against cultured cells. The compound showed cytotoxicity against NBT-T2 rat bladder epithelial cells, and the IC_50_ value was estimated to be 36 μg/mL. | [[53](#_ENREF_53)] |
|  |  |  | Indonesia at depths of 15–30 m | 3-dodecyl pyridine containing a terminal cyano group | Moderate cytotoxity against tumour cell lines A549, MCF-7 and Hela with IC_50_ values of 41.8, 48.4 and 33.2 µM, respectively. | [[54](#_ENREF_54)] |
|  |  |  | Baubau, Buton Island, South-East Sulawesi, Indonesia, at around 20 m depth | halioxepine | Moderate cytotoxicity against NBT-T2 cells with IC_50_ 4.8 µg/mL and also antioxidant activity against 1,1-diphenyl-2-picrylhydrazyl (DPPH) with IC_50_ 3.2 µg/mL. | [[55](#_ENREF_55)] |
|  |  |  | Indonesia | papuamine and haliclonadiamine | Inhibited cell proliferation of six human cancer cell lines with IC_50_ values of 0.93–1.50 and 1.00–4.44 µM, respectively. Papuamine and haliclonadiamine accumulated lymphoma U937 cells at sub-G1 phase and induced a condensation of chromatin and fragmentation of nucleus. | [[56](#_ENREF_56)] |
| 34 | Sponge | *Hippospongia sp.* | Barangcadi Island, Ujung Pandang, Sulawesi, Indonesia | barangcadoic acid A; and rhopaloic acids D to G | Barangcadoic acid A; rhopaloic acids D and E had IC_50_ values of ≈ 10 µg/mL in the RCE protease assay. Barangcadoic acid A; rhopaloic acids D and E had IC_50_ values of ≈ 1-2 µg/mL against the CaCo cells. | [[57](#_ENREF_57)] |
| 35 | Sponge | *Histodermella sp.* | Manado Bay, Indonesia at the depth between 20 and 120 ft | makaluvamine G; makaluvamine A; makaluvamine C; damirones A and damirones B | Makaluvamine G was moderately cytotoxic to several tumor cell lines, with an IC_50_ value 0.50 µg/mL against P388 (murine leukimia), A549 (human non-small cell lung cancer), HT-29 (human colon cancer), and MCF-7 (Human breast cancer), and 0.35 µg/mL against KB (human oral epidermoid carcinoma). Displayed moderate immunomodulatory activity in the mixed lymphocyte reaction (IC_50_=0.28 µg/mL), with only mild cytotoxicity to resting lymphocytes (IC_50_=0.28 µg/mL). Makaluvamines A, C, E was found to be a moderate inhibitor of topoisomerase-I (IC_50_=3.0 µM), compound **5** showed moderate inhibition to RNA (IC_50_= 15 µM), DNA (IC_50_=15 µM), and protein (IC_50_=21 µM) synthesis. | [[58](#_ENREF_58)] |
| 36 | Sponge | *Hyatella sp.* | Indonesia | laulimalide | Antitumor. | [[59](#_ENREF_59)] |
|  |  |  | Indonesia | laulamalide | Cytotoxic. | [[60](#_ENREF_60)] |
|  |  |  | Indonesia | laulimalide | Cytotoxic and fungicidal activities. | [[61](#_ENREF_61)] |
|  |  |  | Manado, northern Sulawesi, Indonesia, at a depth of 20 m | laulimalide and isolaulimalide | Laulimalide displays potent cytotoxicity, IC_50_ = 15 µg/mL, against the KB cell line. | [[62](#_ENREF_62)] |
|  |  |  | Coral reef at the Lembeh Strait, North Sulawesi, Indonesia, | hyattellactones A and B; and phyllofolactones F and G | Hyattellactones A and phyllofolactones F inhibited PTP1B activity with IC_50_ values of 7.45 and 7.47 µM, respectively. Hyattellactones B and phyllofolactones G showed much reduced activity than the 24*R*-isomers. | [[63](#_ENREF_63)] |
| 37 | Sponge | *Hyrtios erectus* | Indonesia | diketotriterpenoid | Antimiotic | [[64](#_ENREF_64)] |
|  |  |  | Inner reef of Ujung Padang, Sulawesi, Indonesia at a depth of 10-15 m | diketotriterpenoid | Antimiotic | [[65](#_ENREF_65)] |
| 38 | Sponge | *Hyrtios sp* | Togian Island in Tomini Bay, North Sulawesi, Indonesia | puupehenone; (+)-(5*S*,8*S*,9*R*,10*S*)-20-methoxypuupehenone;  (+)-(5*S*,8*S*,10*S*)-20-methoxy-9,15-ene-puupehenol and (+)-(5*S*,8*S*,9*R*,10*S*)-15,20-dimethoxypuupehenol | None of the three compounds reported above were active in the Valeriote *in vitro* soft-agar disk diffusion assay screen employing human L1210 leukemia or murine colon 38 or human colon H116 tumors | [[66](#_ENREF_66)] |
| 39 | Sponge | *Ircinia dendroides* | Manado, Sulawesi, Indonesia at a depth of 3-40 m | waiakeamide | Preliminary bioassay data of this fraction showed strong activity against P388 cells at an IC_50_ of 0.054 µg/mL. | [[67](#_ENREF_67)] |
| 40 | Sponge | *Ircinia* sp. | The Thousand Islands, Indonesia | haloirciniamide A and seribunamide A | Haloirciniamide A and seribunamide A failed to show significant cytotoxicity against four human tumor cell lines, lung (A-549), colon (HT-29), breast (MDA-MB-231), and pancreas PSN-1. Both compounds was unable to inhibit the enzyme topoisomerase I or impair the interaction between programmed cell death protein PD1 and its ligand, PDL1. | [[68](#_ENREF_68)] |
| 41 | Sponge | *Jaspis splendens* | Kalimantan (Indonesia), namely Samama, Panjang, and Shoal Islands, at 10 m depths | jaspamide; jaspamide Q and jaspamide R | All of the compounds exhibited potent activities with IC_50_ values in the µg/mL range (<0.1 μg/mL, <0.16 μM). The cytotoxicity of the compounds was still need to be studied further. | [[69](#_ENREF_69)] |
| 42 | Sponge | *Kaliapsis sp.* | Menjangan Island, West Bali National Park at 20 m below sea surface | theonellapeptolide Id | *In vitro* cytotoxicity of isolate theonellapeptolide Id. was 0.18, 7.9, and 5.8 µg/mL on Myeloma, T47D, HeLa, and Raji cells, respectively. | [[70](#_ENREF_70)] |
|  |  |  | Menjangan Island, West Bali, Indonesia | 1-(tetrahydro-4-hydroxy-5-(hydroxymethyl)furan-2-yl)-5-methyl pyrimidine-2,4(1*H*,3*H*)-dione | Cytotoxic against Myeloma cell (IC_50_=10.3 µg/mL), T47D cell (IC_50_=8.3 µg/mL), HeLa cell (IC_50_=16.5 µg/mL), Raji cell (IC_50_=7.8 µg/mL) | [[71](#_ENREF_71)] |
| 43 | Sponge | *Lamellodysidea herbacea* | Sangiang Island, West Java, Indonesia | 2,3,5-tribromo-6-(3′,5′-dibromo-2′-methoxyphenoxy)phenol;  2,5-dibromo-6-(3′,5′-dibromo-2′-hydroxyphenoxy)phenol;  2,4,5-tribromo-6- (5′-bromo-2′-hydroxyphenoxy)phenol and  2,4,5-tribromo-6-(3′,5′- dibromo-2′-hydroxyphenoxy)anisole | All compounds were active against the grampositive bacterium *B. subtilis* in the range of 1-10 µg/disk. 2,5-Dibromo-6-(3′,5′-dibromo-2′-hydroxyphenoxy)phenol was most active, giving clear zones of inhibition of 20, 20, 13, and 7 mm at 10, 5, 1, and 0.1 µg/disk, respectively. However, in the cytotoxicity assay against NBT-T2 rat bladder epithelial cells, these compounds showed no significant activities (IC_50_ >15 µg/mL). | [[72](#_ENREF_72)] |
|  |  |  | Manadotua Island, Indonesia at a depth of 10 m | lamellodysidines A and B;  *O*,*O*-dimethyllingshuiolide A;  11-epi-*O*,*O*-dimethyllingshuiolide A and *O*-methyl nakafuran-8 lactone | Biological activities of all the isolated compounds were tested including cytotoxicity, antimicrobial activities, inhibitory activity of the cholesterol ester accumulation in macrophages, inhibitory activity of the RANKL− induced formation of multinuclear osteoclasts, and inhibitory activities of the ubiquitin−proteasome system (proteasome, E1, Ubc13 (E2)−Uev1A interaction, p53−Mdm2 (E3) interaction, and USP7). However, no significant activity was detected for these compounds. | [[73](#_ENREF_73)] |
|  |  |  | Pari Island, Kepulauan Seribu, Indonesia at 3–8 m depths. | 3,4,5-tribromo-2-(20,40-dibromophenoxy)phenol and  3,4,5,6-tetrabromo-2-(20,40-dibromophenoxy)phenol | The compounds were highly active against all tested strains except *E. coli* (MIC > 66.7 μg/mL). Both compounds strongly inhibited *B. subtilis* (MIC 0.5 μg/mL), displaying a five times lower MIC than the positive control tetracycline (2.8 μg/mL). | [[74](#_ENREF_74)] |
|  |  | *Lamellodysidea* sp. (cf.*L. Herbacea*) | Manado, North Sulawesi, Indonesia | bicyclolamellolactone A; and lamellolactones A and B | All of the compounds inhibited bone morphogenic protein (BMP)-induced alkaline phosphatase activity in mutant BMP receptor-carrying C2C12 cells with IC_50_ values of 51, 4.6, and 20 μM, respectively. | [[75](#_ENREF_75)] |
| 44 | Sponge | *Lanthella basta* | Indonesia | bastadin 6 | Show the potent and selective anti-proliferative activity against human umbilical vein endothelial cells (HUVECs). | [[76](#_ENREF_76)] |
| 45 | Sponge | *Lendenfeldia sp.* | Indonesia at 2 m depth | naphthalene dimer: (*S*)-2,2′-dimethoxy-1,1′-binaphthyl-5,5′,6,6′-tetraol; furanolipid;  16*β*,22-dihydroxy-24-methyl-24-oxoscalaran-25,12*β*-olactone;  24-methyl-12,24,25-trioxoscalar-16-en-22-oic acid; PHC-1 and  12,16-dihydroxy-24-methylscalaran-25,24-olide | 16*β*,22-Dihydroxy-24-methyl-24-oxoscalaran-25,12*β*-olactone and  12,16-dihydroxy-24-methylscalaran-25,24-olide were found to inhibit tumor cell growth with very little specificity for individual tumor cell lines (average GI_50_ values 20.4 and 19 μM, respectively.  24-Methyl-12,24,25-trioxoscalar-16-en-22-oic acid; PHC-1 was significantly more potent (mean GI_50_ 1.17 μM; mean LC_50_ 11.2 μM) than either of the other two related homoscalaranes. 24-Methyl-12,24,25-trioxoscalar-16-en-22-oic acid; PHC-1 also produced a more distinct cell line selectivity pattern of tumor cell growth suppression (GI_50_ range 0.26 to 3.55 μM) and cytotoxicity (LC_50_ range 3.4 to >100 μM). | [[77](#_ENREF_77)] |
| 46 | Sponge | *Leucetta chagosensis* | Near the coast of Kapoposang Island, at a depth of 41 ft. | naamine F; naamine G; kealiinine A; kealiinine B and kealiinine C | Naamine A and G exhibited strong antifungal activity against the phytopathogenic fungus *Cladosporium* *herbarum* and also showed mild cytotoxicity against mouse lymphoma (L5178Y) and human cervix carcinoma (HeLa) cell lines. In the brine shrimp assay, kealiinine A was more active than naamine G. | [[78](#_ENREF_78)] |
|  |  |  | North Sulawesi, Indonesia at a depth of 10 m | naamidines H and I | Naamidines H and I showed cytotoxicity against HeLa cells with IC_50_ values of 5.6 and 15 μg/mL, respectively. | [[79](#_ENREF_79)] |
| 47 | Sponge | *Lissodendoryx (Acanthodoryx) fibrosa* | North Sulawesi, Indonesia at a depth of 5 m | manadosterols A and B | Inhibited the Ubc13-Uev1A interaction with IC_50_ values of 0.09 and 0.13 μM, respectively. | [[80](#_ENREF_80)] |
| 48 | Sponge | *Luffariella sp.* | Manado Bay, Sulawesi, Indonesia | germacrene alcohol; aaptamine and hexacyclic terpene | *In vitro* activity against KB cancer cell line | [[81](#_ENREF_81)] |
| 49 | Sponge | *Luffariella variabilis* | North Sulawesi, Indonesia at a depth of 10 m | variabines A and B | Variabines B inhibited chymotrypsin-like activity of the proteasome and Ubc13 (E2)–Uev1A interaction with IC_50_ values of 4 and 5 µg/mL, respectively, whereas variabines A had little effect on the activity or interaction | [[82](#_ENREF_82)] |
| 50 | Sponge | *Melophlus sarasinorum* | Near the shores of Makassar, Sulawesi  Island, Indonesia. | sarasinosides A1; sarasinosides A3; sarasinosides I1; sarasinosides I2; sarasinosides H2; sarasinosides J; sarasinosides K; sarasinosides L and sarasinosides M | Sarasinosides A1 was selectively and strongly active against the yeast *Saccharomyces cerevisiae* and was inactive against *B. subtilis* and *E. coli*.  Sarasinosides J was strongly active against *S. cerevisiae* and showed moderate antibacterial activity toward *B. subtilis*. Sarasinosides A1 has also been reported to possess mild cytotoxicity to the P388 lymphocytic leukemia cell line. | [[83](#_ENREF_83)] |
|  |  |  | Siladen, Indonesia at a depth of 10 m | melophluosides A and B | Melophluosides A and B showed weak cytotoxicity against HeLa cells, with IC_50_ values of 11.6 and 9.7 mM, respectively.  Melophluosides A and B also showed no antimicrobial activities against bacteria (*Escherichia coli* and *Bacillus cereus*) or yeast (*Candida albicans*), even at 20 mM. | [[84](#_ENREF_84)] |
| 51 | Sponge | *Monanchora*  *ungiculata* | Indonesia | crambescidin 800 | Protected HT22 cells against glutamate-induced oxidative toxicity at 0.06 µM (EC_50_) concentration. also protected HT22 and neuroblastoma cells from the oxidative stress induced by a hypoxic condition or nitric oxide (NO). | [[85](#_ENREF_85)] |
| 52 | Sponge | *Mycale euplectelloides* | Near Tindila Island, North Sulawesi, Indonesia at a depth of 15 m | mycaperoxide and euplectellodiol | Euplectellodiol showed no cytotoxicity (IC_50_ >10 mM) indicating the importance of the 1,2-dioxane moiety for the observation of the bioactivity | [[43](#_ENREF_43)] |
| 53 | Sponge | *Mycale phyllophila* | Menjangan Island, Bali | 5-pentadecyl-1*H*-pyrrole-2-carbaldehyde and (6’*E*)-5-(6’pentadecenyl)-1*H*-pyrrole-2-carbaldehyde | Exhibited mouse lymphoma cell line (L5178Y) growth inhibition with IC_50_ 1.8 µg/mL. | [[86](#_ENREF_86)] |
| 54 | Sponge | *Neopetrosia cf exigua.* | Old Derawan Pier, Indonesia at a depth of 24 m | neopetrocyclamines A and B; papuamine and haliclonadiamine | Papuamine is cytotoxic against glioblastoma SF-295 cells (GI_50_ = 0.8 μM). | [[87](#_ENREF_87)] |
| 55 | Sponge | *Niphates olemda* | Mantehage, North Sulawesi, Indonesia at a depth of 10 m | niphateolide A | Niphateolide A inhibited the interaction with an IC_50_ value of 16 µM. | [[88](#_ENREF_88)] |
| 56 | Sponge | *Oceanapia sp.* | Manado bay, Sulawesi, Indonesia from a depth of 10-40 m | (7*E*,13*E*,15*Z*)-14,16-dibromohexadeca-7,13,15,trien-5-ynoic acid; (5*Z*,7*E*,13*E*,15*Z*)-6,14,16-tribromohexadeca-5,7,9,13,15-pentaenoic acid and (7*E*,9*E*,13*E*,15*Z*)-6,14,16-tribromohexadeca-7,9,13,15,tetraen-5-ynoic acid | The compounds showed weak cytotoxicity against KB cells (2+ at 10 µg/mL). (7*E*,9*E*,13*E*,15*Z*)-6,14,16-tribromohexadeca-7,9,13,15,tetraen-5-ynoic acid showed mild antimicrobial activity against Gram-positive bacteria. | [[89](#_ENREF_89)] |
| 57 | Sponge | *Pericharax heteroraphis* | Kapoposang Island, Indonesia at a depth of 44 ft | preclathridine-A; leucettamine-B and leucettamine-A | Antimicrobial against Gram (+) bacteria *Staphylococcus aureus* and fungus *Cladosporium herbarum* | [[90](#_ENREF_90)] |
| 58 | Sponge | *Petrosia (Petrosia) hoeksemai* | Menjangan Island (North of Bali Island) in Indonesia | manzamine A and xestomanzamine A | Cytotoxic | [[7](#_ENREF_7)] |
| 59 | Sponge | *Petrosia nigricans* | Pulau Baranglompo, Southwest Sulawesi, Indonesia at a depth of 27 ft (9 m) | nigricines 1-4 | Failed to demonstrate cytotoxic activity | [[91](#_ENREF_91)] |
| 60 | Sponge | *Petrosia* sp. | Coral reefs of North Sulawesi, Indonesia | sarasinoside S; sarasinosides A_1_; sarasinosides I_1_; sarasinosides J and melophlin C | Melophlin C inhibited protein tyrosine phosphatase 1B (PTP1B) activity with an IC_50_ value of 14.6 µM, while sarasinoside S; sarasinosides A_1_; sarasinosides I_1_ and sarasinosides J were not active at 15.2–16.0 µM. | [[92](#_ENREF_92)] |
| 61 | Sponge | *Phyllospongia sp.* | Makassar, Indonesia at a depth of 10-15 m | A series of scalarane class sesterterpenes A-H | All of the isolated compounds exhibited 30-95% inhibition of the growth of KB cells at 10 µg/mL. | [[93](#_ENREF_93)] |
| 62 | Sponge | *Plakinastrella sp.* | Manado, Sulawesi, Indonesia at the depth of ~3 to ~37 m | elenic acid and elenic acid (*R*-2,4-dimethyl-22-(*p*-hydroxyphenyl)-docos-3(*E*)-enoic acid | Cytotoxicity with IC_50_ of 5 µg/mL in P-388, A-549, and MEL-28 bioassays. | [[94](#_ENREF_94)] |
| 63 |  | *Plakortis cfr. lita* | Along the coasts of the Bunaken Island in the Bunaken Marine Park of Manado. | manadoperoxide B;  12-isomanadoperoxide B; manadoperoxidic acid B; short chain dicarboxylate monoester; semi-synthetic analogues of manadoperoxide B | 12-Isomanadoperoxide B was the most potent compound, with the closest IC_50_ value (11 µg/mL) to that of manadoperoxide B (IC_50_ 3 µg/mL). It was followed by the semi-synthetic derivative (IC_50_ 0.16 μg/mL) and the equipotent manadoperoxidic acid B and the manadoperoxide B analogues with low μg/mL level efficacy.  12-Isomanadoperoxide B appeared to display the largest selectivity index (SI, calculated by dividing the IC_50_ value against L6 cells to the IC_50_ value against the parasite) that is 10 times lower than that of manadoperoxide B. | [[95](#_ENREF_95)] |
|  |  |  | The coasts of the Bunaken Island in the Bunaken Marine Park of Manado, Indonesia. | plakofuranolactone | Plakofuranolactone can effectively inhibit AHL-induced bioluminescence. In addition, in an experiment on a wild-type strain of *P. aeruginosa*, the total protease activity (one of the virulence factors of *P. aeruginosa* which is under control of the LasI/R system) was decreased by treatment with plakofuranolactone. Therefore, plakofuranolactone can be regarded as a model scaffold to design a first generation of antivirulence drugs. | [[96](#_ENREF_96)] |
| 64 | Sponge | *Plakortis cfr. simplex* | Bunaken Marine Park of Manado, North Sulawesi, Indonesia. | manadoperoxides A - D | Manadoperoxides showed moderate antimalarial activity compared to that of plakortin and peroxyplakoric B3 ester, the latter differing from manadoperoxide B only by minor structural details | [[97](#_ENREF_97)] |
| 65 | Sponge | *Plakortis cfr. Simplex* | Bunaken Marine Park, Bunaken Island, Manado | manadoperoxides A - D | Manadoperoxides were assayed *in vitro* against D10 and W2 strains of *Plasmodium falciparum* and showed moderate antimalarial activity | [[97](#_ENREF_97)] |
| 66 | Sponge | *Plakortis nigra* | Celebes Sea, Northwest of Tanjung Batuanguf, Sulawesi, Indonesia at the depth of 7-40 m | plakorstatins 1 and plakorstatins 2 | Moderate cancer cell growth inhibition against the murine P388 lymphocytic leukemia cell line with ED_50_ values of 1.1 and 0.91 µg/ mL, respectively, for plakorstatins 1 and plakorstatins 2. | [[98](#_ENREF_98)] |
| 67 | Sponge | *Plukortis sp.* | Near Manado, Sulawesi, Indonesia | manadic acid A and B | Moderately active against various antitumor cell lines. | [[99](#_ENREF_99)] |
| 68 | Sponge | *Prianos sp.* | Manado Bay, Sulawesi, Indonesia at a depth of 120 ft | kauluamine | Kauluamine showed moderate immunosuppressive activity (MLR IC_50_ 1.57 µg/mL, LcV IC_50_ >25.0 µg/mL, LcV/MLR > 16) in the mixed lymphoma reaction and was inactive in cytotoxicity and antiviral assays. | [[100](#_ENREF_100)] |
| 69 | Sponge | *Pseudoceratina purpurea* | Watudodol, Banyuwangi at a depth of 10–30 m | aplysamine-2 and aeroplysinin-1 | Aplysamine-2 and aeroplysinin-1 showed growth inhibition of the mouse lymphoma cell line L5178Y with IC_50_ value of 1.7 µg/mL and 0.57 µg/mL respectively. | [[101](#_ENREF_101)] |
| 70 | Sponge | *Sidonops microspinosa* | A mangrove island located near Sulawesi, Indonesia at a depth of 33 ft | microspinosamide | Microspinosamide inhibited the cytopathic effect of HIV-1 infection in an XTT-*based in vitro* assay with an EC_50_ value of approximately 0.2 µg/mL. However, the compound was only cytoprotective over a modest concentration range because of cytotoxic effects toward the target cells (IC_50_ value of approximately 3.0 µg/mL). Microspinosamide represents the latest of a small group of cyclic depsipeptides from diverse marine sponges, which have been reported to exhibit anti-HIV activity. | [[102](#_ENREF_102)] |
| 71 | Sponge | *Spongia sp.* | Bunaken Marine Park, Sulawesi, Indonesia | 18-nor-3,17-dihydroxyspongia-3,13(16),14-trien-2-one;  18-nor-3,5,17-trihydroxyspongia-3,13(16),14-trien-2-one; spongiapyridine and 17-hydroxy-4-epi-spongialactone A | 18-Nor-3,5,17-trihydroxyspongia-3,13(16),14-trien-2-one modestly inhibited aromatase with an IC_50_ of 34 μM and induced quinone reductase 1 activity with a CD (the concentration needed to double the enzymatic response) of 11.2 μM. The remaining isolates were inactive. | [[103](#_ENREF_103)] |
| 72 | Red alga and symbiotic Sponge | *Sigmadocia symbiotica* | Biaro Island, Indonesia. | *cis*,*cis*-ceratospongamide and *trans*,*trans*-ceratospongamide | *Trans*,*trans*-ceratospongamide exhibits potent inhibition of sPLA2 expression in a cell-based model for antiinflammation (ED_50_ 32 µM), whereas the *cis*,*cis* isomer is inactive. *Trans*,*trans*-ceratospongamide was also shown to inhibit the expression of a human-sPLA2 promoter-based reporter by 90%. | [[104](#_ENREF_104)] |
| 73 | Sponge | *Siliquariaspongia mirabilis.* | Sulawesi Island, Indonesia at a depth of 43 m | celebesides A-C and theopapuamides B-D | Celebeside A neutralized HIV-1 in a single-round infectivity assay with an IC_50_ value of 1.9 ± 0.4 μg/mL while the nonphosphorylated analog celebeside C was inactive at concentrations as high as 50 μg/mL. Theopapuamides A-C showed cytotoxicity against human colon carcinoma (HCT-116) cells with IC_50_ values between 2.1 and 4.0 μg/ mL and exhibited strong antifungal activity against wildtype and amphotericin B-resistant strains of *Candida albicans* at loads of 1-5 μg/disk. | [[105](#_ENREF_105)] |
| 74 | Sponge | *Strepsichordaia aliena* | Turtle Bay, Sangakali, eastern Indonesia at a depth of 23 m | honu’enone; phyllofolactones H-K and phyllofenone C | Cytotoxic | [[106](#_ENREF_106)] |
|  |  |  | Turtle Bay, Sangakali, eastern Indonesia at a depth of 23 m | honulactones A-L | Honulactones A-D exhibit cytotoxycity against P-388, A-549, HT-29, and MEL-28 (IC_50_ 1 µg/mL). | [[107](#_ENREF_107)] |
| 75 | Sponge | *Strepsichordaia lendenfeldi* | Indonesia at a depth of 20-30 ft | strepsiamide A-C | Strepsiamide A and C were active against Hela cells and L5178Y. Strepsiamide B was active against L5178Y. | [[108](#_ENREF_108)] |
| 76 | Sponge | *Stylissa carteri* | Sulawesi (Pulau Baranglompo) and Ambon (Seram Tanjung Sial), Indonesia at a depth of 3-23 m | debromostevensine; debromohymenin; stevensin; hymenin;  (*Z*)-debromohymenialdisine;  (*Z*)-hymenialdisine;  (*Z*)-3-bromohymenialdisine;  (*E*)-debromohymenialdisine;  (*E*)-hymenialdisine; spongiacidin A [=(*E*)-3-bromohymenialdisine] and oroidin | (*Z*)-debromohymenialdisine and (*Z*)- hymenialdisine proved to be active against MONOMAC-6 cells. The IC_50_ values of these two compounds were 2.4 and 0.2 µg/mL, respectively. | [[109](#_ENREF_109)] |
| 77 | Sponge | *Stylissa massa* | Papua island, Indonesia | dispacamide E; ethyl 3,4-dibromo-1*H*-pyrrole-2-carboxylate;  4-bromopyrrole-3-carboxamide;  3,4-dibromopyrrole-2-carboxamide; (2)-longamide B; (2)-longamide B methyl ester; (2)-longamide B ethyl ester; hanishin; aldisine;  2,3-dibromoaldisine;  2-bromoaldisine; 3-bromaldisine;  (2)-mukanadin C; (2)-longamide; latonduine A; (2)-dibromophakellin; (2)-monobromoisophakellin;  (2)-dibromocantharelline;  (2)-hymenine; spongiacidin B;  (10*Z*)-debromohymenialdisine;  (10*Z*)-hymenialdisine;  (10*Z*)-3-bromohymenialdisine;  (10*E*)-hymenialdisine; latonduine B ethyl ester; 3-debromolatonduine A; stevensine and 12-*N*-methyl stevensine | (10*Z*)-Debromohymenialdisine was the most potent derivative with an IC_50_ value of 6.33 µM. Dispacamide E, the brominated aldisines, (2)-mukanadin C and (2)-longamide, revealed significant inhibitory activities against GSK-3, DYRK1A and CK-1 with IC_50_ values ranging between 0.6 and 6.4 µM. All isolated compounds were assayed for their antiproliferative and protein kinase inhibitory activities Some of the tested compounds were shown to possess significant antiproliferative activities with IC_50_ values ranging between 6.33 and 28.28 µM. Dispacamide E; 2,3-dibromoaldisine;  2-bromoaldisine; 3-bromaldisine;  (2)-mukanadin C and (2)-Longamide, which were inactive in the antiproliferative assay, revealed significant inhibitory activities against GSK-3, DYRK1A and CK-1 with IC_50_ values ranging between 0.6 and 6.4 µM.  (2)-hymenine;  (10*Z*)-debromohymenialdisine;  (10*Z*)-hymenialdisine;  (10*Z*)-3-bromohymenialdisine;  (10*E*)-hymenialdisine exhibited potent inhibitory activity against PfGSK-3 with IC_50_ values in the nanomolar range which may strengthen their role as potential antimalarial candidates. | [[110](#_ENREF_110)] |
| 78 | Sponge | *Stylissa flabelliformis* | Papua island, Indonesia | 3,4-dibromopyrrole-2-carboxamide; 2-bromoaldisine; (2)-longamide; latonduine A; (2)-dibromoisophakellin;  (10*Z*)-hymenialdisine;  (10*E*)-hymenialdisine; latonduine B ethyl ester; 3-debromolatonduine A; stevensine and 12-*N*-methyl stevensine | All isolated compounds were assayed for their antiproliferative and protein kinase inhibitory activities. Some of the tested compounds were shown to possess significant antiproliferative activities with IC_50_ values ranging between 6.33 and 28.28 µM. | [[110](#_ENREF_110)] |
| 79 | Sponge | *Stylissa sp.* | Biak, Indonesia | stylissamide X | Stylissamide X showed inhibitory activity against migration of HeLa cells in the ranges of 0.1–10 µM, while the cell viability was maintained more than 75% up to 10 µM concentration of stylissamide X | [[111](#_ENREF_111)] |
|  |  |  | Derawan Island, Indonesia | 12-*N*-methyl stevensin; 12-*N*-methyl-2-debromostevensine;  3-debromolatonduine B methyl ester; 3- debromolatonduine A;  *Z*-hymenialdisine;  *Z*-debromohymenialdisine; stevensine; 2-debromostevensine;  3-bromoaldizine; 3,4-dibromopyrrole-2-carbamide; latonduine A and latonduine B methyl ester | 12-*N*-Methyl stevensine; *Z*-hymenialdisine; *Z*-de-bromohymenialdisine; and latonduine A showed significant *in vit*ro activity with EC_50_ values of 3.5, 1.8, 2.1, and 9.0 µg/mL, respectively. | [[112](#_ENREF_112)] |
| 80 | Sponge | *Theonella cupola* | North of Manado Bay, Indonesia at the depth of 64 m | cupolamide A | Cupolamide A is active against P388 murine leukemia cells with an IC_50_ 7.5 µg/mL but not against thrombin. | [[113](#_ENREF_113)] |
| 81 | Sponge | *Theonella sp.* | Indonesia | kumusine | Kumusine showed moderate immunosuppressive activity (MLR IC_50_ 0.195 µg/mL, LcV IC_50_ 5.0 µg/mL, potency >256), and cytotoxicity against P388 (IC_50_ 5.0 µg/mL), A549 (IC_50_ 2.5 µg/mL), HT29 (IC_50_ 5.0 µg/mL), and CV1 (IC_50_ 2.5 µg/mL). | [[114](#_ENREF_114)] |
| 82 | Sponge | *Theonella swinhoei* | Baranglompo Island, Indonesia | barangamide A - D; and theonellapeptolide Id, IId, IIe, Ia, and Ie | Theonellapeptolide IId showed the strongest immunosuppressive. Theonellapeptolides Ia-Ie have been reported to be cytotoxic (IC_50_ 1.3±2.4 mg/mL) against L1210 tumor cells. Barangamide A showed no activity while theonellapeptolide Id, IId, Ia were active in MLR assay. | [[115](#_ENREF_115)] |
|  |  |  | Along the coast of the Bunaken Island in the Bunaken Marine Park, North Sulawesi, Indonesia at the depth of 20-50 m | aurantoic acid; dehydroconicasterol; aurantoside G; conicasterol and theonellasterol | All of the isolated compounds were tested for *in vitro* cytotoxic activity against three cell lines (C6, glioma; HeLa, epithelial carcinoma; H9c2, cardiac myoblast), and they exhibited no significant inhibition of the cell growth (IC_50_ > 70 μM). | [[116](#_ENREF_116)] |
|  |  |  | Baranglompo Island, Indonesia | theonellapeptolides Id; theonellapeptolides IId and barangamide A | In a preliminary assay no cytotoxicity has been observed with barangamide A, while theonellapeptolides Ia-Ie have been reported to be cytotoxic against L1210 (IC_50_ 1.3-2.4 µg/mL) | [[117](#_ENREF_117)] |
|  |  |  | Bunaken Marine Park of Manado, North Sualwesi, Indonesia | theonellapeptolide Id; sulfinyltheonellapeptolide and theonellapeptolide If | The three theonellapeptolides have been evaluated for their antiproliferative activity against HepG2 cells, a hepatic carcinoma cell line. All tested compounds showed moderate antiproliferative activity against HepG2 cells. At the dose 10 μM, the proliferation rate of HepG2 cells was significantly reduced by theonallapeptolide Id, sulfinyltheonellapeptolide and theonellapeptolide If to about 50%, 30% and 50%, respectively. Only theonellapeptolide Id was able to significantly reduce the proliferation at 1 μM. | [[118](#_ENREF_118)] |
| 83 | Sponge | *Theonella cf. swinhoei* | Menjangan Island, North of Bali Island, Indonesia | theonellapeptolide Id; theonellapeptolide Ie; theonellapeptolide IId and swinholide A | Swinholide A possessing potent inhibitory activity against thrombin, trypsin and plasmin, and other acyclic and cyclic polypeptides ranging from cyclic depsipeptide to large bicyclic peptides. Biological activity of theonellapeptolides (Ia-e and IId) are cytotoxicity, ion-transport inhibitory activity for Na+ and K+ ions, and Na+, K+-ATPase inhibitory activity. Theonellapeptolides Ia and Ie have been reported to be cytotoxic (IC_50_ 1.3±2.4 µg/mL) against L1210 tumor cells. The observed immunosuppressive activity of theonellapeptolides may largely originate from their cytotoxic effect | [[7](#_ENREF_7)] |
| 84 | Sponge | *Xestospongia cf.vansoesti* | Coral reef along Lembeh Strait off Bitung, Sulawesi Island, Indonesia at the depth of 10 to ~20 m | bitungolides A-F | Antitumor and inhibit dual-specificity phosphatase VHR. Bitungolides showed weak activity against dual-specificity phosphatase (VHR), while no activity was observed against serine/threonine phosphatase (PP1 and PP2A) or tyrosine phosphatase (PTP-S2). | [[119](#_ENREF_119)] |
| 85 | Sponge | *Xestospongia exigua* | North Sulawesi, Indonesia at a depth of 10 m | salsolinol; norsalsolinol; *cis*-4-hydroxysalsolinol and *trans*-4-hydroxysalsolinol | Salsolinol and norsalsolinol inhibited the chymotrypsin-like activity of the proteasome with IC_50_ values of 50 and 32 µg/mL, respectively, but *cis*-4-hydroxysalsolinol and *trans*-4-hydroxysalsolinol showed no inhibitory effect even at 100 µg/mL. Salsolinol showed cytoxtoxic activity against HeLa, L1210, FL, KB, and A549 with value of IC_50_ values 17, 8, 13, 20, and 27 µg/mL. Norsalsolinol was not tested. | [[120](#_ENREF_120)] |
| 86 | Sponge | *Xestospongia sp.* | Pecaron Bay Situbondo, East Java, Indonesia at the depth of 5-20 m | - | The specimen showed cytotoxic activity against level on HT-29 (124.45 ± 3.12 µg/mL) T47D (98.23 ± 3.21µg/mL) and Casky (68.98 ± 2.34 µg/mL) and moderate antioxidant activity with a IC_50_ 89.17 µg/mL. | [[3](#_ENREF_3)] |
|  |  |  | Coral reef habitat at Sangalaki, Indonesia at a depth of approximately 20 m | xestosaprols F-M | The compounds inhibited the aspartic protease BACE1 at moderate levels in a dose-dependent manner. Xestosaprols H displayed the lowest IC_50_ value at 82 µM. Xestosaprols I displayed the highest IC_50_ value at 163 µM. | [[121](#_ENREF_121)] |
|  |  |  | Near Turtle Bay, Sangalaki, Indonesia | xestosaprol D; xestosaprol E; adocianoquinones A and adocianoquinones B | The compound showed antimicrobial activity against multiple-resistant *Staphylococcus aureus*. The cytotoxins adociaquinones A and B, with IC_50_ values of 9.47 and 4.23 µg/mL, respectively.  Xestosaprol D and xestosaprol E did not display an appreciable antimicrobial (VRE, *E. coli*, MRSA, SA) or cytotoxic effect (SKOV- 3 cells, IC_50_ > 50 µg/mL) and did not significantly activate or inhibit the oncogene Protein Kinase C (PKCd). Adocianoquinones B has been shown to inhibit topoisomerase II in CHO cell line xrs-6. Xestosaprol D showed weakly inhibit the aspartic protease BACE-1 with an IC_50_ value of approximately 30 µg/mL. | [[122](#_ENREF_122)] |
|  |  |  | North Sulawesi (Bunaken, and other islands/reefs near manado), Indonesia | xestoadociaquinones A, B;  14-carboxyxestoquinol sulfate; xestoadociaminals A-D; adociaquinone A, B; secoadociaquinone A,B; 15-chloro-14-hydroxyxestoquinone; 15-chloro-15-hydroxyxestoquinone and xestoquinol sulfate | Adociaquinone B and secoadociaquinone B revealed a modest but selective inhibitory activity towards CDK9/cyclin T (IC_50_: 3 μM) and CDK5/p25 (IC_50_: 6 μM), respectively. Xestoquinol sulfate showed moderate antibacterial activity with an IC_50_  value 125 µM against *Staphylococcus aureus* | [[123](#_ENREF_123)] |
|  |  |  | Pecaron Bay Situbondo, East Java, Indonesia at the depth of 5-20 m dept | - | Showed cytotoxic activity against level on HT-29 (33.54±0.88 µg/mL) T47D(34.38±0.98 µg/mL) and Casky (80.60±2.34 µg/mL. Showed antioxidant activity with a value of IC_50_ 277.75 µg/mL | [[3](#_ENREF_3)] |
|  |  |  | Jakarta at a depth of 5 m | aaptamine; isoaaptamine; demethyl(oxy)aaptamine;  8,9,9-trimethoxy-9*H*-benzo[*d,e*][1,6]-naphthyridine;  2-ethyl-11-methoxy-3-methyl-3*H*-[1,6]naphthyridino[6,5,4-*def*]quinoxaline;  11-methoxy-3*H*-[1,6]naphthyridino[6,5,4-*def*]quinoxalin-2-ol; Novel benzo[*de*][1,6]naphthyridine moiety.derivatives A and B | Aaptamine; isoaaptamine; demethyl(oxy)aaptamine and the novel benzo[*de*][1,6]naphthyridine moiety. Derivative B showed moderate antibacterial activity, activity on the ichthyopathogenic strain *V. anguillarum* being the most significative. Aaptamine and isoaaptamine exhibited antifungal activity towards *C. tropicalis*. Aaptamine; isoaaptamine and demethyl(oxy)aaptamine showed a significant cytotoxic activity on KB cells. The new dimethylketal, 8,9,9-trimethoxy-9*H*-benzo[*de*][1,6]-naphthyridine, also displayed interesting cytotoxic activity. | [[124](#_ENREF_124)] |
| 87 | Sponge | *Xestospongia testudinaria* | In the south west of Kaimana, West Papua, Indonesia at a depth of 10 m | kaimanol and saringosterol | Kaimanol and saringosterol showed antiplasmodial activity with IC_50_ values of 359 and 0.250 nM, respectively. | [[125](#_ENREF_125)] |
|  |  |  | Badi Island, South Sulawesi Province, Indonesia at depths of 10-18 m | methyl 18-bromo-(13*E*,17*E*)-octadeca-13,17-diene-5,7,15-triynoate and methyl 20-bromoeicosa-11*E*,15*E*,19*E*-triene-7,9,17-triynoate | Methyl 18-bromo-(13*E*,17*E*)-octadeca-13,17-diene-5,7,15-triynoate showed higher inhibition zones than compound methyl 20-bromoeicosa-11*E*,15*E*,19*E*-triene-7,9,17-triynoate by the agar diffusion method. Methyl 18-bromo-(13*E*,17*E*)-octadeca-13,17-diene-5,7,15-triynoate can inhibit *Vibrio harveyi* with the highest inhibition zone (7.86 mm), followed *V. alginolyticus* (7.56 mm), and *V. parahaemolyticus* (6.50 mm). | [[126](#_ENREF_126)] |
|  |  |  | Mengangan Island, Bali, Indonesia | aspergillitine and aspergione A-G | Aspergillitine displayed moderate antibacterial activity against *Bacillus subtilis* while it was inactive against *Escherichia coli* and *Saccharomyces cerevisiae.* In the same assay systems, aspergiones C and E were inactive. | [[127](#_ENREF_127)] |
|  |  |  | Mengangan Island, Bali Sea, Indonesia. | xestodecalactones A; xestodecalactones B and xestodecalactones C | Xestodecalactones B showed an active activity against *C. Albicans.* All compounds were inactive against *B. Subtilis, S. aureus, E. coli.* | [[128](#_ENREF_128)] |

**Supplementary Table 2. Chemical and pharmacological data of Indonesian Ascidian**

| **No** | **Type** | **Species** | **Location** | **Constituen** | **Bioactivity** | **Ref.** |
| --- | --- | --- | --- | --- | --- | --- |
| 1. | Ascidian | *Botryllus* sp. | at Barrang Caddi (5° 4.647 S,  119° 19.070 E at -3 to -15 m), Indonesia | cadiolides A and B, rubrolide A | The cadiolides were found to be inactive against the human colon tumor cell line HCT-116, and their biological role is not known. | [[129](#_ENREF_129)] |
| 2. | Ascidian | *Diazona* sp. | - First sample (ORMA034977, 38 g) : near Manado (0^o^ 32’ 39” S, 130^o^ 41’ 53” E) - Second Sample (ORMA043097, 137 g) : at Raja Ampat (1^o^ 37’ 44” N, 124^o^ 45’ 56” E) | diazonamides A, C, D, E | Diazonamides A,C,D, and E showed moderate cytotoxicity (GI_50_= 0.006 µM -9.0 µM) against three human tumor cell lines, including lung (A549), colon (HT29), and breast (MDA-MB-231). | [[130](#_ENREF_130)] |
| 3. | Ascidian | *Eusynstyela latericius* | At 10 m, Ujung Pandang, Indonesia | styelsamine A-D | Styelsamine A-D exhibited mild cytotoxicity toward the human colon tumor cell line HCT-116, with IC_50_ values of 33, 89, 2.6, and 1.6 µM, respectively | [[131](#_ENREF_131)] |
| 4. | Ascidian | *Leptoclinides dubius* | Lembeh Strait, North Sulawesi,  Indonesia | leptoclinidamide, (-)-leptoclinidamide B, *C*2-*α*-D-mannosylpyranosyl-L-tryptophan, *N*-acetyl derivative | No biological activities have been reported for *C*2-*α*-D-mannosylpyranosyl-L-tryptophan. Leptoclinidamide, (-)-leptoclinidamide B, *C*2-*α*-D-mannosylpyranosyl-L-tryptophan, and *N*-acetyl derivative were tested for their cytotoxicity against two human cancer cell lines (colon adenocarcinoma HCT-15 and T-cell leukemia Jurkat cells). None of the four compounds displayed activity against these cell lines at 30 µM. Leptoclinidamide, (-)-leptoclinidamide B, and *C*2-*α*-D-mannosylpyranosyl-L-tryptophan showed no antimicrobial activity. | [[132](#_ENREF_132)] |
| 5. | Ascidian | *Lissoclinum* cf. *badium* | At 7 to 19 m off the coral reef in Manado, Indonesia.  Collected in March (wet season) in 2006 but were not obtained from  the same organism collected in September (dry season) in 2003, 2004, and 2005 at the same sampling site. | lissoclibadins 1-7, lissoclinotoxins E and F, 3,4-dimethoxy-6-(2′-*N,N*-dimethylaminoethyl)-5-(methylthio)benzotrithiane,  and *N,N*-dimethyl-5-(methylthio)varacin | Lissoclibadins 4-7 inhibited the colony formation of Chinese hamster V79 cells with EC_50_ values of 0.71, 0.06, 0.06, and 0.17 µM, respectively and showed weak antibacterial activity against *Staphylococcus aureus* and *Escherichia coli*. Lissoclibadins 5 and 6 showed modest antiyeast activity against *Saccharomyces cere*v*isiae.* | [[133](#_ENREF_133)] |
|  |  |  | At 5 to 21 m off the coral reef in Manado, Indonesia | Lissoclibadins 1,3,4,7, and8-14, lissoclinotoxins E and F | All compounds inhibited the colony formation of Chinese hamster V79 cells and cell proliferation of murine leukimia L1210 cells. Lissoclibadin 12 was not active at 20.0 µM (9.0 µg/mL) and 11 also showed a reduced activity. Lissoclibadin 10 showed similar magnitudes of antiproliferative activity (data not shown) against human breast MDA-MB-231 and large-cell lung NCI-H460 cell lines to Lissoclibadin 1. | [[134](#_ENREF_134)] |
| 6. | Ascidian | *Polycarpa aurata* | In the Lembeh Strait, North Sulawesi, Indonesia | *N*,*N*-didesmethylgrossularine-1 (DDMG-1) | Inhibit TNF-*α* production in lipopolysaccharide-stimulated murine macrophage-like RAW264.7 cells. There is an inhibitory effect on the production of IL-8 which is produced in CD14+-THP-1 cells stimulated by LPS. | [[135](#_ENREF_135)] |
|  |  |  |  | polycarpaurines A,B,C, polycarpine,  2-(4-methoxyphenyl)-*N*-methyl-2-oxoacetamide, *N*-(4-methoxybenzoyl)-*N*′-methylguanidine, *N,N*-didesmethylgrossularine-1, and  *p*-methoxybenzoic acid | Polycarpaurines A,C, polycarpine, and *N*,*N*-didesmethylgrossularine-1 inhibited colony formation of Chinese hamster V79 cells with EC_50_ values of 6.8, 8.6, 3.8, and 10 µM respectively. Polycarpaurines B and *N*-(4-methoxybenzoyl)-*N*′-methylguanidine showed modest activity against V79 cells (EC_50_>10 µM) | [[136](#_ENREF_136)] |
| 7 | Ascidian | *Lissoclinum cf badium* | At ~7 to ~19 m off the coral reef in Manado, Indonesia.  Collected in March (wet season) in 2006 but were not obtained from the same organism collected in September (dry season) in 2003, 2004, and 2005 at the same sampling site. | lissoclibadins 1,3,4,7,8, and 14 | Lissoclibadins 1,3,4,7,8, and 14 showed cytotoxic activity against HCT-15 with IC_50_ values of 4 ± 1.8, 13.2 ± 1.4, 17.2 ± 5.2, 15.7 ±1.2, 4.9 ± 1.9, 4.2 ± 2.4, and 19.7 ± 5.7 µM, respectively.  Lissoclibadins 1,3,4,7,8,and 14 showed cytotoxic activity against HeLa-S3 with IC_50_ values of 6.3 ± 1.4, 16.0 ± 1.8, 17.8 ± 1.8, 14.2 ± 1.3, 6.3 ± 0.5, 5.9 ± 1.6, and 43.1 ± 6.5 µM, respectively.  Lissoclibadins 1 ,8, and 14 showed cytotoxic activity against MCF-7 with IC_50_ values of 7.6 ± 4.0, 11.8 ± 3.1, and 6.4 ± 2.7 µM, respectively.  Lissoclibadins 1 ,8, and 14 showed cytotoxic activity against NCI-H28 with IC_50_ values of 7.1 ± 2.3, 7.1 ± 1.0, and 6.4 ± 1.6 µM, respectively. | [[137](#_ENREF_137)] |
| 8 | Ascidian | *Lissoclinum bistratum* | Raja Ampat Islands (Papua Bar, Indonesia) (00°33.353’S/130° 41.156’E) at depths ranging between 1 and 8 m | bistratamide M and bistratamide N | Bistratamide M showed cytotoxic activity against MDA-MB-231, HT-29, NSLC A-549, and PSN1 with GI_50_ value of 18, 16, 9.1, and 9.8 µM, respectively.  Bistratamide N showed cytotoxic activity against MDA-MB-231, HT-29, NSLC A-549, and PSN1 with GI_50_ value of 0.2, 0.3, 0.2, and 0.2 µM, respectively. | [[138](#_ENREF_138)] |
| 9 | Ascidian | *Polycarpa aurata* | The coast of Siladen (Indonesia, 1° 37’41’’ N 124° 48’01’’ E) | polyaurines A and polyaurines B | Both compounds were not active and showed IC_50_ values higher than 100 µM against both mammalian cells and larval stage (schistosomula) of *Schistosoma mansoni*. | [[139](#_ENREF_139)] |
| 10 | Ascidian | *Polycarpa aurata* | Barrang Caddi waters, Spermonde Archipelago, South Sulawesi, Indonesia | Crude extract | *P. aurata* crude extract were shown to have antibacterial activity against *Bacillus cereus,* MRSA, *Escherichia coli, Salmonella typhi, Candida albicans, Trichophyton rubrum* with inhibition zone diameters of 6.65 ± 0.49, 11.5 ± 0.75, 12.60 ± 1.4, 7.10 ± 0.84, 0, and 0 mm, respectively.  *P. aurata* crude extract were reported to have antioxidants activity with an IC_50_ value of 534.60 ppm.  *P. aurata* crude extract showed cytotoxic activity against P-388 murine leukemia cancer cels with an IC_50_ value of 80 µg/ml. | [[140](#_ENREF_140)] |

**Supplementary Table 3. Chemical and pharmacological data of Indonesian Gorgonian**

| **No** | **Type** | **Species** | **Location** | **Constituens** | **Bioactivity** | **References** |
| --- | --- | --- | --- | --- | --- | --- |
| 1 | Gorgonian | *Briareum* sp. | at 10-15 m  depths off Togian Island (Indonesia) | 2,9-diacetyl-2-debutyrylstecholide H, 13-dehydroxystecholide J, 2*β*-acetoxy-2-(debutyryloxy) stecholide E acetate, stecholides I-M, stecholide A acetate, and stecholide C acetate | Stecholides L showed mild cytotoxic activity against tumor cells P-388 (IC_50_ 10 µg/mL), A-549 (IC_50_ 2.5 µg/mL), HT-29 (IC_50_ 5 µg/mL), and MEL-28 (IC_50_ 5 µg/mL).  2,9-Diacetyl-2-debutyrylstecholide H, 13-dehydroxystecholide J, The semisynthetic 2*β*-acetoxy-2-(debutyryloxy) stecholide E acetate , stecholides I, J, K, and C were devoid of cytotoxic activity against tumor cells P-388, A-549, HT-29, and MEL-28 (IC_50_ >10).  The semisynthetic 2*β*-acetoxy-2-(debutyryloxy) stecholide E acetate showed significant activity against the growth of P-388 cells (EC_50_ 1.59 *µ*g/mL). | [[141](#_ENREF_141)] |
| 2 | Gorgonian | *Isis hippuris* | At a depth of 25 m in Sulawesi, Indonesia | hippuristanol, 3-acetyl-2*α*-hydroxy-22-*epi*-hippuristanol, 22-*epi-*hippuristanol, 3-acetyl-22-*epi-*hippuristanol, 11-dehydroxy-22-*epi-*hippuristanol, 11-dehydroxy-22-*epi-*hippuristan-3-one, orthohippurinsterone A, orthohippurinsterol A, orthohippurinsterol B, hippuristerone B, hippuristerol B, hippuristerone A, hippuristerol A, hippuristerol C, hippuristerone C, hippuristerol D, and hippuristerone D | Hippuristanol and 22-*epi-*hippuristanol were the most active compounds tested as anticancer against P-388, A549, HT29, and MEL28 cell line with an IC_50_ value of 0.1 µg/mL.  3-Acetyl-22-*epi-*hippuristanol showed anticancer activity against P-388, A549, HT29, and MEL28 cell line with IC_50_ values of 1, 0.125, 0.5, and 0.125 µg/mL, respectively.  Hippuristerol B showed anticancer activity against P-388, A549, HT29, and MEL28 cell line with an IC_50_ value of 1.25 µg/mL.  The rest compounds indicated moderate activities against P-388, A549, HT29, and MEL28 cell line with IC_50_ values of 2.5-10 µg/mL. | [[142](#_ENREF_142)] |
| 3. | Gorgonian | *Junceella fragilis* | at Halmahera Island (Indonesia)  (1° 41.550¢ N, 127° 32.156¢ E) at a depth of 27-33 m | (-)-4-deacetyljunceellolide D, (+)-11*R*,20*R-*epoxyjunceellolide D, (-)-11*R*,20*R*-epoxy-4-deacetyljunceellolide D, (-)-11*R*,20*R*-epoxy-4-deacetoxyjunceellolide D, (+)-junceellolide A, (-)-junceellolide A], (-)-junceellolide D, (-)-junceellin, and (-)-praelolide | All compounds showed no cytotoxic activity against several tumor cells (P-388, A-549, HT-29, MEL-28). | [[143](#_ENREF_143)] |

**Supplementary Table 4. Chemical and pharmacological data of Indonesian marine algae**

| **No** | **Species** | **Location** | **Constituent/ Extracts** | **Bioactivity** | **Ref** |
| --- | --- | --- | --- | --- | --- |
| 1 | *Acanthophora muscoides* | North West Lombok area, Mentigi beach (8°24ʹ11.7396″S, 116°4ʹ1.9056″E), West Nusa Tenggara Province, Indonesia | Ethanol extracts | Cytotoxicity (HeLa cells, IC_50_ = 180 ± 14 µg/mL, against HeLa cells in 72 hours incubation). | [[144](#_ENREF_144)] |
| 2 | *Acanthophora muscoides* | North West Lombok coastal area, West Nusa Tenggara province, Indonesia | Ethanol extracts | Antiproliferative activity; cell density was suppressed to 29×10^4^ cells mLG1 compared to untreated cells (78×10^4^ cells mLG1 ). | [[145](#_ENREF_145)] |
| 3 | *Acanthophora Spicifera* | North West Lombok area, Mentigi beach (8°24ʹ11.7396″S, 116°4ʹ1.9056″E), West Nusa Tenggara Province, Indonesia | Ethanol extracts | Cytotoxicity (HeLa cells, IC_50_ = 190 ± 24 µg/mL, 72 h incubation) against HeLa cells in 72 hours incubation. | [[144](#_ENREF_144)] |
| 4 | *Acrocystis nana* | Southern coast of Java Island, Gunung Kidul Regency, Yogyakarta, Indonesia | Crude lectins | The crude lectins had not shown potent cytotoxic effects on MCF-7 and HeLa cells with an average inhibition of 47.68% against HeLa cells and giving lower inhibition against MCF-7 cells. | [[146](#_ENREF_146)] |
| 5 | *Eucheuma cottonii* | East Lombok beach, Nusa Tenggara Barat, Indonesia | Chloroform and ethyl acetate extracts | Cytotoxic (HeLa cells, activity against cervical HeLa cells with IC_50_ up to 4.82 and 4.34 μg /mL, respectively). | [[147](#_ENREF_147)] |
| 6 | *Eucheuma cottonii* | Lemuutan Island waters, West Kalimantan, Indonesia | Ethanol extract | The extraxt showed moderate antioxidant activity (DPPH, IC_50_ of 127.75 ppm), and anti-inflammatory (RBC, anti-inflammatory activity with the concentration of 219.83 ppm). | [[148](#_ENREF_148)] |
| 7 | *Eucheuma* sp. | Binuangeun, a place at the southern coast of West Java (6⁰50'4"S -105⁰52'49"E), Indonesia at about 4 m depth | Water extract | Antioxidant (FRAP, IC_50_ 19.08±0.66 AA mg/g), with tyrosinase inhibition (1000 μg/mL, enzymes of 41.36±3.80%) % (1,000 ppm). | [[149](#_ENREF_149)] |
| 8 | *Gelidium latifolium* | Lombok coast Indonesia | Ethanol extracts | Cytotoxicity in HeLa cells with EC_50_ of 144.42 ± 12.08 µg/mL. | [[144](#_ENREF_144)] |
| 9 | *Gracilaria edulis* | Indonesia | Ethyl acetate extracts and hexane-ethyl acetate gradient fractions | Antibacterial (antimicrobial agent fraction EtOAc:hexane (1:1), *Vibrio fluvialis, Aeromonas hydrophyla*, MIC 1.25, 0.625 μg /mL, respectively). | [[150](#_ENREF_150)] |
| 10 | *Gymnogongrus* sp | Hutumuri and Mahia hamlet of Urimesing village of Ambon city, Indonesia | Hexane extracts | Antibacterial activity against *Escherichia coli*, *Salmonella thypi* and *Bacillus subtilis* with inhibition diameter of 42.33, 37.50 and 20.83 mm, respectively. | [[151](#_ENREF_151)] |
| 11 | *Halimeda cylindracea* | Gulf of Boni, South Sulawesi, Indonesia | Ethyl acetate, acetone extract, and acetone extract | Cytotoxicity toward *Arthemia salina* Leach, with LC_50_ value 281.84 µg/mL.  Antibacterial activity against *S. aureus* and *E. coli.* | [[152](#_ENREF_152)]  [[152](#_ENREF_152)] |
| 12 | *Halimeda macrobola* | Selayar and Kapoposang Island, Indonesia. | Protein fraction | Antioxidant activity (DPPH) with IC_50_ values of 0.110 mg/mL).  Anticancer activity by Brine Shrimp Lethality Test (BSLT) method showed LC_50_ values of 0.29 µg/mL and IC_50_ value of 53.80 µg/mL. | [[153](#_ENREF_153)] |
| 13 | *Halimeda macroloba* | Indonesia | Methanol extract | Antibacterial activity against *S. aureus* at concentration 20%, with inhibition diameter 10.83 ± 1.07 mm. | [[154](#_ENREF_154)] |
| 14 | *Halymenia* sp. | Binuangeun, a place at the southern coast of West Java (6⁰50'4"S -105⁰52'49"E), Indonesia at about 4 m depth | Water extract | Antioxidant (FRAP) IC_50_ 19.40 ± 0.37 AA mg/g), with tyrosinase inhibition (1000 μg/mL, enzymes of 40.09±2.01%) (1,000 ppm). | [[149](#_ENREF_149)] |
| 15 | *Hypnea asperi* | Indonesia | Methanol extract | Antibacterial activity against *S. aureus,* at concentration 20%, with inhibition diameter 11.92 ± 2.45 mm. | [[154](#_ENREF_154)] |
| 16 | *Padina australis* | Pramuka Island, District of Seribu Island, Indonesia | Methanol extract | Antioxidant activity (DPPH) IC_50_ 267.1 ppm) | [[155](#_ENREF_155)] |
| 17 | *Padina australis* | Atep Oki Village, Lembean Timur Minahasa | Ethanol extract | The extract showed antibacterial activity against *S. aureus*, *S. mutans* and *E. coli* with inhibition diameter of 11.6, 11.8, and 11.3, respectively. | [[156](#_ENREF_156)] |
| 18 | *Padina* sp. | Latuhalat Village, Ambon Island, Indonesia | Methanol extract | Strong antibacterial activity against *E. coli* with inhibition diameter of 26.5 mm and moderate antibacterial activity against *Salmonella thypimirium* with inhibition diameter of 19.0 mm. | [[157](#_ENREF_157)] |
| 19 | *Padina tetrastromatica* | Jumiang beach, Pamekasan (a non-oil extraction site), Madura and Camplong beach, Kabupaten Sampang (an oil extraction site), Madura Island, Indonesia | Crude extracts (methanol extract), hexane, ethyl acetate, and methanol residue fractions | Antioxidant (DPPH, IC_50_ 25.25 ± 5.15 mg/mL), antidiabetic (*α*-glucosidase inhibition, 249.12 ± 1.77 mg/mL), cytotoxic (A549, IC_50_ activities with IC_50_ 25.25 ± 5.15, 249.12 ± 1.77 and 70.56 ± 2.56 µg/mL), respectively. | [[158](#_ENREF_158)] |
| 20 | *Sargassum crassifolium* | North West Lombok area, Mentigi beach (8°24ʹ11.7396″S, 116°4ʹ1.9056″E), West Nusa Tenggara Province, Indonesia | Ethanol extracts | Reduced nuclear DNA damage induced by UV-B radiation. | [[144](#_ENREF_144)] |
| 21 | *Sargassum cristaefolium* | West coastal area of Lombok (8°29′54.251″ S and 116°4′36.664″ E), Indonesia | Ethanol extract | Skin protection (*in vivo*) potentially by attenuation of inflammatory cytokines.  Antibacterial activity against *Staphylococcus aureus*, (MIC = 1.302 µg/mL). | [[159](#_ENREF_159)] |
| 22 | *Sargassum cristaefolium* | Batu Layar Coast of Lombok island (8°29′54.251″ S and 116°4′36.664″ E), Indonesia | Ethanol extract | Antioxidant (DPPH, IC_50_ 737.30 ± 23.46 µg/mL). | [[160](#_ENREF_160)] |
| 23 | *Sargassum cristaefolium* | Lendang luar beach, West Lombok, Indonesia | Ethanol extract | Antioxidant (DPPH, IC_50_ 202.7 ± 1.22 µg/mL). | [[161](#_ENREF_161)] |
| 24 | *Sargassum polycystum* | North West Lombok area, Mentigi beach (8°24ʹ11.7396″S, 116°4ʹ1.9056″E), West Nusa Tenggara Province, Indonesia | Ethanol extracts | Reduced nuclear DNA damage induced by UV-B radiation. | [[144](#_ENREF_144)] |
| 25 | *Sargassum polycystum* | Batu Layar Coast of Lombok island (8°29′54.251″ S and 116°4′36.664″ E), Indonesia | Ethanol extract | Antioxidant (DPPH, IC_50_ 804.30 ± 30.82 µg/mL). | [[160](#_ENREF_160)] |
| 26 | *Sargassum polycystum* | Sepanjang Coast in Gunungkidul,Yogyakarta,Indonesia | Ethanol extract | Antibacterial activity against *E. coli* and *S. typhi* with inhibition diameter of 11.5 and 12 mm, respectively. | [[162](#_ENREF_162)] |
| 27 | *Tricleocarpa fragilis* | Atep Oki Village, East Lembean District, Minahasa Regency, Province of North Sulawesi, Indonesia | Ethanol extract | Antibacterial activity against *Vibrio harveyi* with MIC value of 0.495 µg/mL. | [[163](#_ENREF_163)] |
| 28 | *Ulva lactuca* | Lombok Island, from Batu Layar (8.496720, 116.054625), Tanjung Aan (8.908579, 116.322234), and Seriwe (8.886556, 116.507750), Indonesia | Ethanol extracts | Antioxidant (DPPH) radical scavenging activity with a low IC_50_ of 522.23 ± 43 μg/mL. | [[164](#_ENREF_164)] |
| 29 | *Ulva prolifera* | The coast of Pagkalitan (Lat. 13° 38' 4.2" N; Long. 121° 2' 39" E), Batangas, Philippines. | Acidified methanol extract | Antioxidant efficiency of *U. prolifera* exerted high ability of reducing copper ions, potent ABTS+ and DPPH scavenging activities in a concentration-dependent manner with IC50 value of 24.7 mg GAE/mL, 43.52 mg GAE/mL, and 54.1 mg GAE/mL, respectively, more effective than ascorbic acid.  Antibacterial activity against Methicillin-resistant *Staphylococcus aureus* (MIC = 125 mg/mL), *S. epidermidis* (MIC = 125 mg/mL) and *Pseudomonas fluorescens* (MIC = 500 mg/mL). | [[165](#_ENREF_165)] |

**Supplementary Table 5. Medicinal potential of mangrove species from Indonesia (superscript letters in the same row indicate information belonging to the same reference.**

**Mangrove: 34 References, 8 Compounds)**

| **No.** | **Type (Family)** | **Species** | **Location** | **Constituent/ Extracts** | **Tissue** | **Bioactivity** | **Ref.** |
| --- | --- | --- | --- | --- | --- | --- | --- |
| 1. | Acanthaceae | *Acanthus ilicifolius* L. | East Kalimantan^a^, North Sumatra^b^, East Java^c^ | Ethanol, water and seawater extract ^a^; ethanol, aqueous, and *n*-hexane extract^b^; methanol extract ^c^. | Leaf^ab^, Flower^c^ | Antimicrobial^a^, anthelmintic^b^, antioxidant and anticancer^c^, LC_50_= 22 µg/mL | [[166](#_ENREF_166)] ^a^; [[167](#_ENREF_167)] ^b^; [[168](#_ENREF_168)] ^c^ |
|  |  | *Avicennia alba* Blume | Lombok^a^, East Kalimantan^b^ | Ethanol extract ^a^ | Leaf^ab^ | Anti-coagulants^a^, anticancer^b^, IC_50_ of 74.75 ± 26.97, 50.76 ± 9.92, and 73.25 ± 17.63mg/mL, | [[169](#_ENREF_169)] ^a^; [[170](#_ENREF_170)] ^b^ |
|  |  | *Avicennia marina* (Forssk.) Vierh. | Lampung^ad^, East Kalimantan^b^, Central Java^c^ | Methanol extract ^a^; ethanol, water and seawater extract ^b^; ethanol extract ^d^ | Leaf^abc^, Stem^d^ | Anticancer^a^ IC_50_ = 321 μg/mL, antimicrobial^b^ MIC = 3.26 μg/mL, Sunburn treatment^c^, and antihiperuricemic^d^, IC_50_= 202.4 ug/mL | [[171](#_ENREF_171)]^a^; [[166](#_ENREF_166)]^b^; [[170](#_ENREF_170)]^c^; [[172](#_ENREF_172)]^d^ |
| 2 | Aizoaceae | *Sesuvium portulacastrum* (L.) L. | North Sumatra | Ascorbic acid, β-carotene, and phenolic acid | Fruit | Antioxidant | [[173](#_ENREF_173)] |
| 3 | Arecaceae | *Nypa fruticans* Wurmb | North Sumatra^a^, North Sumatra^b^ | Ascorbic acid, β-carotene, and phenolic acid ^a^; polyisoprenoids ^b^ | Fruit^a^, Leaf^b^ | Antioxidant^a^, anticancer^b^, IC_50_ value180.2 µg/mL, | [[173](#_ENREF_173)]^a^; [[174](#_ENREF_174)]^b^ |
| 4 | Combretaceae | *Lumnitzera littorea* (Jack) Voigt | Across Indonesia | 4-(4-hydroxyphenyl)-2-butanol; 2-sulfate 4-(4-sulfoxy-3-methoxyphenyl)-2-butanone; zingeron sulfate; ellagic acid; methylellagic acid sulfate; 3,4-*O*-dimethylellagic acid; 3,3’,4’-trimethyl ellagic acid 4-sulfate and 3,3’,4’-trimethyl ellagic acid | Roots | The tested extracts showed anti-infective activity with IC _50_ value better than control | [[175](#_ENREF_175)] |
|  |  | *Lumnitzera racemosa* Willd. | Across Indonesia | 4-(4-hydroxyphenyl)-2-butanol; 2-sulfate 4-(4-sulfoxy-3-methoxyphenyl)-2-butanone; zingeron sulfate; ellagic acid; methylellagic acid sulfate; 3,4-*O*-dimethylellagic acid; 3,3’,4’-trimethyl ellagic acid 4-sulfate and 3,3’,4’-trimethyl ellagic acid | Roots | The tested extracts showed anti-infective activity with IC _50_value better than control | [[175](#_ENREF_175)] |
| 5 | Lecythidaceae | *Barringtonia asiatica* (L.) Kurz | North Sumatra | Ascorbic acid, β-carotene, and phenolic acid | Fruit | Antioxidant | [[173](#_ENREF_173)] |
| 6 | Lythraceae | *Sonneratia alba* Sm. | Jambi^a^, East Kalimantan^b^, East Java^c^, Jambi^d^, East Java^e^ | Methanolic extract^a^, ethanol, water and seawater extracts^b^  3β-hydroxy-lup-9(11),12–diene, 28-oic acid ^c^; lupeol ^c^; lupan-3β-ol ^c^; stigmasterol and β-sitosterol ^d^; and ethanol extract ^e^ | Leaf^abcd,e^ , Root^d^ , Bark^e^ | Antisplasmodial and antimalaria^a^, antimicrobial^b^, antibacterial^c^, antioxidants^d,e^, IC_50_ value 223.67 and 439.71 ppm, 67.211 ± 2,482 µg/mL and 88,706 ± 4,782 µg/mL, respectively | [[176](#_ENREF_176)] ^a^; [[166](#_ENREF_166)]^b^; [[177](#_ENREF_177)]^c^; [[178](#_ENREF_178)]^d^; [[179](#_ENREF_179)]^e^ |
| 7 | Lythraceae | *Sonneratia caseolaris* (L.) Engl. | South Sulawesi | Ethyl acetate and ethanol extract | Fruits | Antioxidant and antimicrobial | [[180](#_ENREF_180)] |
| 8 | Malvaceae | *Hibiscus tilliaceus* L. | North Sumatra | Ascorbic acid, β-carotene, and phenolic acid | Fruit | Antioxidant | [[173](#_ENREF_173)] |
| 9 | Melastomataceae | *Melastoma candidum* D. Don | North Sumatra | Ascorbic acid, β-carotene, and phenolic acid | Fruit | Antioxidant | [[173](#_ENREF_173)] |
| 10 | Meliaceae | *Xylocarpus granatum* J.Koenig | Central Java^ab^, Lampung^c^ | Extract ^a^; ethanol extract ^b^; fractionized ethyl acetate extract ^c^ | Fruit^a^, leaf^bc^, all tissues^b^. | Wound treatment^a^, antioxidant and anti-ageing^b^, anticancer^c^, IC_50_ 23.12 µg/mL | [[181](#_ENREF_181)]^a^; [[182](#_ENREF_182)]^b^; [[183](#_ENREF_183)]^c^ |
| 11 | Meliaceae | *Xylocarpus moluccensis* (Lam.) M.Roem | North Sulawesi | Methanolic fractions | Stem bark | Antioxidant | [[184](#_ENREF_184)] |
| 12 | Pandanaceae | *Pandanus odoardii* Martelli | North Sumatra | Ascorbic acid, β-carotene, and phenolic acid | Fruit | Antioxidant | [[173](#_ENREF_173)] |
| 13 | Primulaceae | *Aegiceras corniculatum* (L.) Blanco | Central Java^a^, East Kalimantan^b^ | Ethanol extract ^a^ | Leaf^a,b^ | Antioxidant, anti-inflammatory, and antibacterial^a^, anticancer^b^, IC_50_ of 49.40 ± 5.85, 78.12 ± 11.38, and 45.60 ± 7.35mg/mL | [[185](#_ENREF_185)]^a^; [[170](#_ENREF_170)]^b^ |
| 14 | Pteridaceae | *Acrostichum aureum* L. | East Kalimantan | - | Leaf | Anticancer, IC_50_ of 89.19 ± 4.22 mg/mL | [[170](#_ENREF_170)] |
| 15 | Rhizophoraceae | *Bruguiera cylindrica* (L.) Blume | Jakarta | Water, ethanol, chloroform and *n*-hexane extract | Leaf | Antibacterial | [[186](#_ENREF_186)] |
|  |  | *Bruguiera gymnorhiza* (L.) Lam. | East Lampung^ab^, Jakarta^c^, North Sulawesi^d^ | Ethyl acetate extract^ad^; ethanol extract^b^; methanol extract^c^ | Stems^a^, leaf^b^, leaf^c^, fruit^d^ | Antioxidant^a^ IC_50_ = 255.03 mg/L and antibacterial^a^ MIC = 62.5 mg/L, wound treatments^b^, antioxidant^c^, IC_50_ of 81.11 ppm, antimicrobial^d^, LC_50_ of 271.6439 ppm | [[187](#_ENREF_187)] ^a^; [[188](#_ENREF_188)] ^b^; [[189](#_ENREF_189)]^c^; [[190](#_ENREF_190)]^d^ |
|  |  | *Ceriops decandra* (Griff.) W.Theob. | Aceh | Methanol extract | Bark | Anticancer, LC_50_ of 31.5 μg/mL, | [[191](#_ENREF_191)] |
|  |  | *Ceriops tagal* (Perr.) C.B.Rob. | North Sumatra | Dolichol | Leaf | Anticancer | [[192](#_ENREF_192)] |
|  |  | *Rhizophora apiculata* Blume | Bali^a^, Lampung^b^, Central Java^c^ | Ethanol extract^a^; methanol, chloroform, and *n*-hexane extract^b^; methanol extract^c^ | Root bark^a^, root^b^, leaf^c^ | Antimicrobial^a^ Inhibition zone = 6.27-9.87 mm, antimicrobial^b^ Inhibition zone = 8.64-19.83 mm, anticancer^c^, IC_50_ of 0.0323 mg/mL. | [[193](#_ENREF_193)]^a^; [[194](#_ENREF_194)]^b^; [[195](#_ENREF_195)]^c^ |
|  |  | *Rhizophora mucronata* Lam. | East Java^a^, North Sumatra^b^, East Kalimantan^c^ West Java^d^, North Sulawesi^e^, | Ethanol extract^ade^; dolichol^b^ | Leaf ^a,b, d,e^, fruits^c.^ | Analgesics^a^, anticancer^bc^, IC_50_ of 57.49 ± 11.70 mg/mL, antioxidant^de^, IC_50_ of 0.72 ppm; IC_50_ of 20.99 ± 0.33 μg/mL, | [[196](#_ENREF_196)]^a^; [[192](#_ENREF_192)]^b^; [[170](#_ENREF_170)]^c^; [[197](#_ENREF_197)]^d^; [[198](#_ENREF_198)]^e^ |
|  |  | *Rhizophora stylosa* Griff. | East Kalimantan^a^, West Java^b^ | Ethanol, water and seawater extract ^a^; lupeol and β-sitosterol ^b^ | Leaf ^a^, stem bark^b^ | Antimicrobial^a^, anticancer^b^ | [[166](#_ENREF_166)]^a^; [[199](#_ENREF_199)]^b^ |
| 16 | Verbenaceae | *Stachytarpheta jamaicensis* (L.) Vahl | North Sumatra | Ascorbic acid, β-carotene, and phenolic acid | Fruit | Antioxidant | [[173](#_ENREF_173)] |

**Supplementary Table 6. Medicinal potential of marine micro fungi species from Indonesia**

| **No** | **Biological Source of Fungi** | **Sample Location** | **Fungi species** | **Compounds/Extract** | **Bioactivity** | **Ref.** |
| --- | --- | --- | --- | --- | --- | --- |
| 1 | Ascidian (*Eudistoma sp*) | Bunaken waters of North Sulawesi, Indonesia (01°36’49.46’’N, 124°46’03.17’’ S) at a depth of 7m | *Trichoderma asperellum* | crude extract | Antioxidant (DPPH, IC_50_ 32.89 µg/mL), Anti-microbial (Inhibition diameter, *Candida albicans, Aeromonas hydrophila, Escherichia coli, Salmonella sp., Staphylococcus aureus* 8.5 ± 0.3, 15 ± 0.17, 9.5 ± 0.56, 10 ± 0.44, and 8.5 ± 0.20 mm, respectively). | [[200](#_ENREF_200)] |
| 2 | Ascidian (*Polycarpa aurata*) | Manado, Indonesia | *Penicillium verruculosum* | verruculides A and B,  chrodrimanins A, B, and H | Verruculides A, chrodrimanins A, and chrodrimanins H inhibited the activity of protein tyrosine phosphatase 1B (PTP1B) with IC_50_ values of 8.4, 8.5, and 14.9 µM, respectively. Verruculides B showed 40% inhibition at 23.1 µM, while chrodimanins B was not active at concentration 20.7 µM. | [[201](#_ENREF_201)] |
| 3 | Ascidian (unidentified coronal ascidian) | Manado, Indonesia | *Penicillium albobiverticillium* | 2-hydroxy-6(20-hydroxy-30-hydroxymethyl-5-methylphenoxy)benzoic acid, monodictyphenone | Monodictyphenone moderate inhibitory activities against protein tyrosine phosphatase (PTP) 1B, T cell PTP (TCPTP), and CD45 tyrosine phosphatase (CD45); 2-hydroxy-6(20-hydroxy-430-hydroxymethyl-5-methylphenoxy)-benzoic acid modestly inhibited CD45 activity. | [[202](#_ENREF_202)] |
| 4 | Green alga (*Bornetella* sp.) | Malalayang Beach, Manado, North Sulawesi | *Aspergillus nominus* | extract | Preliminary antibacterial screening against *Staphylococcus aureus* and *Escherecia coli.* | [[203](#_ENREF_203)] |
| 5 | Mangrove (*Aegiceras corniculatum*) | Segara Anakan, Central Java | Isolate DD1, DD2, DBU1, DBU2, DBU3; *Microdochium sp* DR1, DR2, DR3 | extract. GCMS on DBU3 (phenol,3,5-*bis*(1,1-dimethylethyl), hexadecanoic acid, hexadecanoic acid methyl ester, malic acid, *N*-aminopyrrolidine, 9-octadecanoic acid, methyl ester (*E*), benzeneethanol, 4-hydroxy, 1,2-benzenedicarboxylic acid, *D*-tyrosine, *bis*(2-methylpropyl) ester 1-nonadecene dan heneicosane) | Antioxidant (DPPH, percentage inhibition of DPPH scavenger, range 7.45-89.80%). DBU3 IC_50_ 19.28 µg/mL). | [[204](#_ENREF_204)] |
| 6 | Mangroves (*Avicennia* sp., *Sonneratia* sp., *Rhizophora* sp.) | Manado, North Sulawesi  Likupang Restoration (MSr), 1°40'33.82"N/125° 3'17.24"E; Likupang Natural (MSn), 1°40'41.76"N/125° 3'14.20"E; Tiwoho Natural (MT), 1°35'57.01"N/124°51'32.25"E; Bawoho Restoration (MBa), 1°34'51.69"N/124°49'3.26"E; Buyat Restoration (MB), 0°50'57.01"N/124°42'27.56"E | *Trichoderma viride* (tiwoho), *Penicillium citrimum* (bahowo) | extract | The extract showed varying antimicrobial activity against *V. harveyi*, *V. vulnificus*, and *V. Parahaemolyticus*. *Trichoderma viride* showed the strongest activity against the tested Vibrio species. | [[205](#_ENREF_205)] |
| 7 | Mangroves *Avicennia marina* | Payung island, Banyuasin Regency, South Sumatera | *Aspergillus flavus, Aspergillus ochraceus* | extract | Antibacterial (*A. flavus* inhibition diameter range *S. aureus*, 13.79 ± 1.26 mm, *E. coli* 11.86 ± 1.48 mm), (*A. ochraceus* 8.01 ± 0.45 mm, 9.34 ± 1.25 mm). | [[206](#_ENREF_206)] |
| 8 | Sponges | Kotok Kecil, Seribu island, North Jakarta | K1-K9 | Secondary metabolites of K1 (butylated hydroxytoluene, 2 (5*H*)-furanone, 4-methyl-5-2(2-propenyl), 1-octadecene, cycloeicosane, phthalic acid, di(2-propylpetyl)ester,  2-(acetoxymethyl)-3-(methoxycarbonyl)biphenylene | Antimicrobial (*Escherichia coli,* inhibition diameter, range 6-8.40 mm; *Streptococcus mutans,* range 6-7.45 mm). | [[207](#_ENREF_207)] |
| 9 | Sponges | Singaraja, Buleleng, Bali | Isolate A02F, A05RF, A06RF, A09RF, A12RF, close to *Aspergillus sp*A13RF, A14RF, B14RF, B15RF  B19RF, C20RF, C21RF, C23RF, C24RF, C27RF, C30RF, C31RF, D36RF, E41RF, E42RF | ethyl acetate extract | The ethyl acetate extracts of A12RF, A05RF, C36RF had inhibitory activity against the growth of *S. aureus* at a concentration of 0,5mg/mL. | [[208](#_ENREF_208)] |
| 10 | Sponges (*Ancorina* sp.) | Kukup Beach, Yogyakarta | SAF KU4 | ethyl acetate extract | Cytotoxicity (HeLa, IC_50_ 283.95 µg/mL) | [[209](#_ENREF_209)] |
| 11 | Sponges (*Clathria* sp) | Pramuka island, North Jakarta  (5º45'48" S and 106º37'11" E) | *Gymnoascus udagawae* IPBCC 19.1495  *Gymnoascus udagawae* IPBCC 19.1496 | ethyl acetate extract | Both ethyl acetate extracts of *G. udagawae* IPBCC 19.1495 and IPBCC 19.1496 showed anti-fungal activity against *C. albicans* with inhibition diameter of 14.0 ± 0.71 and 14.4 ± 0.89 mm, respectively at concentration of 500 mg/µL. | [[210](#_ENREF_210)] |
| 12 | Sponges (*Monanchora* sp.) | Samalona island, Makassar, South Sulawesi  (5° 07’ 37,410” S and 119° 20’ 24,010” E) | *Trichoderma reesei* | extract | Antibacterial (500 µg/disk, inhibition diameter *V. harveyi* 4.82 ± 0.37 mm, *V. parahaemolyticus* 3.83 ± 0.2 mm, *V. vulnificus* 4.14 ± 0.32 mm). | [[211](#_ENREF_211)] |
| 13 | Sponges (*Stylissa sp*) | Pramuka island, North Jakarta | *Purpureocillium lilacinum* | LCMS detection (galangin, kaempferol, quercetin) | Anti-obesity (pancreatic lipase inhibition, active fraction IC_50_= 220.60 µg/mL) | [[212](#_ENREF_212)] |

**Supplementary Table7.** Bioactive compounds isolated from marine bacteria in Indonesia and their pharmacological potential (21 References, 10 Compounds)

| **No** | **Biological Source of Bacteria** | **Sample Location** | **Bacteria species** | **Compounds/Extract** | | **Bioactivity** | **Ref.** | | |
| --- | --- | --- | --- | --- | --- | --- | --- | --- | --- |
| 1 | Mangrove sediments | Setokok Island, Indonesia | *Pseudoalteromonas xiamenensis* | triethoxy-borane; 1,3-diphenyl-1,3,5,5-tetramethyl-cyclotrisiloxane; 1,6-diazaspiro (4.4) nonane-2,7-dione; and 1,3-*bis* (4-methoxyphenyl)-5-phenyl-1,3,5-triazinane-2-thione | Antibacterial (*Vibrio* spp. MIC ≤0.78 µg/mL). | | | [[213](#_ENREF_213)] |  |
| 2 | Mollusca; chepalopoda *Loligo sp., Loligo Edulis* | Jepara and the Bombay duck (Harpadon nehereus) from the Strait of Malacca, Indonesia at the depth of 1-2 m | LDS 12-4 (Uncultured bacterium clone 1P-1-G05), LDS 18-5 (Uncultured bacteriumclone 3g10a) | Isolate extract | Antibacterial (LDS 12-4, *E. coli*, inhibition zone 32.59 mm, LDS 18-5, *Enterobacter sp*, inhibition zone 28.44 mm). | | | [[214](#_ENREF_214)] |  |
| 3 | Nudibranchs | Lovina, Bali and the National Park of Karimunjawa, Jepara at 15-20 m in depth | Symbiont bacteria of Nudibranchs. *Pseudoalteromonas rubra* | Isolate extract. LCMS detection stearidonic acid, prodigiosin, (22*E*,24*R*)-5a8a-epidioxyergosta-6,9,22-trien-3*β*-ol | Cytotoxicity (*Pseudoalteromonas rubra,* Vero cell CC_50_ 75 µg/mL). | | | [[215](#_ENREF_215)] |  |
| 4 | Red algae *Gelidiella acerosa* | Drini, Gunungkidul Yogyakarta | 14 actinobacteria isolates | Isolate extract | Eight out of 14 actinobacteria isolates showed an antibacterial activity against *Vibrio alginolyticu*. | | | [[216](#_ENREF_216)] |  |
| 5 | Sea Cucumber *Holothuria leucospilota* and *Stichopus vastus* | Lampung, Indonesia | 127 bacteria isolates | - | Preliminary screening on ESKAPE bacteria. | | | [[217](#_ENREF_217)] |  |
| 6 | Sea slugs, sponge | Bunaken National Park, located near Manado, North Sulawesi, Indonesia | *Vibrio* and *Pseudoalteromonas* strains | Isolate extract | From the obtained isolates, 35 out of 49 strains showed antibacterial activity against *E. coli* XL1Blue. All of the 10 isolated *Pseudoalteromonas* strains showed activity, and 7 thereof revealed strong activity against the Gram-positive test strain. Further, two strains also inhibited methicillin resistant *S. aureus* (MRSA) | | | [[218](#_ENREF_218)] |  |
| 7 | Sediment samples | Karimunjawa National Park, Jepara regency and Mangkang sub-district, Semarang city, Central Java | *Pseudomonas aeruginosa*, *Zhouia amylolytica* strain HN-181, *P. aeruginosa* strain QK -2 | Isolate extract | Isolate SP 3-8 (*P. aeruginosa* strain QK -2) tested positive against *C. albicans* (0.58 ± 0.03 mm), *Trichoderma* sp. (1.31 ± 0.07 mm) and *A. niger* (0.80 ± 0.06 mm). | | | [[219](#_ENREF_219)] |  |
| 8 | Soft coral *Sinularia polydactyla* | Bandengan water, Jepara, North Java Sea, Indonesia at a depth of 5 m | *Pseudomonas* sp.(TASC.16) and *Streptococcus equi* Subsp. *zooepidemicus* (K6.72) | Isolate extract | Isolate TASC 16 :  Inhibition zone : 15,50 ; 16.10 ; 15.70 mm against pathogenic K6.72. | | | [[220](#_ENREF_220)] |  |
| 9 | Sponge | Research Center for Oceanography, Indonesian Institutes of Sciences (LIPI) collection | Nine sponge endophytic bacterial strains:  Sp 4.3, Sp 5.8,Sp 6.3, Sp 7.4, Sp 7.5, Sp 7.9, Sp 8.5, Sp 8.9, and Sp 8.10 | Isolate extract | Antibacterial (*Staphylococcus aureus, Bacillus subtilis, Pseudomonas aeuriginosa,* MIC range 2.5-10.0 mg/mL). | | | [[221](#_ENREF_221)] |  |
| 10 | Sponge | - | Sponge-associated bacteria HAA-01, HAL-13, and HAL-74 | Isolate extract | Antibacterial (*E. coli*, EPEC, *Pseudomonas aeruginosa, S. aureus, B. subtilis*, MIC range 0.1-1.0 mg/mL), Cytotoxixity (Hela, IC_50_ range 132.88-457.64 µg/mL). | | | [[222](#_ENREF_222)] |  |
| 11 | Sponge *Haliclona fascigera* | Setan Island, South Coast of West Sumatra, Indonesia at a depth of 20 m | N_1_M_1,_ N2M2, N2M, N1M2, N2M, H1N1, H2N2, H2N1, MR1, M2R2, M2R1, NF1, N2F1, and N1F1 | Isolate extract | Twelve isolates of the bacteria were considered active to MRSA. Mean of inhibition zones ranged from 11.1 ± 0.17 to 15.17 ± 0.76. The most effective extract against MRSA was bacteria N1F2 (*Corynebacterium* sp.4). | | | [[223](#_ENREF_223)] |  |
| 12 | Sponge *Haliclona fascigera* | Setan Island, West Sumatra, Indonesia at a depth of 13 m | *Bacillus* sp., *Corynebacterium* sp., *Micrococcus* sp., *Staphylococcus aureus* | Isolate extract | Cytotoxicity (Hela, Widr, 347D, LC_50_ range 31.17 - 283.38 µg/mL). | | | [[224](#_ENREF_224)] |  |
| 13 | Sponge *Reniera* sp. | Karimunjawa Islands, Jepara | 46 bacteria associated with sponge *Reniera* sp. | Isolate extract | Antibacterial (*E. coli*, MRSA, inhibition zone diameter range 2.3 - 10.7 mm). | | | [[225](#_ENREF_225)] |  |
| 14 | Sponge *Xestospongia testudinaria* | Tanjung Kasuari, Sorong, Papua. At the depth of 15-20 m | Xp 4.1, Xp 4.2, Xp 4.3, Xp 4.4, Xp 4.5 and Xp 4.6 | Isolate extract | Xp 4.2 appeared to be the most active isolate in antibacterial testing against *E.coli* and *K. pneumoni* with inhibition diameter of 24 and 22 mm, respectively at concentration of 1 mg/mL. | | | [[226](#_ENREF_226)] |  |
| 15 | Sponges | Kodingareng Keke Island-Makassar Indonesia | KDR-02-01, KDR-02-02, KDR-02-03, KDR-02-04, KDR-02-05, KDR-02-06, KDR. -02-07, KDR-02-08, KDR-02-09, and KDR-02-10 | Isolate extract | The bcterial isolate KDR-02-07 showed the best inhibition compared to other isolates capable of inhibiting *E. coli*, *S. aureus* and *C. albicans*. | | | [[227](#_ENREF_227)] |  |
| 16 | Sponges and corals | Baru Island, Randayan Island, and Lemukutan Island, Bengkayang District, West Kalimantan at a depth of 7 m | Extracts of 12 bacterial isolates from sponges or corals:  RC1, RC2, RC3, RC4, RC5  RS1, RS2, RS3, RS4, RS5, RS6, RS7 | Isolate extract | All 12 of the strains showed the ability to produce potent antibacterial compounds, and some of them were active against the opportunistic Gram-negative bacterial pathogen *P.aeruginosa* with inhibition diameter range 0-30 mm. | | | [[228](#_ENREF_228)] |  |
| 17 | Sponges *Clathrina* sp. and *Agelas* sp. | Sibolga, North Sumatra at the depth of ± 10-20 m | Eleven and 7 isolates of symbiont bacteria from sponges *Clathrina* sp. and *Agelas* sp., respectively:  C1I1, C1I2, C1I3, C1I4, C1I5, C1I6, C1I7, C1I8, C1I9, C1I10, C1I11  A1I1, A1I2, A1I3, A1I4, A1I5, A1I6, A1I7 | Isolate extract | Antibacterial (C1I1, C1I3, C1I5, C1I6, C1I9, C1I10, A1I3, against KP, PA, SL, inhibition zone range 6.0-9.3 mm). | | | [[229](#_ENREF_229)] |  |
| 18 | Sponges *Hyrtios* sp., *Verongula* sp. and *Smenospongia* sp. | Kepulauan Seribu Jakarta | Bacteria isolates from marine sponges *Hyrtios* sp., *Verongula* sp. and *Smenospongia* sp.:  D4.13, D4.15, D4.19, D4.110, P2.24, P2.211, P2.212, P2.34, P3.310, P3.414, P3.415, P3.514 | Isolate extract | Isolate coded as P2.24 was the only bacterium that had the widest spectrum of anti-Vibrio bioactive compounds against three *Vibrio* sp used i.e., *Vibrio harveyi*, *V. parahaemolyticus*, *V. vulnificus*. Consistently, an anti- *Vibrio* sp. activity of the P2.24 was also shown by antagonism assay using culture, supernatant and crude extract of the isolate. | | | [[230](#_ENREF_230)] |  |
| 19 | The surface and the bottom layer of seawater (depths of 0 and 6 m). | Alor Island at the depths of 0 and 6 m | *Pseudoalteromonas rubra* strains PS1 and SB14 | Both strains produced cycloprodigiosin; 2-methyl-3-hexyl-prodiginine, and prodigiosin,  2-methyl-3-propyl prodiginine, 2-methyl-3-butyl prodiginine, and 2-methyl-3-heptyl-prodiginine | The antimicrobial activity of cycloprodigiosin, prodigiosin, and 2-methyl-3-hexyl-prodiginine was examined by a disk-diffusion test against *Escherichia coli*, *Staphylococcus aureus*, *Salmonella typhi*, and *Candida albicans*, in which the test results showed that at a concentration of 20 μg/mL, cycloprodigiosin exhibited the greatest inhibition (25.1 ± 0.55 mm) against *S. aureus*. | | | [[231](#_ENREF_231)] |  |
| 20 | Water sample | Lombok Island, Indian Ocean | *Bacillus* sp. (*Bacillus flexus, B. methylotrophicus, B. aquimaris, B. horikoshii, and B. thioparans*) | Isolate extract | Selected strains from the Lombok Strait and Indian Ocean were capable of degrading crude oil (2% v/v) by 43.9–71.9% over 14 days. | | | [[232](#_ENREF_232)] |  |
| 21 | Water sample | Sea water of Siak, Riau, Indonesia | [Bacillus cereus](https://www.sciencedirect.com/topics/medicine-and-dentistry/bacillus-cereus) SN7 and *Vagococcus fluvialis* CT21 | Isolate extract | The anti-pathogenic activity test demonstrated the ability for both bacteria to inhibit three types of pathogens with the following inhibitory zone values: *Vibrio*  *alginolyticus* (10–11 mm), *Aeromonas hydrophila* (8–12 mm), and *Pseudomonas aeruginosa* (8–10 mm). | | | [[233](#_ENREF_233)] |  |

References

1. Diers JA, Bowling JJ, Duke SO, Wahyuono S, Kelly M, Hamann MT. Zebra mussel antifouling activity of the marine natural product aaptamine and analogs. Marine Biotechnology. 2006;8:366-372.

2. Tsukamoto S, Yamanokuchi R, Yoshitomi M, Sato K, Ikeda T, Rotinsulu H, Mangindaan REP, de Voogd NJ, van Soest RWM, Yokosawa H. Aaptamine, an alkaloid from the sponge *aaptos suberitoides*, functions as a proteasome inhibitor. Bioorg. Med. Chem. Lett. 2010;20:3341-3343.

3. Abdillah S, Nurhayati APD, Nurhatika S, Setiawan E, Heffen WL. Cytotoxic and antioxidant activities of marine sponge diversity at pecaron bay pasir putih situbondo east java, indonesia. J. Pharm. Res. (Gurgaon, India). 2013;6:685-689.

4. Elkhayat E, Edrada R, Ebel R, Wray V, van Soest R, Wiryowidagdo S, Mohamed MH, Mueller WEG, Proksch P. New luffariellolide derivatives from the indonesian sponge *acanthodendrilla* sp. Journal of Natural Products. 2004;67:1809-1817.

5. Williams DE, Telliez J-B, Liu J, Tahir A, van Soest R, Andersen RJ. Meroterpenoid mapkap (mk2) inhibitors isolated from the indonesian marine sponge *acanthodendrilla* sp. Journal of Natural Products. 2004;67:2127-2129.

6. El-Desoky AH, Kato H, Eguchi K, Kawabata T, Fujiwara Y, Losung F, Mangindaan REP, de Voogd NJ, Takeya M, Yokosawa H, Tsukamoto S. Acantholactam and pre-neo-kauluamine, manzamine-related alkaloids from the indonesian marine sponge *acanthostrongylophora ingens*. J. Nat. Prod. 2014;77:1536-1540.

7. Murti YB, Isolation and structure elucidation of bioactive secondary metabolites from sponges collected at ujungpandang and in the bali sea, indonesia. 2006. p. No pp.

8. Ibrahim SRM, Mohamed GA, Zayed MF, Sayed HM. Ingenines a and b, two new alkaloids from the indonesian sponge *acanthostrongylophora ingens*. Drug Res. (Stuttgart, Ger.). 2015;65:361-365.

9. AlTarabeen M, Daletos G, Ebrahim W, Muller WEG, Hartmann R, Lin W, Proksch P. Ircinal e, a new manzamine derivative from the indonesian marine sponge *acanthostrongylophora ingens*. Nat Prod Commun. 2015;10:1951-3.

10. Ibrahim SRM Mohamed GA. Ingenines c and d, new cytotoxic pyrimidine-*β*-carboline alkaloids from the indonesian sponge *acanthostrongylophora ingens*. Phytochemistry Letters. 2016;18:168-171.

11. Esposito G, Mai LH, Longeon A, Mangoni AA-O, Durieu E, Meijer LA-O, Van Soest R, Costantino VA-O, Bourguet-Kondracki ML. A collection of bioactive nitrogen-containing molecules from the marine sponge *acanthostrongylophora ingens*. Marine drugs. 2019;17:472.

12. Rao KV, Kasanah N, Wahyuono S, Tekwani BL, Schinazi RF, Hamann MT. Three new manzamine alkaloids from a common indonesian sponge and their activity against infectious and tropical parasitic diseases. Journal of Natural Products. 2004;67:1314-1318.

13. Rao KV, Donia MS, Peng J, Garcia-Palomero E, Alonso D, Martinez A, Medina M, Franzblau SG, Tekwani BL, Khan SI, Wahyuono S, Willett KL, Hamann MT. Manzamine b and e and ircinal a related alkaloids from an indonesian *acanthostrongylophora* sponge and their activity against infectious, tropical parasitic, and alzheimer's diseases. Journal of Natural Products. 2006;69:1034-1040.

14. Peng J, Hu J-F, Kazi AB, Li Z, Avery M, Peraud O, Hill RT, Franzblau SG, Zhang F, Schinazi RF, Wirtz SS, Tharnish P, Kelly M, Wahyuono S, Hamann MT. Manadomanzamines a and b: A novel alkaloid ring system with potent activity against mycobacteria and hiv-1. Journal of the American Chemical Society. 2003;125:13382-13386.

15. Hamann M, Alonso D, Martin-Aparicio E, Fuertes A, Perez-Puerto MJ, Castro A, Morales S, Navarro ML, del Monte-Millan M, Medina M, Pennaka H, Balaiah A, Peng J, Cook J, Wahyuono S, Martinez A. Glycogen synthase kinase-3 (gsk-3) inhibitory activity and structure-activity relationship (sar) studies of the manzamine alkaloids. Potential for alzheimer's disease. Journal of Natural Products. 2007;70:1397-1405.

16. Wahba AE, Fromentin Y, Zou Y, Hamann MT. Acantholactone, a new manzamine related alkaloid with an unprecedented *δ*-lactone and *ε*-lactam ring system. Tetrahedron Lett. 2012;53:6329-6331.

17. Hertiani T, Edrada-Ebel R, Ortlepp S, van Soest RWM, de Voogd NJ, Wray V, Hentschel U, Kozytska S, Mueller WEG, Proksch P. From anti-fouling to biofilm inhibition: New cytotoxic secondary metabolites from two indonesian *agelas* sponges. Bioorg. Med. Chem. 2010;18:1297-1311.

18. Dai J, Parrish SM, Yoshida WY, Yip MLR, Turkson J, Kelly M, Williams P. Bromotyrosine-derived metabolites from an indonesian marine sponge in the family aplysinellidae (order verongiida). Bioorg. Med. Chem. Lett. 2016;26:499-504.

19. Mudianta IW, Skinner-Adams T, Andrews KT, Davis RA, Hadi TA, Hayes PY, Garson MJ. Psammaplysin derivatives from the balinese marine sponge *aplysinella strongylata*. J. Nat. Prod. 2012;75:2132-2143.

20. Gallimore WA, Cabral C, Kelly M, Scheuer PJ. A novel d-ring unsaturated a-nor sterol from the indonesian sponge, *axinella carteri* dendy. Nat. Prod. Res. 2008;22:1339-1343.

21. Supriyono A, Schwarz B, Wray V, Witte L, Mueller WEG, van Soest R, Sumaryono W, Proksch P. Bioactive alkaloids from the tropical marine sponge *axinella carteri*. Zeitschrift fuer Naturforschung, C: Biosciences. 1995;50:669-74.

22. Williams DE, Patrick BO, Tahir A, Van Soest R, Roberge M, Andersen RJ. Boneratamides a-c, new sesquiterpenoids isolated from the marine sponge *axinyssa aplysinoides*. Journal of Natural Products. 2004;67:1752-1754.

23. Hertiani T, Edrada-Ebel RA, Kubbutat M, van Soest RWM, Proksch P. Protein kinase inhibitors from indonesian sponge *axynissa* sp. Maj. Farm. Indones. 2008;19:78-85.

24. Aoki S, Wei H, Matsui K, Rachmat R, Kobayashi M. Pyridoacridine alkaloids inducing neuronal differentiation in a neuroblastoma cell line, from marine sponge *biemna fortis*. Bioorganic & Medicinal Chemistry. 2003;11:1969-1973.

25. Ibrahim SRM, Min CC, Teuscher F, Ebel R, Kakoschke C, Lin W, Wray V, Edrada-Ebel R, Proksch P. Callyaerins a-f and h, new cytotoxic cyclic peptides from the indonesian marine sponge *callyspongia aerizusa*. Bioorg. Med. Chem. 2010;18:4947-4956.

26. Ibrahim SRM, Edrada-Ebel R, Mohamed GA, Youssef DTA, Wray V, Proksch P. Callyaerin g, a new cytotoxic cyclic peptide from the marine sponge *callyspongia aerizusa*. ARKIVOC (Gainesville, FL, United States). 2008;164-171.

27. Daletos G, Kalscheuer R, Koliwer-Brandl H, Hartmann R, de Voogd NJ, Wray V, Lin W, Proksch P. Callyaerins from the marine sponge *callyspongia aerizusa*: Cyclic peptides with antitubercular activity. J. Nat. Prod. 2015;78:1910-1925.

28. Pham C-D, Hartmann R, Boehler P, Stork B, Wesselborg S, Lin W, Lai D, Proksch P. Callyspongiolide, a cytotoxic macrolide from the marine sponge *callyspongia* sp. Org. Lett. 2014;16:266-269.

29. de Voogd NJ. The mariculture potential of the indonesian reef-dwelling sponge *callyspongia* (*euplacella*) *biru*: Growth, survival and bioactive compounds. Aquaculture. 2007;262:54-64.

30. Braekman JC, Daloze D, Devijver C, Dubut D, Van Soest RWM. A new c-20 polyacetylene from the sponge *callyspongia pseudoreticulata*. Journal of Natural Products. 2003;66:871-872.

31. Trianto A, Hermawan I, Suzuka T, Tanaka J. Two new cytotoxic candidaspongiolides from an indonesian sponge. ISRN Pharm. 2011;852619, 6 pp.

32. Williams DE, Hollander I, Feldberg L, Frommer E, Mallon R, Tahir A, van Soest R, Andersen RJ. Scalarane-based sesterterpenoid rce-protease inhibitors isolated from the indonesian marine sponge *carteriospongia foliascens*. J. Nat. Prod. 2009;72:1106-1109.

33. Mokhlesi A, Stuhldreier F, Wex KW, Berscheid A, Hartmann R, Rehberg N, Sureechatchaiyan P, Chaidir C, Kassack MU, Kalscheuer R, Broetz-Oesterhelt H, Wesselborg S, Stork B, Daletos G, Proksch P. Cyclic cystine-bridged peptides from the marine sponge *clathria basilana* induce apoptosis in tumor cells and depolarize the bacterial cytoplasmic membrane. J. Nat. Prod. 2017;80:2941-2952.

34. Kasmiati K, Yoshioka Y, Okamoto T, Ojika M. New crambescidin-type alkaloids from the indonesian marine sponge *clathria bulbotoxa*. Marine Drugs. 2018;16:84.

35. Sahidin S, Sabandar C, Wahyuni W, Hamsidi R, Malaka M, Sadarun B, Aslan L. A-nor steroids from the marine sponge, *clathria* species. Malaysian Journal of Analytical Sciences. 2018;22:375-382.

36. Fattorusso E, Romano A, Taglialatela-Scafati O, Bavestrello G, Bonelli P, Calcinai B. Coelodiol and coeloic acid, ent-isocopalane diterpenes from the indonesian sponge *coelocarteria* cfr. *Singaporensis*. Tetrahedron Letters. 2006;47:2197-2200.

37. Kotoku N, Sumii Y, Hayashi T, Kobayashi M. Synthesis of cd-ring structure of cortistatin a, an anti-angiogenic steroidal alkaloid from marine sponge. Tetrahedron Letters. 2008;49:7078-7081.

38. Aoki S, Watanabe Y, Tanabe D, Setiawan A, Arai M, Kobayashi M. Cortistatins j, k, l, novel abeo-9(10-19)-androstane-type steroidal alkaloids with isoquinoline unit, from marine sponge *corticium simplex*. Tetrahedron Letters. 2007;48:4485-4488.

39. Jurek J, Scheuer PJ, Kelly-Borges M. Two steroidal alkaloids from a sponge, *corticium* sp. Journal of Natural Products. 1994;57:1004-7.

40. Arai M, Kawachi T, Sato H, Setiawan A, Kobayashi M. Marine spongian sesquiterpene phenols, dictyoceratin-c and smenospondiol, display hypoxia-selective growth inhibition against cancer cells. Bioorg. Med. Chem. Lett. 2014;24:3155-3157.

41. Balansa W, Mettal U, Wuisan ZG, Plubrukarn A, Ijong FG, Liu Y, Schäberle TF A new sesquiterpenoid aminoquinone from an indonesian marine sponge. Marine Drugs, 2019. **17**, 158 DOI: 10.3390/md17030158.

42. Chianese G, Fattorusso E, Taglialatela-Scafati O, Bavestrello G, Calcinai B, Dien HA, Ligresti A, Di Marzo V. Desulfohaplosamate, a new phosphate-containing steroid from *dasychalina* sp., is a selective cannabinoid cb2 receptor ligand. Steroids. 2011;76:998-1002.

43. Salmoun M, Braekman JC, Dewelle J, Darro F, Kiss R, De Voogd NJ, Van Soest RWM. New terpenoids from two indonesian marine sponges. Natural Product Research, Part A: Structure and Synthesis. 2007;21:149-155.

44. Handayani D, Edrada RA, Proksch P, Wray V, Witte L, Van Soest RWM, Kunzmann A, Soedarsono. Four new bioactive polybrominated diphenyl ethers of the sponge *dysidea herbacea* from west sumatra, indonesia. Journal of Natural Products. 1997;60:1313-1316.

45. Sadar MD, Williams DE, Mawji NR, Patrick BO, Wikanta T, Chasanah E, Irianto HE, Van Soest R, Andersen RJ. Sintokamides a to e, chlorinated peptides from the sponge *dysidea* sp. That inhibit transactivation of the n-terminus of the androgen receptor in prostate cancer cells. Organic Letters. 2008;10:4947-4950.

46. Trianto A, de VNJ, Tanaka J. Two new compounds from an indonesian sponge *dysidea* sp. J Asian Nat Prod Res. 2014;16:163-8.

47. Mudianta IW, Katavic PL, Lambert LK, Hayes PY, Banwell MG, Munro MHG, Bernhardt PV, Garson MJ. Structure and absolute configuration of 3-alkylpiperidine alkaloids from an indonesian sponge of the genus *halichondria*. Tetrahedron. 2010;66:2752-2760.

48. Aoki S, Matsui K, Wei H, Murakami N, Kobayashi M. Structure-activity relationship of neuritogenic spongean acetylene alcohols, lembehynes. Tetrahedron. 2002;58:5417-5422.

49. Rao KV, Santarsiero BD, Mesecar AD, Schinazi RF, Tekwani BL, Hamann MT. New manzamine alkaloids with activity against infectious and tropical parasitic diseases from an indonesian sponge. Journal of Natural Products. 2003;66:823-828.

50. Crews P Harrison B. New triterpene-ketides (merotriterpenes), haliclotriol a and b, from an indo-pacific *haliclona* sponge. Tetrahedron. 2000;56:9039-9046.

51. Aoki S, Matsui K, Tanaka K, Satari R, Kobayashi M. Lembehyne a, a novel neuritogenic polyacetylene, from a marine sponge of *haliclona* sp. Tetrahedron. 2000;56:9945-9948.

52. Aoki S, Cao L, Matsui K, Rachmat R, Akiyama S-i, Kobayashi M. Kendarimide a, a novel peptide reversing p-glycoprotein-mediated multidrug resistance in tumor cells, from a marine sponge of *haliclona* sp. Tetrahedron. 2004;60:7053-7059.

53. Aratake S, Trianto A, Hanif N, de Voogd NJ, Tanaka J. A new polyunsaturated brominated fatty acid from a *haliclona* sponge. Mar. Drugs. 2009;7:523-527.

54. Zhang H, Loveridge ST, Tenney K, Crews P. A new 3-alkylpyridine alkaloid from the marine sponge *haliclona* sp. And its cytotoxic activity. Nat Prod Res. 2015;30:1262–1265.

55. Trianto A, Hermawan I, de Voogd NJ, Tanaka J. Halioxepine, a new meroditerpene from an indonesian sponge *haliclona* sp. Chem. Pharm. Bull. 2011;59:1311-1313.

56. Yamazaki H, Wewengkang DS, Kanno S-i, Ishikawa M, Rotinsulu H, Mangindaan REP, Namikoshi M. Papuamine and haliclonadiamine, obtained from an indonesian sponge *haliclona* sp., inhibited cell proliferation of human cancer cell lines. Nat. Prod. Res. 2013;27:1012-1015.

57. Craig KS, Williams DE, Hollander I, Frommer E, Mallon R, Collins K, Wojciechowicz D, Tahir A, Van Soest R, Andersen RJ. Novel sesterterpenoid and norsesterterpenoid rce-protease inhibitors isolated from the marine sponge *hippospongia* sp. Tetrahedron Letters. 2002;43:4801-4804.

58. Carney JR, Scheuer PJ, Kelly-Borges M. Makaluvamine g, a cytotoxic pigment from an indonesian sponge *histodermella* sp. Tetrahedron. 1993;49:8483-6.

59. Ghosh AK, Wang Y, Kim JT. Total synthesis of microtubule-stabilizing agent (-)-laulimalide. Journal of Organic Chemistry. 2001;66:8973-8982.

60. Shimizu A Nishiyama S. Synthesis of the c1-c16 fragment of the marine toxin, laulimalide. Tetrahedron Letters. 1997;38:6011-6014.

61. Moore RE, Banarjee S, Bornemann V, Caplan FR, Chen JL, Corley DG, Larsen LK, Moore BS, Patterson GML, et al. Novel cytotoxins and fungicides from blue-green algae and marine animals possessing algal symbionts. Pure and Applied Chemistry. 1989;61:521-4.

62. Corley DG, Herb R, Moore RE, Scheuer PJ, Paul VJ. Laulimalides. New potent cytotoxic macrolides from a marine sponge and a nudibranch predator. Journal of Organic Chemistry. 1988;53:3644-6.

63. Abdjul DB, Yamazaki H, Takahashi O, Kirikoshi R, Mangindaan REP, Namikoshi M. Two new protein tyrosine phosphatase 1b inhibitors, hyattellactones a and b, from the indonesian marine sponge *hyattella* sp. Bioorg. Med. Chem. Lett. 2015;25:904-907.

64. Enders D Schusseler T. First highly efficient asymmetric synthesis of the *hyrtios erectus* diketotriterpenoid. Synthesis. 2002;2002:2280-2288.

65. Williams DE, Tahir A, Andersen RJ. A new acyclic diketotriterpenoid isolated from the indonesian marine sponge *hyrtios erectus*. Journal of Natural Products. 1999;62:653-654.

66. Pina IC, Sanders ML, Crews P. Puupehenone congeners from an indo-pacific *hyrtios* sponge. Journal of Natural Products. 2003;66:2-6.

67. Mau CMS, Nakao Y, Yoshida WY, Scheuer PJ, Kelly-Borges M. Waiakeamide, a cyclic hexapeptide from the sponge *ircinia dendroides*. Journal of Organic Chemistry. 1996;61:6302-6304.

68. Fernández R, Bayu A, Aryono Hadi T, Bueno S, Pérez M, Cuevas C, Yunovilsa Putra M Unique polyhalogenated peptides from the marine sponge *ircinia* sp. Marine Drugs, 2020. **18**, 396 DOI: 10.3390/md18080396.

69. Ebada Sherif S, Wray V, de Voogd Nicole J, Deng Z, Lin W, Proksch P. Two new jaspamide derivatives from the marine sponge *jaspis splendens*. Mar. Drugs. 2009;7:434-44.

70. Setyowati EP, Jenie UA, Sudarsono, Kardono LBS, Rahmat R. Identification of cytotoxic constituent of indonesian sponge *kaliapsis* sp. (bowerbank). Pak. J. Biol. Sci. 2008;11:2560-2566.

71. Setyowati EP, Jenie UA, Sudarsono, Kardono LBS, Rahmat R. Theonellapeptolide id: Structure identification of cytotoxic constituent from *kaliapsis* sp. Sponge (bowerbank) collected from west bali sea indonesia. J. Biol. Sci. (Faisalabad, Pak.). 2009;9:29-36.

72. Hanif N, Tanaka J, Setiawan A, Trianto A, de Voogd NJ, Murni A, Tanaka C, Higa T. Polybrominated diphenyl ethers from the indonesian sponge *lamellodysidea herbacea*. Journal of Natural Products. 2007;70:432-435.

73. Torii M, Kato H, Hitora Y, Angkouw ED, Mangindaan REP, de Voogd NJ, Tsukamoto SA-OX. Lamellodysidines a and b, sesquiterpenes isolated from the marine sponge *lamellodysidea herbacea*. J. Nat. Prod. 2017;80:2536–2541.

74. Faisal MR, Kellermann MY, Rohde S, Putra MY, Murniasih T, Risdian C, Mohr KI, Wink J, Praditya DF, Steinmann E, Koeck M, Schupp PJ. Ecological and pharmacological activities of polybrominated diphenyl ethers (pbdes) from the indonesian marine sponge *lamellodysidea herbacea*. Mar. Drugs. 2021;19:611.

75. Ohte S, Yamazaki H, Takahashi O, Rotinsulu H, Wewengkang DS, Sumilat DA, Abdjul DB, Maarisit W, Kapojos MM, Zhang H, Hayashi F, Namikoshi M, Katagiri T, Tomoda H, Uchida R. Inhibitory effects of sesquiterpene lactones from the indonesian marine sponge *lamellodysidea* cf. *Herbacea* on bone morphogenetic protein-induced osteoblastic differentiation. Bioorg. Med. Chem. Lett. 2021;35:127783.

76. Kotoku N, Hiramatsu A, Tsujita H, Hirakawa Y, Sanagawa M, Aoki S, Kobayashi M. Structure-activity relationships study of bastadin 6, an anti-angiogenic brominated-tyrosine derived metabolite from marine sponge. Archiv der Pharmazie (Weinheim, Germany). 2008;341:568-577.

77. Dai J, Liu Y, Zhou Y-D, Nagle DG. Cytotoxic metabolites from an indonesian *sponge lendenfeldia* sp. Journal of Natural Products. 2007;70:1824-1826.

78. Hassan W, Edrada R, Ebel R, Wray V, Berg A, Van Soest R, Wiryowidagdo S, Proksch P. New imidazole alkaloids from the indonesian sponge *leucetta chagosensis*. Journal of Natural Products. 2004;67:817-822.

79. Tsukamoto S, Kawabata T, Kato H, Ohta T, Rotinsulu H, Mangindaan REP, van Soest RWM, Ukai K, Kobayashi H, Namikoshi M. Naamidines h and i, cytotoxic imidazole alkaloids from the indonesian marine sponge *leucetta chagosensis*. Journal of Natural Products. 2007;70:1658-1660.

80. Ushiyama S, Umaoka H, Kato H, Suwa Y, Morioka H, Rotinsulu H, Losung F, Mangindaan REP, de Voogd NJ, Yokosawa H, Tsukamoto S. Manadosterols a and b, sulfonated sterol dimers inhibiting the ubc13-uev1a interaction, isolated from the marine sponge *lissodendryx fibrosa*. J. Nat. Prod. 2012;75:1495-1499.

81. Park SK, Kim SS, Park JD, Hong JS, Kim IK. A study on the chemical constituents from marine sponge *luffariella* sp. Journal of the Korean Chemical Society. 1995;39:559-63.

82. Sakai E, Kato H, Rotinsulu H, Losung F, Mangindaan REP, Voogd NJ, Yokosawa H, Tsukamoto S. Variabines a and b: New *β*-carboline alkaloids from the marine sponge *luffariella variabilis*. J. Nat. Med. 2014;68:215-219.

83. Dai H-F, Edrada RA, Ebel R, Nimtz M, Wray V, Proksch P. Norlanostane triterpenoidal saponins from the marine sponge *melophlus sarassinorum*. Journal of Natural Products. 2005;68:1231-1237.

84. Sadahiro Y, Hitora Y, Fukumoto A, Ise Y, Angkouw ED, Mangindaan REP, Tsukamoto S. Melophluosides a and b, new triterpene galactosides from the marine sponge *melophlus sarasinorum*. Tetrahedron Letters. 2020;61:151852.

85. Suna H, Aoki S, Setiawan A, Kobayashi M. Crambescidin 800, a pentacyclic guanidine alkaloid, protects a mouse hippocampal cell line against glutamate-induced oxidative stress. Journal of Natural Medicines. 2007;61:288-295.

86. Hertiani T, Edrada RA, van Soest RWM, Sudarsono, Mueller WEG, Proksch P. Chemical investigation on indonesian marine sponge *mycale phyllophila*. Maj. Farm. Indones. 2009;20:104-111.

87. Liang Z, Sulzmaier FJ, Yoshida WY, Kelly M, Ramos JW, Williams PG. Neopetrocyclamines a and b, polycyclic diamine alkaloids from the sponge *neopetrosia cf exigua*. J. Nat. Prod. 2015;78:543-547.

88. Kato H, Nehira T, Matsuo K, Kawabata T, Kobashigawa Y, Morioka H, Losung F, Mangindaan REP, de Voogd NJ, Yokosawa H, Tsukamoto S. Niphateolide a: Isolation from the marine sponge *niphates olemda* and determination of its absolute configuration by an ecd analysis. Tetrahedron. 2015;71:6956-6960.

89. Ichiba T, Scheuer PJ, Kelly-Borges M. Sponge-derived polyunsaturated c16 di- and tribromocarboxylic acids. Helvetica Chimica Acta. 1993;76:2814-2816.

90. Ali AA, Hassanean HA, Elkhayat ES, Edrada RA, Ebel R, Proksch P. Imidazole alkaloids from the indopacific sponge *pericharax heteroraphis*. Bulletin of Pharmaceutical Sciences, Assiut University. 2007;30:149-157.

91. Ashour M, Edrada-Ebel R, Ebel R, Wray V, van Soest RWM, Proksch P. New purine derivatives from the marine sponge *petrosia nigricans*. Nat. Prod. Commun. 2008;3:1889-1894.

92. Maarisit W, Yamazaki H, Kanno S-i, Tomizawa A, Rotinsulu H, Wewengkang DS, Sumilat DA, Ukai K, Kapojos MM, Namikoshi M. A tetramic acid derivative with protein tyrosine phosphatase 1b inhibitory activity and a new nortriterpene glycoside from the indonesian marine sponge *petrosia* sp. Bioorganic & Medicinal Chemistry Letters. 2017;27:999-1002.

93. Roy MC, Tanaka J, de Voogd N, Higa T. New scalarane class sesterterpenes from an indonesian sponge, *phyllospongia* sp. Journal of Natural Products. 2002;65:1838-1842.

94. Juagdan EG, Kalidindi RS, Scheuer PJ, Kelly-Borges M. Elenic acid, an inhibitor of topoisomerase ii, from a sponge, *plakinastrella* sp. Tetrahedron Letters. 1995;36:2905-2908.

95. Chianese G, Scala F, Calcinai B, Cerrano C, Dien HA, Kaiser M, Tasdemir D, Taglialatela-Scafati O. Natural and semisynthetic analogues of manadoperoxide b reveal new structural requirements for trypanocidal activity. Mar. Drugs. 2013;11:3297-3308, 12 pp.

96. Costantino V, Della Sala G, Saurav K, Teta R, Bar-Shalom R, Mangoni A, Steindler L. Plakofuranolactone as a quorum quenching agent from the indonesian sponge *plakortis* cf. *Lita*. Mar. Drugs. 2017;15:59.

97. Fattorusso C, Persico M, Calcinai B, Cerrano C, Parapini S, Taramelli D, Novellino E, Romano A, Scala F, Fattorusso E, Taglialatela-Scafati O. Manadoperoxides a-d from the indonesian sponge *plakortis* cfr. *Simplex*. Further insights on the structure-activity relationships of simple 1,2-dioxane antimalarials. J. Nat. Prod. 2010;73:1138-1145.

98. Pettit GR, Nogawa T, Knight JC, Doubek DL, Hooper JNA. Antineoplastic agents. 535. Isolation and structure of plakorstatins 1 and 2 from the indo-pacific sponge *plakortis nigra*. Journal of Natural Products. 2004;67:1611-1613.

99. Ichiba T, Scheuer PJ, Kelly-Borges M. Two cytotoxic 3,6-epidioxy fatty acids from an indonesian sponge, *plakortis* sp. Tetrahedron. 1995;51:12195-202.

100. Ohtani II, Ichiba T, Isobe M, Kelly-Borges M, Scheuer PJ. Kauluamine: An unprecedented manzamine dimer from an indonesian marine sponge *prianos* sp. Tennen Yuki Kagobutsu Toronkai Koen Yoshishu. 1995;117:10743-10744.

101. Hertiani T, Edrada R, van Soest RWM, Mueller WEG, Sudarsono, Proksch P. Chemical investigation on *pseudoceratina purpurea* collected from banyuwangi indonesia. Maj. Farm. Indones. 2009;20:17-26.

102. Rashid MA, Gustafson KR, Cartner LK, Shigematsu N, Pannell LK, Boyd MR. Microspinosamide, a new hiv-inhibitory cyclic depsipeptide from the marine sponge *sidonops microspinosa*. Journal of Natural Products. 2001;64:117-121.

103. Parrish SM, Yoshida WY, Kondratyuk TP, Park E-J, Pezzuto JM, Kelly M, Williams PG. Spongiapyridine and related spongians isolated from an indonesian *spongia* sp. J. Nat. Prod. 2014;77:1644-1649.

104. Tan LT, Williamson RT, Gerwick WH, Watts KS, McGough K, Jacobs R. Cis,cis- and trans,trans-ceratospongamide, new bioactive cyclic heptapeptides from the indonesian red alga *ceratodictyon spongiosum* and symbiotic sponge *sigmadocia symbiotica*. Journal of Organic Chemistry. 2000;65:419-425.

105. Plaza A, Bifulco G, Keffer JL, Lloyd JR, Baker HL, Bewley CA. Celebesides a-c and theopapuamides b-d, depsipeptides from an indonesian sponge that inhibit hiv-1 entry. J. Org. Chem. 2009;74:504-512.

106. Jimenez JI, Yoshida WY, Scheuer PJ, Kelly M. Scalarane-based sesterterpenes from an indonesian sponge *strepsichordaia aliena*. Journal of Natural Products. 2000;63:1388-1392.

107. Jimenez JI, Yoshida WY, Scheuer PJ, Lobkovsky E, Clardy J, Kelly M. Honulactones: New bishomoscalarane sesterterpenes from the indonesian sponge *strepsichordaia aliena*. Journal of Organic Chemistry. 2000;65:6837-6840.

108. Ibrahim SRM, Mohamed GA, Elkhayat ES, Gouda YG, Proksch P. Strepsiamide a-c, new ceramides from the marine sponge *strepsichordaia lendenfeldi*. Natural Product Communications. 2008;3:205-209.

109. Eder C, Proksch P, Wray V, Steube K, Bringmann G, Van Soest RWM, Sudarsono, Ferdinandus E, Pattisina LA, Wiryowidagdo S, Moka W. New alkaloids from the indopacific sponge *stylissa carteri*. Journal of Natural Products. 1999;62:184-187.

110. Ebada SS, Linh MH, Longeon A, de Voogd NJ, Durieu E, Meijer L, Bourguet-Kondracki M-L, Singab ANB, Mueller WEG, Proksch P. Dispacamide e and other bioactive bromopyrrole alkaloids from two indonesian marine sponges of the genus *stylissa*. Nat. Prod. Res. 2015;29:231-238.

111. Arai M, Yamano Y, Fujita M, Setiawan A, Kobayashi M. Stylissamide x, a new proline-rich cyclic octapeptide as an inhibitor of cell migration, from an indonesian marine sponge of *stylissa* sp. Bioorg. Med. Chem. Lett. 2012;22:1818-1821.

112. Fouad MA, Debbab A, Wray V, Muller WEG, Proksch P. New bioactive alkaloids from the marine sponge *stylissa* sp. Tetrahedron. 2012;68:10176-10179.

113. Bonnington LS, Tanaka J, Higa T, Kimura J, Yoshimura Y, Nakao Y, Yoshida WY, Scheuer PJ. Cupolamide a: A cytotoxic cyclic heptapeptide from two samples of the sponge *theonella cupola*. Journal of Organic Chemistry. 1997;62:7765-7767.

114. Ichiba T, Nakao Y, Scheuer PJ, Sata NU, Kelly-Borges M. Kumusine, a chloroadenine riboside from a sponge, *theonella* sp. Tetrahedron Letters. 1995;36:3977-80.

115. Roy MC, Ohtani II, Ichiba T, Tanaka J, Satari R, Higa T. New cyclic peptides from the indonesian sponge *theonella swinhoei*. Tetrahedron. 2000;56:9079-9092.

116. Angawi RF, Calcinai B, Cerrano C, Dien HA, Fattorusso E, Scala F, Taglialatela-Scafati O. Dehydroconicasterol and aurantoic acid, a chlorinated polyene derivative, from the indonesian sponge *theonella swinhoei*. J. Nat. Prod. 2009;72:2195-2198.

117. Roy MC, Ohtani II, Tanaka J, Higa T, Satari R. Barangamide a, a new cyclic peptide from the indonesian sponge *theonella swinhoei*. Tetrahedron Letters. 1999;40:5373-5376.

118. Sinisi A, Calcinai B, Cerrano C, Dien HA, Zampella A, D'Amore C, Renga B, Fiorucci S, Taglialatela-Scafati O. New tridecapeptides of the theonellapeptolide family from the indonesian sponge *theonella swinhoei*. Beilstein J. Org. Chem. 2013;9:1643-1651, 9 pp.

119. Sirirath S, Tanaka J, Ohtani II, Ichiba T, Rachmat R, Ueda K, Usui T, Osada H, Higa T. Bitungolides a-f, new polyketides from the indonesian sponge *theonella* cf. *Swinhoei*. Journal of Natural Products. 2002;65:1820-1823.

120. Nagasawa Y, Ueoka R, Yamanokuchi R, Horiuchi N, Ikeda T, Rotinsulu H, Mangindaan REP, Ukai K, Kobayashi H, Namikoshi M, Hirota H, Yokosawa H, Tsukamoto S. Isolation of salsolinol, a tetrahydroisoquinoline alkaloid, from the marine sponge *xestospongia* cf. *Vansoesti* as a proteasome inhibitor. Chem. Pharm. Bull. 2011;59:287-290.

121. Dai J, Sorribas A, Yoshida WY, Kelly M, Williams PG. Xestosaprols from the indonesian marine sponge *xestospongia* sp. J. Nat. Prod. 2010;73:1188-1191.

122. Millan-Aguinaga N, Soria-Mercado IE, Williams P. Xestosaprol d and e from the indonesian marine sponge *xestospongia* sp. Tetrahedron Lett. 2010;51:751-753.

123. He F, Mai LH, Longeon A, Copp BR, Loaeec N, Bescond A, Meijer L, Bourguet-Kondracki M-L. Novel adociaquinone derivatives from the indonesian sponge *xestospongia* sp. Mar. Drugs. 2015;13:2617-2628.

124. Calcul L, Longeon A, Al-Mourabit A, Guyot M, Bourguet-Kondracki M-L. Novel alkaloids of the aaptamine class from an indonesian marine sponge of the genus *xestospongia*. Tetrahedron. 2003;59:6539-6544.

125. Murtihapsari M, Salam S, Kurnia D, Darwati D, Kadarusman K, Abdullah FF, Herlina T, Husna MH, Awang K, Shiono Y, Azmi MN, Supratman U. A new antiplasmodial sterol from indonesian marine sponge, *xestospongia* sp. Nat. Prod. Res. 2021;35:937-944.

126. Latifah La, Soekamto Nh, Tahir A. New antibacterial activities of brominated c18 and c20 fatty acids isolated from marine sponge *xestospongia testudinaria* against shrimp pathogenic bacteria. Rasayan J. Chem. 2021;14:460-465.

127. Lin W, Brauers G, Ebel R, Wray V, Berg A, Sudarsono, Proksch P. Novel chromone derivatives from the fungus *aspergillus versicolor* isolated from the marine sponge *xestospongia exigua*. J. Nat. Prod. 2003;66:57-61.

128. Edrada RA, Heubes M, Brauers G, Wray V, Berg A, Graefe U, Wohlfarth M, Muehlbacher J, Schaumann K, Sudarsono, Bringmann G, Proksch P. Online analysis of xestodecalactones a-c, novel bioactive metabolites from the fungus *penicillium* cf. *Montanense* and their subsequent isolation from the sponge *xestospongia exigua*. Journal of Natural Products. 2002;65:1598-1604.

129. Smith CJ, Hettich RL, Jompa J, Tahir A, Buchanan MV, Ireland CM. Cadiolides a and b, new metabolites from an ascidian of the genus *botryllus*. J. Org. Chem. 1998;63:4147-4150.

130. Fernandez R, Martin MJ, Rodriguez-Acebes R, Reyes F, Francesch A, Cuevas C. Diazonamides c-e, new cytotoxic metabolites from the ascidian *diazona* sp. Tetrahedron Lett. 2008;49:2283-2285.

131. Copp BR, Jompa J, Tahir A, Ireland CM. Styelsamines a-d: New tetracyclic pyridoacridine alkaloids from the indonesian ascidian *eusynstyela latericius*. J. Org. Chem. 1998;63:8024-8026.

132. Yamazaki H, Wewengkang DS, Nishikawa T, Rotinsulu H, Mangindaan REP, Namikoshi M. Two new tryptamine derivatives, leptoclinidamide and (-)-leptoclinidamine b, from an indonesian ascidian *leptoclinides dubius*. Mar. Drugs. 2012;10:349-357.

133. Nakazawa T, Xu J, Nishikawa T, Oda T, Fujita A, Ukai K, Mangindaan REP, Rotinsulu H, Kobayashi H, Namikoshi M. Lissoclibadines 4-7, polysulfur aromatic alkaloids from the indonesian ascidian *lissoclinum* cf. *Badium*. J. Nat. Prod. 2007;70:439-442.

134. Wang W, Takahashi O, Oda T, Nakazawa T, Ukai K, Mangindaan REP, Rotinsulu H, Wewengkang DS, Kobayashi H, Tsukamoto S, Namikoshi M. Lissoclibadins 8-14, polysulfur dopamine-derived alkaloids from the colonial ascidian *lissoclinum* cf. *Badium*. Tetrahedron. 2009;65:9598-9603.

135. Oda T, Lee J-S, Sato Y, Kabe Y, Sakamoto S, Handa H, Mangindaan REP, Namikoshi M. Inhibitory effect of *n,n*-didesmethylgrossularine-1 on inflammatory cytokine production in lipopolysaccharide-stimulated raw 264.7 cells. Mar. Drugs. 2009;7:589-599.

136. Wang W, Oda T, Fujita A, Mangindaan REP, Nakazawa T, Ukai K, Kobayashi H, Namikoshi M. Three new sulfur-containing alkaloids, polycarpaurines a, b, and c, from an indonesian ascidian *polycarpa aurata*. Tetrahedron. 2007;63:409-412.

137. Tatsuta T, Hosono M, Rotinsulu H, Wewengkang DS, Sumilat DA, Namikoshi M, Yamazaki H. Lissoclibadin 1, a polysulfur aromatic alkaloid from the indonesian ascidian *lissoclinum* cf. *Badium*, induces caspase-dependent apoptosis in human colon cancer cells and suppresses tumor growth in nude mice. J. Nat. Prod. 2017;80:499-502.

138. Urda C, Fernandez R, Rodriguez J, Perez M, Jimenez C, Cuevas C. Bistratamides m and n, oxazole-thiazole containing cyclic hexapeptides isolated from *lissoclinum bistratum* interaction of zinc (ii) with bistratamide k. Mar. Drugs. 2017;15:1-11.

139. Casertano M, Imperatore C, Luciano P, Aiello A, Putra MY, Gimmelli R, Ruberti G, Menna M. Chemical investigation of the indonesian tunicate *polycarpa aurata* and evaluation of the effects against *schistosoma mansoni* of the novel alkaloids polyaurines a and b. Mar. Drugs. 2019;17:278.

140. Sibero MT, Trianto A, Frederick EH, Wijaya AP, Ansori ANM, Igarashi Y. Biological activities and metabolite profiling of *polycarpa aurata* (tunicate, ascidian) from barrang caddi, spermonde archipelago, indonesia. Jordan J. Biol. Sci. 2022;15:15-20.

141. Rodriguez J, Nieto RM, Jimenez C. New briarane stecholide diterpenes from the indonesian gorgonian *briareum* sp. J. Nat. Prod. 1998;61:313-317.

142. Gonzalez N, Barral MA, Rodriguez J, Jimenez C. New cytotoxic steroids from the gorgonian *isis hippuris*. Structure-activity studies. Tetrahedron. 2001;57:3487-3497.

143. Garcia M, Rodriguez J, Jimenez C. Absolute structures of new briarane diterpenoids from *junceella fragilis*. J. Nat. Prod. 1999;62:257-260.

144. Prasedya ES MA, Candra Dwipayana Hamdin, Aluh Nikmatullah, Susumu Yoshie, Masao Miyake, Daisuke Kobayashi, Akihiro Hazama, Haji Sunarpi. Evaluation of indonesian selected macroalgae for their antitumor and cytoprotective activity. Vol. Volume: 8. 2018: ssue: 11. 123-130.

145. Sunarpi H, Prasedya E, Ariyana M, Nikmatullah A, Zulkifli L, Yoshie S, Miyake M, Kobayashi D, Hazama A. Cytotoxicity and antiproliferative activity of indonesian red algae *acanthophora muscoides* crude ethanol extracts. Journal of Biological Sciences. 2018;18:425-433.

146. Anam C, Chasanah E, Perdhana BP, Fajarningsih ND, Yusro NF, Sari AM, Nursiwi A, Praseptiangga D, Yunus A. Cytotoxicity of crude lectins from red macroalgae from the southern coast of java island, gunung kidul regency, yogyakarta, indonesia. IOP Conference Series: Materials Science and Engineering. 2017;193:012017.

147. Arsianti A, Aziza YAN, Kurniasari KD, Mandasari BKD, Masita R, Zulfa FR, Dewi MK, Zagloel CRZ, Azizah NN, Putrianingsih R. Phytochemical test and cytotoxic activity of macroalgae *eucheuma cottonii* against cervical hela cells. Pharmacognosy Journal. 2018;10:1012-1017.

148. Sofiana MSJ, Safitri I, Warsidah W, Helena S, Nurdiansyah SI. Antioxidant and anti-inflammatory activities from ethanol extract of *eucheuma cottonii* from lemukutan island waters west kalimantan. Saintek Perikanan: Indonesian Journal of Fisheries Science and Technology. 2021;17:247-253.

149. Khatulistiani TS, Noviendri D, Munifah I, Melanie S. Bioactivities of red seaweed extracts from banten, indonesia. IOP Conference Series: Earth and Environmental Science. 2020;404:012065.

150. Kasanah N, Amelia W, Mukminin A, Triyanto, Isnansetyo A. Antibacterial activity of indonesian red algae *gracilaria edulis* against bacterial fish pathogens and characterization of active fractions. Nat Prod Res. 2019;33:3303-3307.

151. Pattipeilohy F. MT, Mailoa M.N., Sormin, R.B.D. Antibacterial activity of seaweed (*gymnogongrus* sp) extract against *salmonella typhimurium*, *escherichia coli* and *bacillus subtilis*. Int J Sci Res Publ. 2017;7.

152. Dini I, Soekamto NH, Firdaus, Supratman U. Antibacterial and cytotoxic activities assay from the extract of macroalga *halimeda cylindracea* from gulf of boni, indonesia. Journal of Physics: Conference Series. 2019;1341:032035.

153. Ahmad A. Isolation and characterization of bioactive protein from green algae *halimeda macrobola* acting as antioxidant and anticancer agent. American Journal of Biomedical and Life Sciences. 2014;2:134.

154. Widowati R, Handayani S, Suprihatin, Rahayu I, Antara N. Antibacterial activity of methanol extract of *gracilaria salicornia*, *halimeda gracilis*, *halimeda macroloba*, and *hypnea asperi* from indonesia. International Journal of Advanced Science and Technology. 2019;129:67-80.

155. Santoso J, Podungge F, Sumaryanto H. Chemical composition and antioxidant activity of tropical brown algae *padina australis* from pramuka island, district of seribu island, indonesia. Jurnal Ilmu dan Teknologi Kelautan Tropis. 2013;5:287-297.

156. Singkoh M. F. O. KDY, Rumondor M. J. Phytochemical screening and antibacterial activity of brown algae (*padina australis*) from atep oki coast, east lembean of minahasa regency. AACL Bioflux. 2021;14:455-461.

157. Joice P. M. Kolanus aEJD. Inhibitory test antimicrobial of seaweed extract from *padina* sp. Against the growth of *vibrio parahaemolyticus*, *staphylococcus aureus*, *escherichia coli* and *salmonella thypimirium*. Proceedings of the 3^rd^ International Seminar of Basic Sciences. 2017;1:35-44.

158. War Naw S, Darli Kyaw Zaw N, Siti Aminah N, Amin Alamsjah M, Novi Kristanti A, Nege AS, Thanda Aung H. Bioactivities, heavy metal contents and toxicity effect of macroalgae from two sites in madura, indonesia. Journal of the Saudi Society of Agricultural Sciences. 2020;19:528-537.

159. Prasedya ES, Martyasari NWR, Abidin AS, Pebriani SA, Ilhami BTK, Frediansyah A, Sunarwidhi AL, Widyastuti S, Sunarpi H. Macroalgae *sargassum cristaefolium* extract inhibits proinflammatory cytokine expression in balb/c mice. Scientifica (Cairo). 2020;2020:9769454.

160. Prasedya ES, Martyasari NWR, Abidin AS, Ilhami BTK, Padmi H, Widyastuti S, Sunarwidhi AL, Sunarpi H. Antioxidant activity of brown macroalgae *sargassum* ethanol extract from lombok coast, indonesia. IOP Conference Series: Earth and Environmental Science. 2021;712:012038.

161. Prasedya ES, Frediansyah A, Martyasari NWR, Ilhami BK, Abidin AS, Padmi H, Fahrurrozi, Juanssilfero AB, Widyastuti S, Sunarwidhi AL. Effect of particle size on phytochemical composition and antioxidant properties of *sargassum cristaefolium* ethanol extract. Scientific Reports. 2021;11:17876.

162. Scania A Chasani A. The anti-bacterial effect of phenolic compounds from three species of marine macroalgae. Biodiversitas Journal of Biological Diversity. 2021;22:3412-3417.

163. Singkoh MFO, Mantiri DMH, Lumenta C, Manoppo H. Biomineral characterization and antibacterial activity of marine algae *tricleocarpa fragilis* from kora-kora coastal waters of minahasa regency, indonesia. AACL Bioflux. 2019;12:1814-1822.

164. Prasedya ES, Martyasari NWR, Apriani R, Mayshara S, Fanani RA, Sunarpi H. Antioxidant activity of *ulva lactuca* l. From different coastal locations of lombok island, indonesia. AIP Conference Proceedings. 2019;2199:020003.

165. Arguelles EDLR B. Sapin A. Nutrient composition, antioxidant and antibacterial activities of *ulva prolifera* o. F. Müller. Squalen Bulletin of Marine and Fisheries Postharvest and Biotechnology. 2021;16:11.

166. Saptiani G, Asikin A, Ardhani F, Hardi E. Mangrove plants species from delta mahakam, indonesia with antimicrobial potency. Biodiversitas. 2018;19:466-471.

167. Husori D, Sumardi, Tarigan H, Gemasih S, Ningsih SR. In vitro anthelmintic activity of *acanthus ilicifolius* leaves extracts on *ascardia galli* and *pheretima posthuma*. Journal of Applied Pharmaceutical Science. 2018;8:164-167.

168. Firdaus M, Prihanto Aa Fau - Nurdiani R, Nurdiani R. Antioxidant and cytotoxic activity of *acanthus ilicifolius* flower. Asian Pacific journal of tropical biomedicine. 2013;3:17–21.

169. Rusyiana R, Lestarini IA, Hamdin CD, Muliasari H. Anticoagulant activity of mangrove (*avicennia alba*) leaves extract in vitro. ILMU KELAUTAN: Indonesian Journal of Marine Sciences. 2021;26:110-116.

170. Arbiastutie Y, Diba F, Masriani M. Short communication: Ethnobotanical and ecological studies of medicinal plants in a mangrove forest in mempawah district, west kalimantan, indonesia. Biodiversitas Journal of Biological Diversity. 2021;22:3164-3170.

171. Andriani S, Widiastuti EL, Nurcahyani N, rosa E, Busman H. Cytotoxic activity of methanol extraction of *avicennia marina* and taurin in the hela cancer cells. Journal of Physics: Conference Series. 2021;1751:012045.

172. Anam K, Susilo D, Kusrini D, Aminin A. Chemical constituents and inhibition xanthine oxidase activity of *avicennia marina* exudate. Research Journal of Medicinal Plants. 2016;11:19-24.

173. Nawar M, Basyuni M, Hanum C, Siregar E. Bioprospecting opportunities of mangrove fruits for the coastal community in lubuk kertang and pulau sembilan, north sumatra, indonesia. Asian Journal of Plant Sciences. 2022;21:145-153.

174. Sari DP, Basyuni M Fau - Hasibuan PAZ, Hasibuan Pa Fau - Sumardi S, Sumardi S Fau - Nuryawan A, Nuryawan A Fau - Wati R, Wati R. Cytotoxic and antiproliferative activity of polyisoprenoids in seventeen mangroves species against widr colon cancer cells. Asian Pacific journal of cancer prevention : APJCP. 2018;19:3393–3400.

175. Manurung J, Kappen J, Schnitzler J, Frolov A, Wessjohann LA, Agusta A, Muellner-Riehl AN, Franke K. Analysis of unusual sulfated constituents and anti-infective properties of two indonesian mangroves, *lumnitzera littorea* and *lumnitzera racemosa* (combretaceae). Separations. 2021;8:1-21.

176. Muhaimin M, Madyawati L, Riski Dwimalida P, Anis Yohana C, Andreas Yoga A, Normalita Eka P, Josephine Elizabeth S. Antiplasmodial activity of methanolic leaf extract of mangrove plants against *plasmodium berghei*. Pharmacognosy Journal. 2019;11:929-935.

177. Harizon, Pujiastuti B, Kurnia D, Sumiarsa D, Shiono Y, Supratman U. Antibacterial triterpenoids from the bark of *sonneratia alba* (lythraceae). Natural Product Communications. 2015;10:277-280.

178. Latief M, Utami A, Amanda H, Muhaimin M, Afifah Z. Antioxidant activity of isolated compound from perepat roots (*sonneratia alba*). Journal of Physics: Conference Series. 2019;1282:012088.

179. Budi SB, Sulistiyati TD, Hardoko H. Phytochemicals and identification of antioxidant compounds from ethanol extract of *sonneratia alba* leaves and bark. Russian Journal of Agricultural and Socio-Economic Sciences. 2019;95:190-196.

180. Halifah P, Yusminah H, Roshanida AR. Phytochemical screening and antimicrobial activity from *sonneratia caseolaris* fruit extract. in Materials Science Forum. 2019. Trans Tech Publ.

181. Pringgenies D, Yudiati E, Widyadmi R, Anggelina A, Syaifudien Bahry M. *Xylocarpus granatum* mangrove fruit extract and sodium alginate extract lotion as potent wound treatment medicine. Jurnal Biologi Papua. 2021;13:67-73.

182. Yusuf S, Astuti R, Batubara I, Chavasiri W. Anti-aging activity of *xylocarpus granatum* phytoextracts and xyloccensins k compound. Indonesian Journal of Pharmacy. 2021;32:365-375.

183. Darmadi J, Batubara RR, Himawan S, Azizah NN, Audah HK, Arsianti A, Kurniawaty E, Ismail IS, Batubara I, Audah KA. Evaluation of indonesian mangrove *xylocarpus granatum* leaves ethyl acetate extract as potential anticancer drug. Scientific reports. 2021;11:6080.

184. Budiarso FS, Elya B, Hanafi M, Limengan AH, Rahmasari R. Antioxidant activity of methanol fractions stem bark of kayu sarampa (*xylocarpus moluccensis* (lam.) m. Roen)). Pharmacognosy Journal. 2021;13:1694-1701.

185. Nugraha AP, Sibero MA-O, Nugraha AP, Puspitaningrum MS, Rizqianti Y, Rahmadhani D, Kharisma VD, Ramadhani NF, Ridwan RD, Noor T, Ernawati DS. Anti-periodontopathogenic ability of mangrove leaves (*aegiceras corniculatum*) ethanol extract: In silico and in vitro study. European journal of dentistry. 2022;17:46–56.

186. Audah KA, Amsyir J, Almasyhur F, Hapsari A, Sutanto H. Development of extract library from indonesian biodiversity: Exploration of antibacterial activity of mangrove *bruguiera cylindrica* leaf extracts. IOP Conference Series: Earth and Environmental Science. 2018;130:012025.

187. Yami ACL, Batubara I, Audah KAJABI. Antioxidant and antibacterial activity of mangrove *brugueira gymnorrhiza* stem extracts against pathogenic bacteria *vibrio cholerae*. Acta Biochimica Indonesiana. 2021;3:53-61.

188. Kurniawaty E, Megaputri S, Mustofa S, Rahmanisa S, Audah KA, Andriani S. Ethanol extract of *bruguiera gymnorrhiza* mangrove leaves and propolis activity on macroscopic healing of cuts in vivo. Acta Biochimica Indonesiana. 2022;5:94.

189. Nurjanah N, Jacoeb A, Hidayat T, Hazar S, Nugraha R. Antioxidant activity, total phenol content, and bioactive components of lindur leave (*bruguierra gymnorrhiza*). American Journal of Food Science and Health. 2016;2:65-70.

190. Lohoo HJ, Berhimpon S, Mantiri DM, Montolalu R. Bioactive components, antibacterial activity, and toxicity of mangrove *bruguiera gymnorrhiza* fruit extract. Aquaculture, Aquarium, Conservation & Legislation. 2021;14:3721-3730.

191. Indriaty I, Ginting B, Hasballah K, Djufri. Assessment cytotoxic assay of rhizophora plants mangrove using brine shrimp (*artemia salina* l) model. IOP Conference Series: Earth and Environmental Science. 2022;951:012070.

192. Istiqomah M, Hasibuan PA, Nuryawan A, Lubis S, Siregar E, Basyuni M. The anticancer compound dolichol from *ceriops tagal* and *rhizophora mucronata* leaves regulates gene expressions in widr colon cancer. Sains Malaysiana. 2021;50:181-189.

193. Kurniawan R. Aktivitas antibakteri ekstrak daun *rhizophora apiculata* terhadap bakteri *edwardsiella tarda* antibacterial activity of *rhizophora apiculata* leaf extract against *edwardsiella tarda* bacteria. Jurnal Natur Indonesia. 2021;19:13-17.

194. Wijaya MD Indraningrat AAG. Antibacterial activity of mangrove root extracts from ngurah rai mangrove forest, denpasar-bali. Biology, Medicine, & Natural Product Chemistry. 2021;10:117-121.

195. Sibero MT, Siswanto AP, Pribadi R, Sabdono A, Radjasa OK, Trianto A, Frederick EH, Wijaya AP, Haryanti D, Triningsih DW. The effect of drying treatment to metabolite profile and cytotoxic potential of *rhizophora apiculata* leaves. Biodiversitas Journal of Biological Diversity. 2020;21:2180-2187.

196. Nanuru EW, Dewi L, Wibowo P. Effect of asiatic mangrove (*rhizophora micronata*) leaves extract as analgesic in male albino ddw mice (*mus musculus* l.) induced by 0, 7% acetic acid. Medical and Health Science Journal. 2021;5:1-8.

197. Purwaningsih S, Salamah E, Sukarno AYP, Deskawati E. Aktivitas antioksidan dari buah mangrove (*rhizophora mucronata* lamk.) pada suhu yang berbeda. Jurnal Pengolahan Hasil Perikanan Indonesia. 2013;16:199-206.

198. Rumengan AP, Mandiangan ES, Tanod WA, Paransa DSJ, Paruntu CP, Mantiri DMH. Identification of pigment profiles and antioxidant activity of *rhizophora mucronata* mangrove leaves origin lembeh, north sulawesi, indonesia. Biodiversitas Journal of Biological Diversity. 2021;22:2805-2816.

199. Suyatno NH, Kirana D, Rofida R, Putri F. Non-phenolic compounds from the stem bark of red mangrove (*rhizophora stylosa*) and evaluation of their cytotoxicity and larvacidal activity. Journal of Biology, Agriculture and Healthcare. 2014;4:65-68.

200. Sumilat DA, Lintang RAJ, Undap SL, Adam AA, Tallei TE. Phytochemical, antioxidant, and antimicrobial analysis of *trichoderma asperellum* isolated from ascidian *eudistoma* sp. J. Appl. Pharm. Sci. 2022;12:90-95.

201. Yamazaki H, Nakayama W, Takahashi O, Kirikoshi R, Izumikawa Y, Iwasaki K, Toraiwa K, Ukai K, Rotinsulu H, Wewengkang DS, Sumilat DA, Mangindaan REP, Namikoshi M. Verruculides a and b, two new protein tyrosine phosphatase 1b inhibitors from an indonesian ascidian-derived *penicillium verruculosum*. Bioorg. Med. Chem. Lett. 2015;25:3087-3090.

202. Sumilat DA, Yamazaki H, Endo K, Rotinsulu H, Wewengkang DS, Ukai K, Namikoshi M. A new biphenyl ether derivative produced by indonesian ascidian-derived *penicillium albobiverticillium*. J. Nat. Med. 2017;71:776-779.

203. Sumilat DA Lintang RAJ. Antibacterial potential of marine fungus *aspergillus nomius* isolated from green algae *bornetella* sp. Jurnal Ilmiah PLATAX. 2021;9:49-49.

204. Nurhalimah S, Rahmawati SI, Hermanianto J, Nurjanah S, Izzati FN, Septiana E, Rachman F, Bustanussalam B, Hapsari Y, Simanjuntak P, Putra MY. Aktivitas antioksidan dari metabolit sekunder kapang endofit mangrove *aegiceras corniculatum*. Biopropal Industri. 2021;12:51-51.

205. Trianto A, Radjasa OK, Subagiyo, Purnaweni H, Bahry MS, Djamaludin R, Tjoa A, Singleton I, Diele K, Evan D. Potential of fungi isolated from a mangrove ecosystem in northern sulawesi, indonesia: Protease, cellulase and anti-microbial capabilities. Biodiversitas. 2021;22:1717-1724.

206. Ayu Gustina Situmorang D Hendri M, Isolasi dan aktivitas antibakteri jamur endofit pada mangrove *avicennia marina* dari pulau payung kabupaten banyuasin sumatera selatan, in Jurnal Penelitian Sains. 2021. p. 125-133.

207. Trifani R, Noverita, Hadi TA, Sinaga E. Antibacterial activity of endosymbiotic fungi isolated from marine sponges collected from kotok kecil island, seribu islands, jakarta. in IOP Conference Series: Earth and Environmental Science. 2021. IOP Publishing Ltd.

208. Lutfiah R, Juliasih NLGR, Hendri J, Setiawan A. Screening extract etoac sponge derived fungi against clinical *staphylococcus aureus* to obtain sustainable natural product. in IOP Conference Series: Earth and Environmental Science. 2021. IOP Publishing Ltd.

209. Pramana AAC, Ramadhani E, Priyambada F, Pertiwi GA, Setiawibawa RAA, Wijayanti N. Sponge-associated fungi isolates from *ancorina* sp. Showed anti-cancer activity against hela cell lines. Journal of Microbiology, Biotechnology and Food Sciences. 2022;12:e3352.

210. Fadillah WN, Sukarno N, Iswantini D, Rahminiwati M, Listiyowati S. New record of *gymnoascus udagawae* associated with *clathria* sp. Sponge from indonesia and the potency as anti-candida. in IOP Conference Series: Earth and Environmental Science. 2021. IOP Publishing Ltd.

211. Bahry MS, Radjasa OK, Trianto A. Potential of marine sponge-derived fungi in the aquaculture system. Biodiversitas. 2021;22:2883-2892.

212. Fadillah WN, Sukarno N, Iswantini D, Rahminiwati M, Hanif N, Waite M. *In vitro* pancreatic lipase inhibition by marine fungi *purpureocillium lilacinum* associated with *stylissa* sp. Sponge as anti-obesity agent. HAYATI Journal of Biosciences. 2022;29:76-86.

213. Handayani DP, Isnansetyo A, Istiqomah I, Jumina J. Anti-vibrio activity of *pseudoalteromonas xiamenensis* stkmti.2, a new potential vibriosis biocontrol bacterium in marine aquaculture. Aquacult. Res. 2022;53:1800-1813.

214. Pringgenies D, Dewi K, Apriliyani P. Isolation and identification of symbiont microorganisms from bioluminescent marine life. Annu. Res. Rev. Biol. 2019;33:1-16.

215. Kristiana R, Bedoux G, Pals G, Mudianta IW, Taupin L, Marty C, Asagabaldan MA, Ayuningrum D, Trianto A, Bourgougnon N, Radjasa OK, Sabdono A, Hanafi M. Bioactivity of compounds secreted by symbiont bacteria of nudibranchs from indonesia. PeerJ. 2020;8:e8093.

216. Ulfah M, Kasanah N, Handayani NSN. Bioactivity and genetic screening of marine actinobacteria associated with red algae *gelidiella acerosa*. Indonesian Journal of Biotechnology. 2018;22:13-21.

217. Wibowo JT, Kellermann MY, Versluis D, Putra MY, Murniasih T, Mohr KI, Wink J, Engelmann M, Praditya DF, Steinmann E, Schupp PJ. Biotechnological potential of bacteria isolated from the sea cucumber *holothuria leucospilota* and *stichopus vastus* from lampung, indonesia. Mar. Drugs. 2019;17:635.

218. Böhringer N, Fisch KM, Schillo D, Bara R, Hertzer C, Grein F, Eisenbarth J-H, Kaligis F, Schneider T, Wägele H, König GM, Schäberle TF. Antimicrobial potential of bacteria associated with marine sea slugs from north sulawesi, indonesia. Front Microbiol. 2017;8:1-8.

219. Pringgenies D Setyati WA. Antifungal strains and gene mapping of secondary metabolites in mangrove sediments from semarang city and karimunjawa islands, indonesia. AIMS Microbiol. 2021;7:499-512.

220. Radjasa OK, Salasia SIO, Sabdono A, Weise J, Imhoff JF, Lammler C, Risk MJ. Antibacterial activity of marine bacterium *pseudomonas* sp. Associated with soft coral *sinularia polydactyla* against *streptococcus equi* subsp. *Zooepidemicus*. Int. J. Pharmacol. 2007;3:170-174.

221. N. Artanti, F. Maryani, H. Mulyani, R.T Dewi, V. Saraswati, Murniasih T. Bioactivities screening of indonesian marine bacteria isolated from sponges. Annales. Bogorienses. 2016;20:25-30.

222. W.F. Safari, E. Chasanah, A.T. Wahyudi. Antibacterial and anticancer activities of marine bacterial extracts and detection of genes for bioactive compounds synthesis. International Journal of Pharmacy and Pharmaceutical Sciences. 2016;8:55-59.

223. Handayani D, Sandrawaty N, Murniati M, Regina R. Screening of endophytic bacteria isolated from marine sponge *haliclona fascigera* for inhibition against clinical isolates of methicillin resistant *staphylococcus aureus* (mrsa). J. Appl. Pharm. Sci. 2015;5:139-142.

224. Sandrawati N, Pariatno R, Suharti N, Handayani D. In vitro cytotoxic activity assay of bacteria extract derived marine sponge *haliclona fascigera* toward hela, widr, t47d, and vero cell line. J. Appl. Pharm. Sci. 2019;9:66-70.

225. Trianto A, Nirwani N, Susanti O, Maesaroh DS, Radjasa OK. The bioactivity of bacterium and fungi living associate with the sponge *reniera* sp. Against multidrug-resistant *staphylococcus aureus* and *escherichia coli*. Biodiversitas Journal of Biological Diversity. 2019;20:2302-2307.

226. Cita YP, Suhermanto A, Radjasa OK, Sudharmono P. Antibacterial activity of marine bacteria isolated from sponge *xestospongia testudinaria* from sorong, papua. Asian Pacific Journal of Tropical Biomedicine. 2017;7:450-454.

227. Rante H, Alam G, Usmar U, Anwar RA, Ali A. Isolation of sponge bacterial symbionts from kodingareng keke island-makassar indonesia which is potential as a producer of antimicrobial compounds. J. Pure Appl. Microbiol. 2022;16:737-743.

228. Nofiani R, Weisberg AJ, Tsunoda T, Panjaitan RGP, Brilliantoro R, Chang JH, Philmus B, Mahmud T. Antibacterial potential of secondary metabolites from indonesian marine bacterial symbionts. Int J Microbiol. 2020;2020:1-11.

229. E.S. Gultom, Hasruddin AFS, A.D. Situmorang, Prasetya E. Identifying sponge symbiont bacterial with antibacterial activity against multi-drug resistant organism (mdro) bacteria from sea waters in sibolga, north sumatra indonesia. BIOSPHERE: Tadris Biology Journal. 2021;12:169-184.

230. Wahyudi AT, Priyanto JA, Maharsiwi W, Astuti RI. Screening and characterization of sponge-associated bacteria producing bioactive compounds anti-*vibrio* sp. American Journal of Biochemistry and Biotechnology. 2018;14:221-229.

231. Setiyono E, Adhiwibawa MAS, Indrawati R, Prihastyanti MNU, Shioi Y, Brotosudarmo THP. An indonesian marine bacterium, *pseudoalteromonas rubra*, produces antimicrobial prodiginine pigments. ACS Omega. 2020;5:4626-4635.

232. Syakti AD, Lestari P, Simanora S, Sari LK, Lestari F, Idris F, Agustiadi T, Akhlus S, Hidayati NV, Riyanti. Culturable hydrocarbonoclastic marine bacterial isolates from indonesian seawater in the lombok strait and indian ocean. Heliyon. 2019;5:e01594.

233. Feliatra F, Batubara UM, Nurulita Y, Lukistyowati I, Setiaji J. The potentials of secondary metabolites from *bacillus cereus* sn7 and *vagococcus fluvialis* ct21 against fish pathogenic bacteria. Microb. Pathog. 2021;158:105062.
